# Detection of bovine milk–, but likely not soy–derived, peptides in human milk after maternal consumption of bovine milk and soy beverage: a randomized, cross-over, dietary intervention trial

**DOI:** 10.3389/fnut.2025.1642177

**Published:** 2025-10-23

**Authors:** Cassandra L. Partridge, Trillitye R. Paullin, Bum Jin Kim, David C. Dallas, Janet E. Williams, Mark A. McGuire, Harpreet Kaur, Michelle K. McGuire

**Affiliations:** ^1^Margaret Ritchie School of Family and Consumer Sciences, University of Idaho, Moscow, ID, United States; ^2^Free to Feed™, Boise, ID, United States; ^3^Nutrition Program, School of Nutrition and Public Health, College of Health, Oregon State University, Corvallis, OR, United States; ^4^Animal, Veterinary and Food Sciences, University of Idaho, Moscow, ID, United States; ^5^Statistical Programs, University of Idaho, Moscow, ID, United States

**Keywords:** nutrition, lactation, allergen, breastfeeding, diet, maternal, infant, peptidomics

## Abstract

**Background:**

Food-borne allergens in human milk (HM) may cause allergic responses in HM-fed infants, but variability of allergen transfer complicates recommendations for individuals nursing food-allergic infants.

**Objective:**

We aimed to identify bovine- and soy-derived peptides in HM after maternal elimination and reintroduction of bovine milk (BM) and soy beverage (SB).

**Methods:**

In this randomized, cross-over, dietary intervention trial, 38 lactating participants underwent 2 study phases, each including a 5-day diet elimination, 3-day diet intervention, and 2-day washout. Each diet intervention required daily consumption of increasing amounts of BM or SB (175, 295, and 415 mL). Peptidomics analysis was performed on a subset of HM samples (24 participants) collected after dietary elimination, and 2 and 4 h after BM/SB consumption (415 mL). Peptides were isolated via ethanol precipitation and C18 solid-phase extraction, analyzed by LC–MS/MS, and identified with Proteome Discoverer.

**Results:**

We identified 121 bovine-derived peptides (associated with 6 proteins) in HM collected during the BM phase. From most to least abundant, these proteins were β-lactoglobulin, *κ*-casein, *α*s1-casein, β-casein, α-lactalbumin protein variant D, and glycosylation-dependent cell adhesion molecule 1. Generalized linear mixed models demonstrated differences in relative abundance for 14 peptides when comparing before, and 2 and 4 h after BM consumption. We identified 8 peptides of possible soy origin in HM collected during the SB phase, but they were not matched to parent proteins with adequate confidence.

**Conclusion:**

The relative abundance of some BM-derived peptides, while low overall, may differ in human milk collected after maternal BM dietary elimination compared to 2 and 4 h after BM consumption. Five days of dietary elimination may not be adequate for the elimination of BM-derived peptides or low levels of these non-human peptides may be present in HM from other sources. No confident soy-derived peptides from the SB were identified in HM after consumption.

**Clinical trial registration:**

https://clinicaltrials.gov/study/NCT04851340, identifier NCT04851340.

## Introduction

Food allergies during infancy and childhood can be life-threatening, severely impacting the health and quality of life of children and their caregivers, and influencing multiple organs and systems throughout the body, including the skin, eyes, respiratory system, cardiovascular system, and gastrointestinal tract (1, 2). More than 7% of US children have a food allergy, and ~40% of those children have multiple food allergies (3). Although the prevalence of food allergies in US infants <1 yr. of age has been estimated to be 3%, with cow’s milk allergy (CMA) and soybean allergy believed to be two of the most prevalent allergies, impacting 1.5 and 0.4% of infants, respectively, parental self-reported rates of food reactivity are as high as 19–35% (3, 4). Furthermore, reactivity to bovine milk and soy often co-occur: 11–14% of infants with an immunoglobulin E (IgE)-mediated CMA are also allergic to foods containing soy (5, 6).

Allergies can occur when the infant consumes an allergenic product directly, but they can also occur in nursing infants whose mothers consume the allergen. Indeed, when consumed by a lactating person, numerous allergens (e.g., proteins and peptides originating from bovine milk (BM), eggs, and peanuts) can be transferred to human milk (HM), leading to a potential allergic response in HM-fed infants (7–11). However, the presence and concentration of non-human peptides and proteins in HM are highly variable within and among lactating individuals (9, 11). Analyses via deep proteome profiling, shotgun proteomics, and parallel reaction monitoring have identified 1,577 human proteins and 36 nonhuman proteins in HM (12), most of the latter being of bovine origin, with αs1-casein being the most abundant. Other bovine proteins in HM include β-casein, *κ*-casein, and β-lactoglobulin. Bovine peptides, specifically β-lactoglobulin and αs1-casein, are frequently detected in milk produced by lactating individuals consuming at least one cup of bovine milk daily (8). Research on the transfer and presence of soy proteins and peptides into HM remains limited.

Management of food allergies throughout the lifespan focuses on avoidance or elimination of specific foods causing the reaction (1). In the first months of life, this strategy provides limited feeding options for many caregivers of infants with CMA and/or soy allergy. Hydrolyzed formulas are available and may be tolerated by many infants with CMA or soy allergy, but are often expensive, and may conflict with the goals of parents who wish to HM-feed their infant. Both the World Health Organization (WHO) and the United Nations Children’s Fund (UNICEF) recommend that children be exclusively fed HM for the first 6 months of life and continue feeding HM, with the addition of complementary foods, for up to 2 yr. and beyond (13, 14). This recommendation is rooted in numerous, well-established, short- and long-term benefits of HM-feeding to both the nursing parent and their infant (15, 16). For parents who wish to exclusively feed HM, management of infant food allergy is therefore generally limited to maternal dietary elimination of any triggering allergens.

Research evaluating the risks of maternal dietary elimination during lactation on maternal health is limited. Highly restrictive elimination diets may lead to nutrient deficiencies, especially when the elimination diet is prolonged (17). Furthermore, some evidence suggests that nursing mothers with food-allergic infants following an elimination diet have higher scores of depression, anxiety, obsession, and anger (18). As described previously, the transfer of proteins from the maternal diet into HM is not consistent across or within individuals regarding quantity and timing (9). The individual variability of protein transfer complicates recommendations regarding specificity and duration of dietary elimination that is needed and impacts recommendations for best practices while nursing infants with food allergies. More information is especially needed related to the transfer of previously unidentified allergens, such as those found in soy, and on factors impacting the variable transfer of these proteins and peptides.

Previous studies evaluating the presence of bovine milk proteins in human milk have frequently used elimination periods of variable lengths ranging from 24 h up to 4 weeks or more (19). Recent research evaluating bovine peptides using nanoflow-HPLC-tandem mass spectrometry has reported clearance of bovine-derived peptides from HM within 6 h (9). We selected a 5-day elimination period as a reasonable balance between minimizing maternal burden and ensuring adequate dietary clearance for the majority of participants.

To help fill the described knowledge gap, the primary objective of this study was to use rigorous analytical methods to identify bovine- and soy-derived peptides in milk produced by nursing individuals after dietary elimination and then reintroduction of these foods. We hypothesized that (i) bovine and soy peptides would not be detectable in milk produced after a 5-d elimination period; (ii) relative abundance of these peptides would be greatest in milk samples collected 4 h after consumption of 415 mL of BM / soy beverage (SB) when compared to samples collected 2 h after consumption of 415 mL bovine or soy beverage and after a 5 d dietary elimination; and (iii) and responses would vary among women.

## Methods

### Study design

This study was a prospective, randomized, longitudinal, cross-over dietary intervention trial. Mother/infant dyads were recruited from Moscow, ID; Boise, ID; New York, NY; and surrounding areas through social media, word-of-mouth, and local advertisements to maternal and child health organizations. Milk samples and data were collected between November 2020 and June 2021, coinciding with the global COVID-19 pandemic. Inclusion criteria required that participants be ≥18 yr. of age, currently nursing or pumping, and ≤12 months postpartum at time of enrollment. Participants were excluded if they were recently diagnosed with COVID-19, experiencing symptoms of COVID-19, or not willing or able to consume SB and/or BM. All participants provided verbal and written informed consent, and procedures were approved by the University of Idaho Institutional Review Board (20–126).

**Figure 1:**
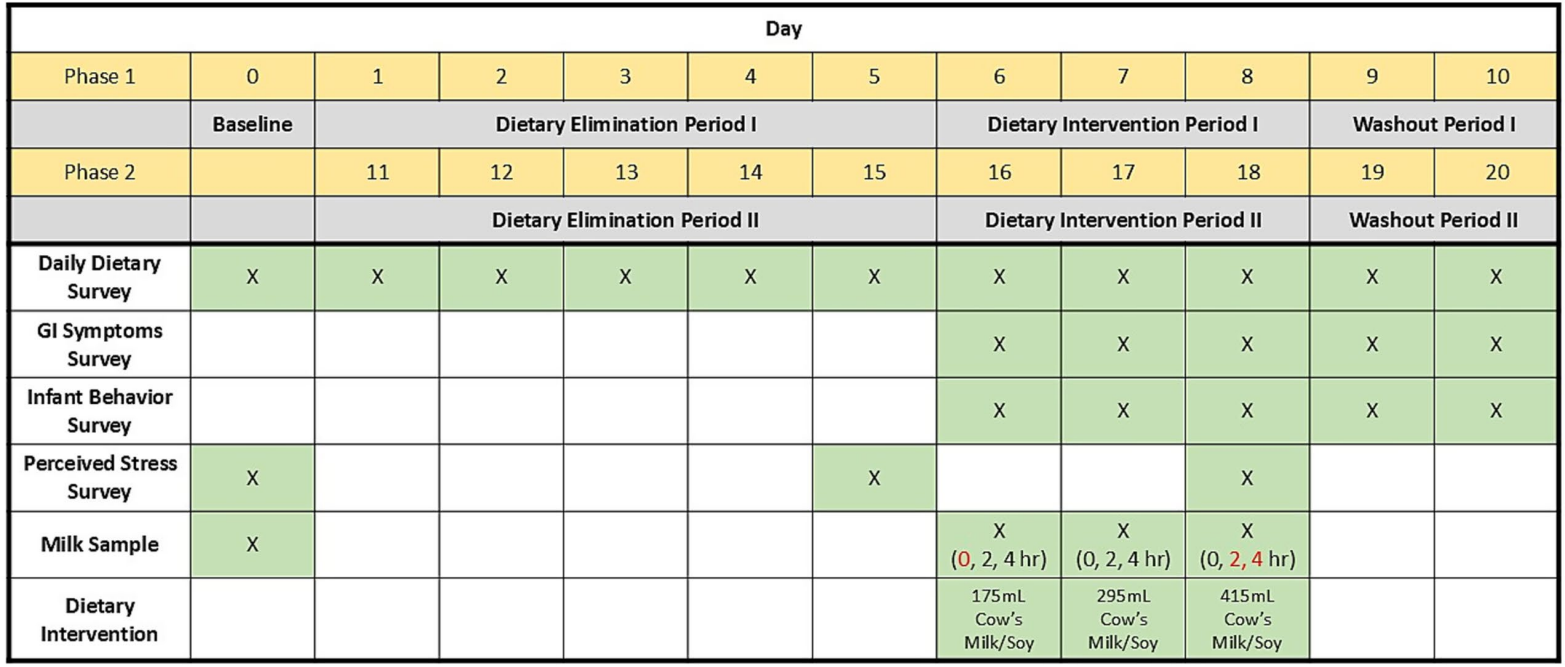
Graphical illustration of sample and data collection regime used in the parent study. Red text highlights human milk samples used for the peptidomic analyses reported herein.

Thirty-eight lactating individuals participated in this cross-over 21-d study that included a baseline collection period (d0) and two 10-d study phases (a BM phase and a soy phase), each consisting of a 5-d dietary elimination period, a 3-d dietary intervention period, and a 2-d washout period (Figure 1). The study sample size was estimated based on funding availability and sample sizes from similarly published research. Participants experienced both a BM and soy phase and were assigned randomly, by the study coordinator, to the order of dietary intervention (BM followed by SB or SB followed by BM) via random number generation in Excel. Baseline human milk samples were collected on d0.

### Dietary interventions

During each 3-d dietary elimination period, participants were instructed to eliminate either all BM-containing products (when followed by the BM reintroduction) or all soy-containing products (when followed by the SB reintroduction). During the entire BM phase (both elimination and reintroduction), participants were instructed not to consume sheep and goat products. Participants were provided a list of foods and ingredients containing BM, during the BM phase, and soy, during the soy phase, and were instructed to check ingredient labels of all products consumed during the study as feasible. A trained research dietitian was available to all participants and provided further resources on diet elimination as needed.

During each of the 3-d dietary intervention periods, participants underwent three consecutive dietary challenges requiring daily consumption of increasing amounts of provided BM or SB [0.75 cup (175 mL), 1.25 cup (295 mL), and 1.75 cup (415 mL)] over the course of 3 days (d6, d7, and d8, respectively). Participants were provided with pasteurized (either High Temperature Short Time or Ultra-high-temperature) 1% fat BM (1 cup provided 100–110 kcals energy and 8–9 g protein) and Pacific Foods Ultra Soy Original SB (1 cup provided 140 kcals energy and 10 g protein) for the appropriate dietary intervention periods. They were also given empty plastic bottles that were pre-measured and labeled with the amount of beverage to be measured by participants and consumed on each day of the dietary intervention.

Participants underwent a 2-d washout period between the end of the first dietary reintroduction phase and the second dietary elimination phase, during which time they were instructed to continue eliminating all other sources of BM or soy during the BM or soy intervention periods as appropriate. Dietary compliance during the study was assessed via printed daily dietary surveys which prompted participants to indicate if, to their knowledge, they successfully eliminated all BM or soy-containing foods (as appropriate for study phase) and provide information on the infant’s daily dietary intake and dietary intake outside of the home, such as at restaurants (Supplementary File S1).

### Milk collection and storage

Human milk was collected by participants in their homes. To reduce contamination, collection kits were aseptically and individually packaged by study personnel wearing masks, gloves, and gowns. Participants were provided detailed written instructions regarding use of aseptic techniques to obtain all samples. The research coordinator for the study verbally reviewed all instructions with participants within 1 week of study initiation and was available during sample collection for virtual assistance as needed.

Participants collected one HM sample during the baseline collection period (d0). During each day of the intervention periods, participants collected a sample of HM (hr 0) and then consumed the provided BM or SB (as appropriate to intervention period) after the first HM collection. In most cases, this was as soon as possible after milk collection, but some women consumed the beverage later in the day. Women collected a second HM sample 2 h after consuming the provided beverage, and a third HM sample 4 h after consuming the provided beverage (Figure 1). At each collection, participants were asked to record if the sample was provided from the left or right breast, the date and time of HM collection, the time since the participant last nursed or pumped, and indicate if they provided a partial or full breast expression (“full” if the breast was fully emptied).

In general, participants were successful following the timing of this study protocol with all but one 2 and 4 h sample collected within 1 h of the study protocol, and the majority collected within 30 min. This variation in sampling timing was not accounted for in statistical analysis. Given the small degree of variability for the majority of our participants and the consistency of timing across study days, we believe this had minimal impact on the study outcomes. Nonetheless, future studies should evaluate the potential effects of post-consumption collection timing more rigorously.

At each collection, participants collected up to 30 mL of HM in sterile collection containers using a provided sterile manual breast pump (Harmony, Medela) or by hand expression. Prior to each milk collection, participants were asked to wash their hands for 20 s using soap and warm water and then clean their breast twice using provided castile cleaning wipes (PDI, New Jersey). Participants were instructed to place samples immediately into their home freezer after collection. All samples were stored frozen in the participant’s freezer for 1–10 d after which time they were transported by participants to a laboratory at the University of Idaho, Boise State University, or Rockefeller University (depending on the subject’s location) in provided food-safe, thermal, foil-lined, soft-sided coolers with frozen ice packs. On arrival at the laboratory, samples were stored at −20 °C. Samples were later thawed, and duplicate 1 mL aliquots were created and frozen at −80 °C prior to further analysis.

### Description of subset of milk samples undergoing peptidomic analyses

Peptidomic analysis was performed on HM (*n* = 144) collected from a subsample of 24 participants in the study population. Subsample inclusion criteria included >80% daily compliance with the dietary elimination and sufficient quantities of HM for analysis. One additional participant was excluded from further analysis after LC-MS/MS due to concern for mislabeling of study samples. Peptidomic analysis was focused on HM collected after the 5-d dietary elimination (d6 and d16; hr. 0), hereafter referred to as “bovine elimination” (BovE) or “soy elimination” (SoyE), and after consumption of BM/SB on the third day of each dietary intervention period (d8 and d18; hr 2 and 4), hereafter referred to as “bovine 2 h” (Bov2Hr), “bovine 4 h” (Bov4Hr), “soy 2 h” (Soy2Hr), or “soy 4 h” (Soy4Hr).

### Peptide extraction and processing

Aliquots of HM (1 mL) were centrifuged at 4 °C for 30 min at 4,000 × g. The aqueous layer (150 μL), which contained the peptides, was transferred to a new tube and the lipid layer and cell pellet reserved and frozen for later analysis. The supernatant was centrifuged again at 4 °C for 30 min at 4,000 × g, and the aqueous layer (75 μL) was transferred into another tube. A 10-μL aliquot of this aqueous layer was mixed with 90 μL of ultrapure water and 500 μL of chilled ethanol (100%, Sigma-Aldrich, St. Louis, MO, USA), chilled at −20 °C for 60 min, and then centrifugated at 4,000 × g at 4 °C for 10 min. Supernatants, which contained the peptides, were transferred using a pipette into a new tube and dried by vacuum centrifugation at max temperature 37 °C in “HPLC” setting (Genevac SP Scientific, Warminster, PA, USA). After vacuum centrifugation, the dried samples were dissolved in 100 μL of 50 mM ammonium bicarbonate (Thermo Fisher Scientific, Waltham, MA, USA), 2 μL of 550 mM dithiothreitol (Promega, Madison, WI, USA) was added, and samples were incubated at 50 °C for 50 min to reduce disulfide bonds. After incubation, 4 μL of 450 mM iodoacetamide (Sigma-Aldrich) was added, and samples were incubated in the dark at room temperature for 60 min to alkylate thiol groups. For enrichment and purification of peptides, C18 96-well plates (Glygen, Columbia, MD, USA) were washed and reconditioned with 600 μL of ultrapure water and then 600 μL of 80% acetonitrile (ACN, Thermo Fisher Scientific), 0.1% trifluoroacetic acid (TFA, Sigma-Aldrich) in water prior to loading samples. After sample loading, the 96-well plates were washed with 600 μL of ultrapure water to remove salts and other potential interfering substances. A 600 μL aliquot of 80% ACN, 0.1% TFA was added to elute the peptides. C18 96-well plates were centrifuged at 4 °C for 1 min at 1,000 × g in each step, and each eluate was dried by vacuum centrifugation.

### Peptide analysis via liquid chromatography–tandem mass spectrometry (LC–MS/MS*)*

Peptides were analyzed using an Orbitrap Fusion™ Lumos™ Tribrid™ mass spectrometer (Thermo Scientific, Waltham, MA) combined with a Waters nanoAcquity Ultra-Performance Liquid Chromatography (UPLC) (Waters, Milford, MA). Each dried sample was reconstituted with 50 μL of 3% ACN with 0.1% formic acid. One microliter of each sample was enriched and desalted with a C18 180 μm × 20 mm, 5-μm bead nanoAcquity UPLC trap column (Waters), and separated with a 100 μm × 100 mm, 1.7-μm bead Acquity UPLC Peptide BEH C18 column (Waters). Peptides were separated over 60 min at a flowrate of 0.5 μL/min with a gradient using the following proportions of mobile phases A (0.1% formic acid in water) and B (0.1% formic acid in ACN) and time points: 3–11.5% B, 0–10 min; 11.5–20% B, 10–31 min; 20–30% B, 31–36 min; 30–95% B, 36–45 min; 95% B, 45–54.5 min, 95–3% B over 30 s, and then finally the column was equilibrated with 97% A for 5 min. Peptides were ionized with an electrospray voltage of 2,320 V and an ion transfer tube temperature of 300 °C. In full MS scans, MS spectra were acquired in positive ionization mode over an m/z (mass-to-charge) range of 300–2000 by the Orbitrap at resolution of 60,000. The MS cycle time was set to 3 s. Following an MS scan, precursor compounds were automatically selected for MS/MS analysis by the acquisition software based on the following criteria: ion-intensity threshold 5.0 × 104, charge state 2–8 and exclusion time of 60 s. The fragmentation mode was set to higher-energy collisional dissociation (HCD) with normalized collision energy 30% for all precursor ions. MS/MS spectra were acquired in the positive ionization mode by the Orbitrap at resolution of 60,000. To minimize sample carry over, we ran samples from each study arm together (“Bovine” or “Soy”) and “BovE”/“SoyE” samples were run first. Furthermore, we performed blank runs between each sample and two blank runs between “bovine” and “soy” samples.

### Data analysis

Peptide identification from raw files was conducted using ProteomeDiscoverer (v.2.4; Thermo Fisher Scientific) based on database searching using both in-house HM protein sequences (*n* = 382) and in-house BM protein sequences (*n* = 490) for samples collected during the BM phase, and both HM protein sequences and soybean protein sequences [*n* = 757, *Glycine max* (soybean) in UniProtKB] for samples collected during the SB phase. Cleavage sites (cleavage enzyme) were set to “non-specific.” Precursor mass tolerance was set to 10 ppm with fragment mass tolerance of 0.1 Da. Minimum and maximum lengths of peptide were set to 4 and 160, respectively. Potential modifications included phosphorylation (+79.966 Da) at serine and threonine, oxidation (+15.995 Da) at methionine and acetylation (+42.011 Da) at peptide N-terminal. Carbamidomethylation (+57.021 Da) of cysteine was specified as a fixed modification. The abundances of each identified peptide were measured using the area under the curve of the extracted ion chromatograms based on ion intensity. Raw abundance of peptides with the same amino acid chain but different modifications were mathematically combined. Only proteins with >1 unique peptide identified were reported for further data processing.

The mass spectrometry proteomics data have been deposited to the ProteomeXchange Consortium^1^ via the PRIDE partner repository (20) with the dataset identifier PXD067746.

### Additional peptidomic analysis of soy beverage samples

Additional peptidomic analysis was also performed using the SB intervention (Pacific Foods Ultra Soy Original, Tualatin, OR). One sample of the same SB used in the study was analyzed in duplicate in a separate analysis from HM samples. Sample preparation, peptide extraction, and data analysis were similar to the previously described methods used for HM samples with the following exceptions: (1) initial aliquot volumes used for peptide extraction were 500 μL of SB, (2) 100 μL of sample supernatant was mixed with 500 μL of chilled ethanol during the ethanol precipitation step and no ultrapure water was used, and (3) C18 cartridges (5-mL tube volume, 500 mg bed weight, 45 μm particle size, Sigma-Aldrich) were used during solid phase extraction instead of C18 96-well plates.

### Dietary assessment

To assess typical dietary intakes, participants completed two 24-h dietary recalls via the Automated Self-Administered 24-h (ASA24; version 2020) Dietary Assessment Tool developed by the National Institute of Health National Cancer Institute (Bethesda, MD). The ASA24 is a public-access, web-based tool developed for researchers, clinicians, and educators and is based on the validated United States Department of Agriculture “automated multiple-pass method” (22). Participants completed the 24-h diet recalls within 2 wk. prior to the baseline HM collection (d0) on two nonconsecutive days, including one weekday and one weekend day. One of the participants was unable to complete a 24-h diet recall on a weekend day and therefore completed both recalls on two weekdays. The two dietary recalls were averaged for each individual and means were used for additional analysis and reported in results.

### Collection of additional data

Information related to participant and infant health, food allergy and intolerance history, reproductive history, anthropometric and demographic data was collected via telephone during the week prior to the baseline collection period (d0).

### Statistical methods

Raw abundances of individual peptides were transformed into relative abundance by taking the individual abundance values for each peptide and dividing them by the total abundance of all identified peptides. In the bovine phase, this total abundance included identified human- and bovine-derived peptides and, in the soy phase, the total included identified human- and soy-derived peptides. Statistical analysis of the peptide and protein relative abundance (range of 0–1) data was performed using R [v4.4.3, (21)]. A zero-inflated generalized linear mixed model with beta family was used to evaluate the abundance of peptides across time points of sample collection (BovE, Bov2Hr, Bov4Hr). Peptides that were detected in less than 15% of the samples (*n* = 44) were not included in this analysis. A glmmTMB (23) package in R was used to fit a model with time point as a fixed effect and categorical variable, participant identification code as a random effect, and an autoregressive [AR (1)] variance–covariance structure to account for the repeated measures effect. Residuals of the model were tested for normality and homogeneity of variance via the DHARMa package [v0.4.6, (24)]. The estimated marginal means for each time point were estimated using the emmeans package and pairwise comparison among groups was analyzed using pairs() function from the emmeans package [v1.10.3, (25)]. Probability (p) values were adjusted for multiple comparisons using a Bonferroni correction. Significance for the specified responses was declared at *p* ≤ 0.05.

When peptide abundance was highly zero-inflated and had a skewed distribution which resulted in violation of assumptions of generalized linear mixed effects models, a non-parametric Kruskal–Wallis test was used to evaluate the time point effect. All protein variables (*n* = 6) and some peptide variables (with zero inflation and skewed distribution, *n* = 6) were analyzed using kruskal.test function from the stats R package [v4.4.1, (21)] to test for significant differences in peptide and protein abundances among time points.

The nonmetric dimensional scaling (NMDS) ordination analysis of overall bovine-derived peptide communities was performed using the Bray–Curtis dissimilarity matrix. The vegan [v2.6.6.1, (26)] package was used to estimate the Bray-Curtis distance matrix. A Permutational Multivariate Analysis of Variance with 999 permutations was performed using the adonis2() function in the vegan package to quantify the effect size of variables explaining Bray-Curtis distance. The phyloseq [v1.48.0, (27)] package in R was to visualize the differences in ordinations among time points.

The estimated means and standard error of peptide variables from the generalized linear mixed model were reported as relative abundances on a percentage scale of 0–100%. The means and standard deviations for bovine proteins along with *p* values from Kruskal Wallis tests were reported in Supplementary Table 4. For peptides which were detected in <15% of all samples, average relative abundance and raw abundance of a given peptide can be found in Supplementary Table 1.

*T*-test, Wilcoxon test, and Fisher’s Exact test were used to compare demographic and dietary intake variables between the full participant group and peptidomics subset. Unless otherwise noted, data provided in this manuscript represent mean ± SD.

## Results

### Participant characteristics and sample information

**Figure 2:**
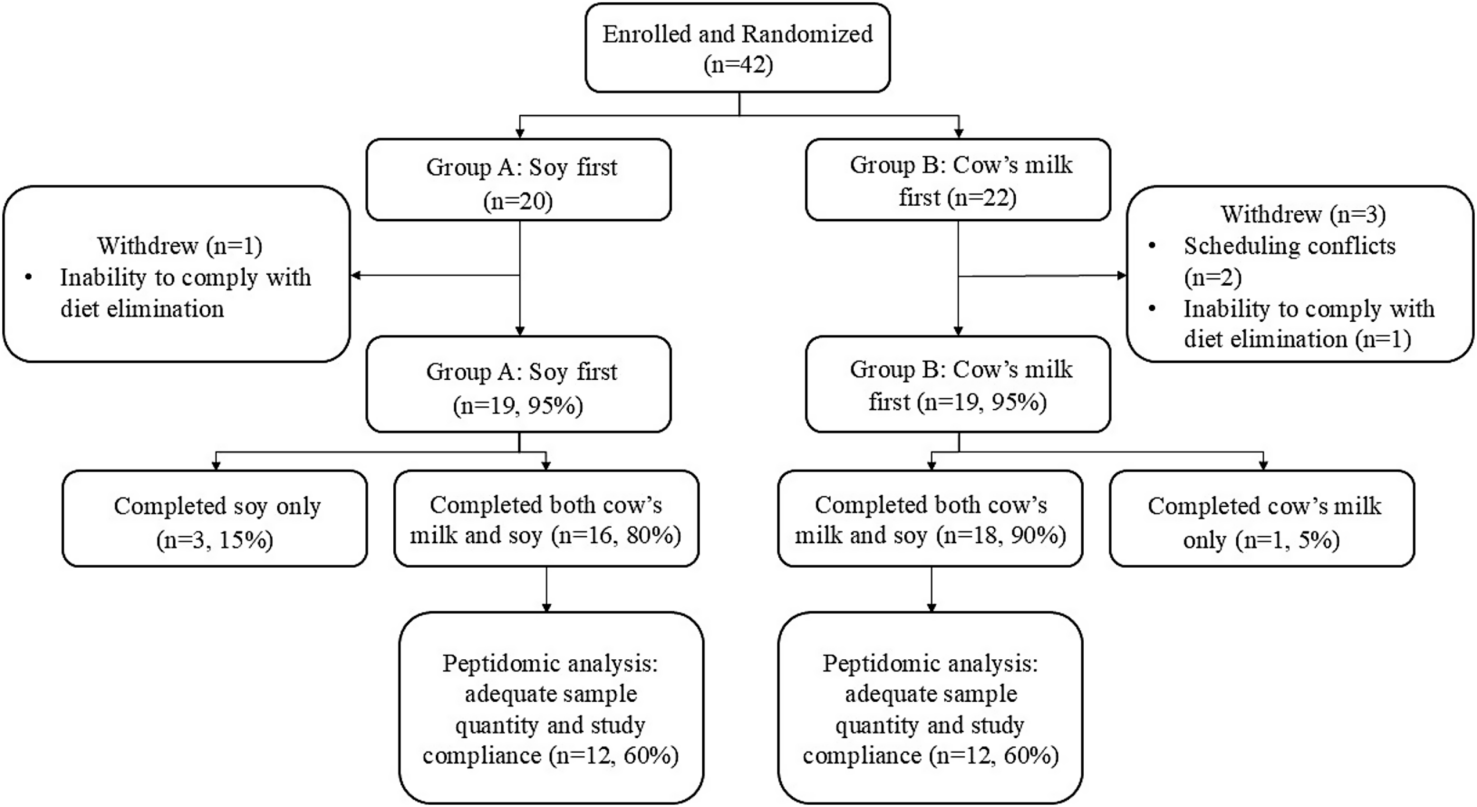
Flow chart describing participant enrollment, randomization and withdrawal in the parent study and inclusion for the peptidomics subset analyzed and described herein.

In total, 42 women enrolled in the study: 34 completed both phases of the study, four completed only one phase of the study, and four withdrew due to scheduling conflicts or inability to comply with the diet elimination or intervention (Figure 2). A subset of 24 participants had sufficient quantities of HM and adequate daily compliance with dietary elimination and study protocols and were therefore used for peptidomics analysis. In the peptidomics subset, BovE and SoyE (d6 and d16; hr. 0) HM samples were collected within a range of times (0520–1700 h) with the majority (>91%) collected in the morning. On the third day of each dietary intervention (d8 and d18), Bov2Hr and Soy2Hr HM samples were collected 0800–1900 h with >54% collected in the morning, and Bov4Hr and Soy4Hr HM samples were collected 1,014–2,100 h with >81% collected after noon.

Health and demographic information for women and their infants in the subset described herein (*n* = 24) are provided in Table 1. Overall, this subset of women (29.8 ± 4.5 y of age) was predominantly non-Hispanic white (92%), had completed some college (100%), had one child (54%), and did not report food-related reactivity (83%) or diagnosed food allergy (100%). No differences between the full group of all participants and the subset described herein were detected when comparing demographic variables using Fisher’s Exact, Welch’s t-test, and Wilcoxon tests as appropriate based on distributions of normality. No differences were found for selected nutrient and food group intakes when comparing the peptidomics subset to the group of all participants (Table 2). Participants reported intakes equal to or greater than Dietary Reference Intake Recommended Dietary Allowance values for most nutrients, except the average consumption of vitamins A, C, D, and E, and folate (Table 2) (28–31). Mean intakes of vitamins A, C, D, and E were also below the Estimated Average Requirement (EAR) values, while mean consumption of folate was above the EAR value. It is noteworthy that these intakes do not reflect additional supplementation, such as from multivitamins. In the subset discussed herein, 58% (*n* = 14) of participants reported regularly consuming a prenatal multivitamin, 33% reported not regularly consuming a multivitamin, and 8% did not provide information on multivitamin use.

**Table 1:** Selected health and demographic information of women and infants participating in the parent study (*n* = 38) and the 24 women whose milk samples were analyzed for this subset analysis.

	All participants (*n* = 38)	Subset (*n* = 24)
Maternal		
Age, y		
Mean ± Standard Deviation	30.2 ± 4.6	29.8 ± 4.5
Range	21–38	21–38
Race, *n* (%)		
White, non-Hispanic	36 (95)	22 (92)
White, Hispanic	1 (3)	1 (4)
Asian	1 (3)	1 (4)
Highest educational attainment, *n* (%)		
Completed high school	1 (3)	0 (0)
Some college	6 (16)	4 (17)
Bachelor’s degree	23 (61)	15 (62)
Graduate/Professional degree	8 (21)	5 (21)
Household income, *n* (%)		
Declined to answer	1 (3)	0 (0)
<$20,000	3 (8)	2 (8)
$20,000–$35,000	3 (8)	2 (8)
$35,000–$50,000	6 (16)	5 (21)
$50,000–$75,000	10 (26)	7 (29)
$75,000–$100,000	7 (18)	3 (13)
>$100,000	8 (21)	5 (21)
Parity, *n* (%)		
1	19 (50)	13 (54)
2	11 (29)	8 (33)
3+	8 (21)	3 (13)
Current or history of food allergy, *n* (%)		
Yes	4 (11)	0 (0)
No	34 (89)	24 (100)
Food-related reactivity, *n* (%)		
Yes	8 (21)	4 (17)
No	30 (79)	20 (83)
Infant		
Age, wk		
Mean ±Standard Deviation	26.8 ±15.6	26.5 ± 14.2
Range	2.0–53.7	4.0–50.3
Sex, *n* (%)		
Male	24 (63)	14 (58)
Female	14 (37)	10 (42)

No differences were detected when comparing demographic variables across groups using Fisher’s Exact, Welch’s *t*-test, and Wilcoxon tests. Not all percentages within a parameter add to 100% due to rounding.

**Table 2:** Daily consumption of energy, selected nutrients, and relevant food groups by all 38 women participating in the parent study and the 24 women whose milk samples were analyzed for this subset.

	All participants (*n* = 38)	Subset (*n* = 24)	RDA/AI*
	Mean ± SD	Mean ± SD	
Energy and macronutrients			
Energy, kcal	2448.4 ± 811.1	2356.6 ± 572.4	ND
Protein, g	93.8 ± 32.5	89.0 ± 26.9	71
Total Fat, g	112.8 ± 39.4	108.0 ± 32.3	ND
Carbohydrates, g	271.2 ± 112.0	262.4 ± 79.0	210
Micronutrients			
Calcium, mg	1187.1 ± 623.2	1177.8 ± 527.9	1,000
Iron, mg	16.3 ± 6.2	15.3 ± 3.6	9
Magnesium, mg	374.8 ± 137.5	354.2 ± 96.4	310–320
Phosphorus, mg	1635.8 ± 633.1	1566.7 ± 489.5	700
Potassium, mg	2895.8 ± 952.7	2827.3 ± 825.8	2800*
Sodium, mg	3944.6 ± 1213.2	3922.2 ± 1000.9	1500*
Zinc, mg	12.9 ± 4.5	11.9 ± 3.2	12
Copper, mg	1.7 ± 0.6	1.7 ± 0.5	1.3
Selenium, μg	134.9 ± 50.2	131.8 ± 36.4	70
Vitamin C, mg	72.2 ± 53.5	71.3 ± 47.3	120
Thiamin, mg	1.9 ± 0.9	2.0 ± 0.8	1.4
Riboflavin, mg	2.5 ± 1.0	2.5 ± 1.0	1.6
Niacin, mg	27.1 ± 9.5	26.8 ± 8.8	17
Vitamin B-6, mg	2.1 ± 0.8	2.1 ± 0.8	2.0
Folate, μg	465.2 ± 184.5	446.0 ± 113.6	500
Vitamin B-12, μg	5.1 ± 2.1	5.2 ± 2.1	2.8
Vitamin A, μg RAE	723.0 ± 354.0	702.6 ± 271.9	1,300
Vitamin E, mg *α*-tocopherol	11.9 ± 8.5	10.0 ± 2.6	19
Vitamin K, μg phylloquinone	166.0 ± 137.3	128.1 ± 71.6	90*
Vitamin D (D2 + D3), μg	5.1 ± 5.1	6.1 ± 6.1	15
Selected food groups			
^1^Soy products, oz	0.3 ± 0.7	0.4 ± 0.8	ND
^2^Total dairy, cup	2.2 ± 1.9	2.2 ± 1.8	ND
^3^Milk products, cup	0.7 ± 0.9	0.8 ± 1.1	ND
Yogurt, cup	0.1 ± 0.3	0.2 ± 0.3	ND
Cheese, cup	1.4 ± 1.3	1.3 ± 0.9	ND

No differences were found between groups based on two-sample t-tests and Wilcoxon tests. Reported dietary intakes do not include nutrient intake from supplements. In the subset discussed herein, 58% (*n* = 14) of lactating participants reported regularly consuming a prenatal multivitamin. DRI values for selected nutrients are provided based on life-stage group of 19–30 y and 31–50 y females during lactation. A range is provided if values for age groups were different. An asterisk (*) delineates AI from RDA values.

1 Excludes fortified soy milk and immature soybeans.

2 Includes milk, yogurt, cheese, and whey.

3 Includes fluid milk, buttermilk, evaporated milk, dry milk, and calcium fortified soy milk.

AI, Adequate Intakes; DRI, Dietary Reference Intakes; ND, not determined; RDA, Recommended Dietary Allowances; SD, Standard Deviation.

### Peptidomic analysis of human milk samples collected during bovine milk phase

A total of 2,964 unique peptides were identified in the 72 HM samples collected during the BM phase. Of these, 2,704 peptides were identified as being of human origin, corresponding to 73 HM-derived parent proteins, and 121 peptides were of bovine origin, matched to 9 bovine-derived parent proteins (Supplementary Tables 1, 2). A total of 139 peptides could not be distinguished between human and bovine origin. Given that the aim of this research was to identify peptides unique to BM, peptides that shared homology and could not be distinguished between bovine or human origin were deemed irrelevant to additional analysis. Of the 9 identified bovine parent proteins, 3 were identified using only 1 unique bovine peptide and were therefore removed before additional analysis. Of the remaining 6 bovine proteins, β-lactoglobulin represented the highest mean raw and relative abundance, followed by *κ*-casein, *α*s1-casein, β-casein, α-lactalbumin protein variant D, glycosylation-dependent cell adhesion molecule 1, and αs2-casein (Table 3).

**Table 3:** Number and mean relative abundance (%; range 0–100) of identified bovine-milk-derived peptides aggregated by parent protein in human milk samples (*n* = 72) collected before and after bovine milk consumption.

Parent protein	Number of peptides	Relative abundance (%)
β-Lactoglobulin	64	1.061
κ-Casein	10	0.136
α_S1_-Casein	19	0.027
β-Casein	14	0.024
α-Lactalbumin protein variant D	3	0.015
Glycosylation-dependent cell adhesion molecule 1	8	0.004

**Figure 3:**
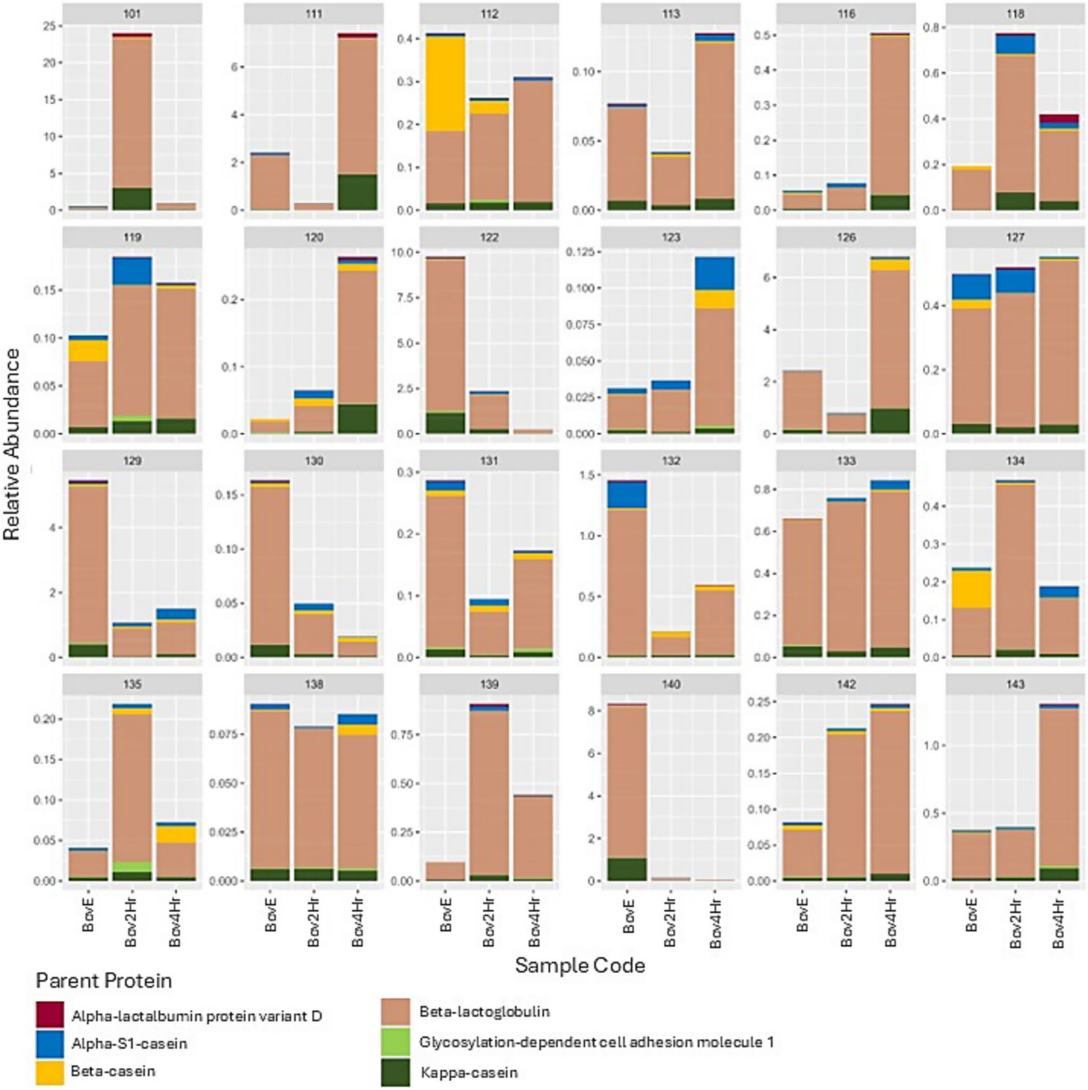
Relative abundance (range: 0–100%) of bovine milk peptides (characterized by bovine parent protein) by participant and sample. BovE represents d6/d16 hr. 0 collection after 5 days of dietary bovine milk elimination. Bov2Hr and Bov4Hr represent d8/d18 hr. 2 and 4, respectively, after maternal consumption of 415 mL bovine milk during the reintroduction phase.

**Figure 4:**
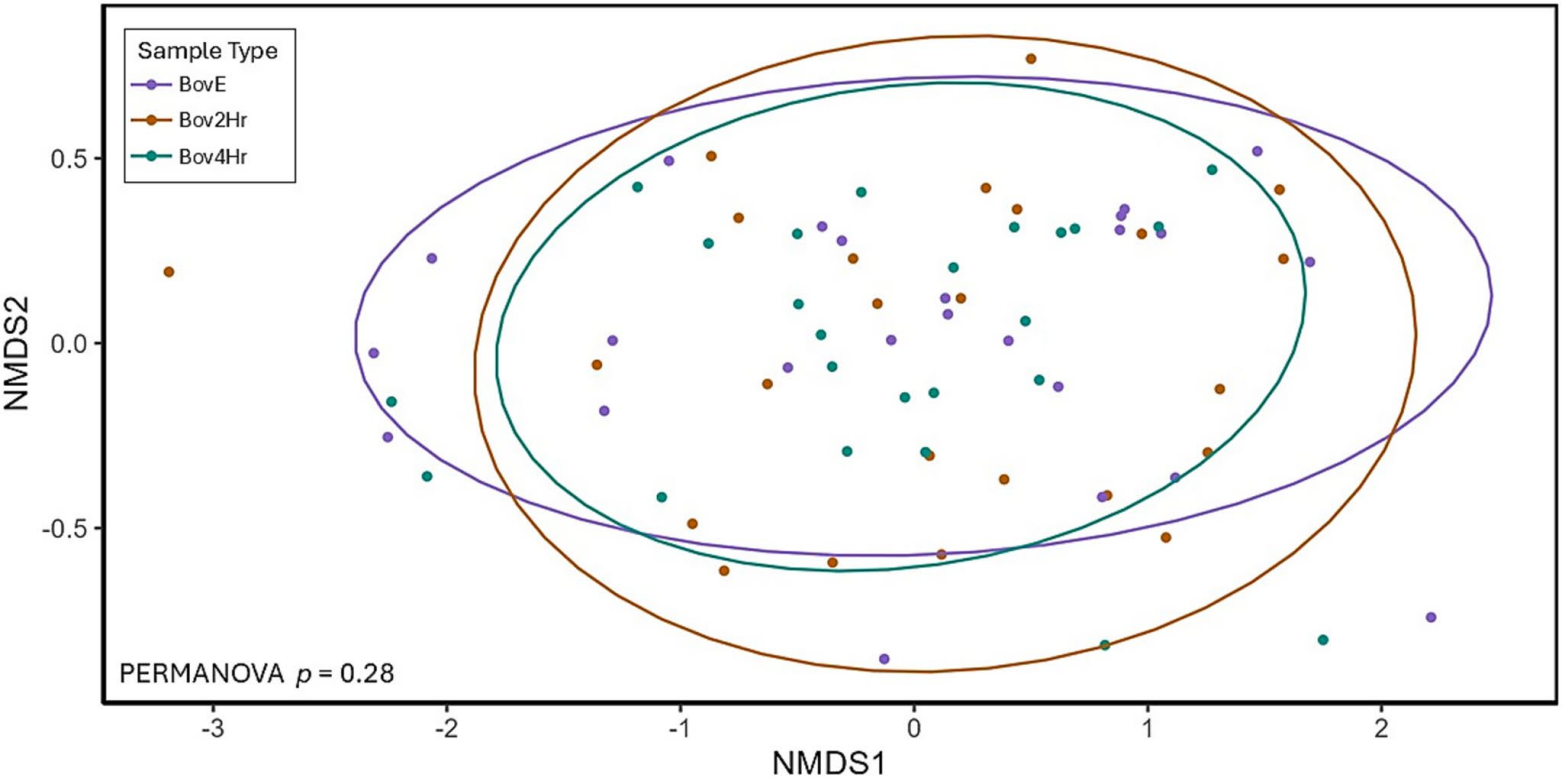
Non-metric multidimensional scaling (NMDS) plot showing Bray-Curtis distances and Permutational Multivariate Analysis of Variance (PERMANOVA) test comparing human milk peptidomic profiles organized by collection/time point. Ellipses cover 90% confidence level categorized by sample type. BovE represents d6/d16 hr. 0 collection after 5 days of dietary bovine milk elimination. Bov2Hr and Bov4Hr represents d8/d18 hr. 2 and 4, respectively, after maternal consumption of 415 mL bovine milk during the reintroduction period.

Relative abundance and timing of the appearance of bovine peptides after BM elimination and reintroduction were variable among participants (Figure 3). Results demonstrated significant differences (*p* ≤ 0.05) in relative abundance for 14 individual peptides when compared across the three collection time points (BovE, Bov2Hr, Bov4Hr) (Table 4). The majority (*n* = 9) of these significantly different peptides were associated with β-lactoglobulin. No differences in relative abundances were observed when peptides were aggregated at the parent protein level (Supplementary Table 3). Furthermore, no differences in relative abundances across the three collection time points were observed for the six bovine-derived peptides evaluated using Kruskal Wallis test (*p* > 0.05) (Supplementary Table 4). There was also no effect of time point on HM peptidomic profiles based on PERMANOVA analysis (*p* = 0.28) (Figure 4).

**Table 4:** Relative abundances (estimated means ± standard error of the mean) by sample type of significant identified bovine-milk-derived peptides in generalized linear mixed models.

Parent protein	Peptide sequence	BovE	Bov2Hr	Bov4Hr	*p* value
β-Lactoglobulin	VLVLDTDYKKY	1.92E-03 ± 6.07E-04^a^	5.78E-04 ± 1.85E-04^b^	1.21E-03 ± 4.10E-04^a^	<0.001
β-Lactoglobulin	KIIAEKTKIPAVF	2.86E-02 ± 1.12E-02^a^	6.61E-03 ± 1.63E-03^b^	3.74E-03 ± 8.76E-04^c^	<0.001
β-Lactoglobulin	YVEELKPTPEGDLEIL	2.44E-02 ± 7.30E-03^a^	4.09E-03 ± 1.85E-03^b^	6.31E-03 ± 3.45E-03^b^	0.006
β-Casein	NIPPLTQTPVVVPPF	1.37E-02 ± 5.03E-03^a^	1.48E-02 ± 5.52E-03^a^	5.56E-03 ± 2.16E-03^b^	0.006
β-Lactoglobulin	ALNENKVLVLDTDYKKY	9.87E-03 ± 4.60E-03^a^	1.58E-03 ± 4.82E-04^b^	2.05E-03 ± 6.69E-04^b^	0.006
β-Lactoglobulin	IDALNENKVLVLDTDYKKY	3.59E-03 ± 1.48E-03^a^	1.11E-03 ± 4.21E-04^b^	2.84E-03 ± 1.93E-03^ab^	0.006
κ-Casein	PPKKNQDKTEIPTINTIA	2.84E-02 ± 1.42E-02^a^	3.15E-03 ± 9.44E-04^b^	4.16E-03 ± 1.31E-03^b^	0.006
β-Lactoglobulin	VRTPEVDDEALEK	5.43E-02 ± 2.05E-02^a^	1.29E-02 ± 3.59E-03^b^	1.43E-02 ± 3.93E-03^b^	0.006
β-Lactoglobulin	TPEVDDEALEKF	5.44E-03 ± 1.63E-03^a^	9.39E-02 ± 6.52E-02^b^	8.98E-03 ± 3.61E-03^a^	0.007
Glycosylation-dependent cell adhesion molecule 1	AQPTDASAQFIRN	3.77E-03 ± 1.38E-03^a^	7.05E-04 ± 2.10E-04^b^	1.88E-03 ± 9.67E-04^ab^	0.011
α-Lactalbumin protein variant D	LDKVGIN	5.56E-03 ± 1.75E-03^a^	3.66E-02 ± 2.02E-02^b^	1.85E-02 ± 8.49E-03^b^	0.028
β-Lactoglobulin	VRTPEVDDEALEKFDKA	1.04E-02 ± 3.63E-03^a^	3.23E-03 ± 7.72E-04^b^	4.09E-03 ± 1.16E-03^b^	0.028
β-Lactoglobulin	KPTPEGDLEILLQ	9.67E-03 ± 2.86E-03^a^	2.91E-03 ± 1.28E-03^b^	3.97E-03 ± 1.53E-03^b^	0.036
CDC42 effector protein 3	PLLSPVTFSSKQ	2.84E-02 ± 8.96E-02^a^	5.70E-02 ± 1.80E-02^b^	4.63E-02 ± 1.46E-02^ab^	0.049

Means and standard errors are reported in relative abundance (%; range 0–100). Values within a row not sharing a common superscript differ based on pairwise comparison among groups (*p* ≤ 0.05). Due to the nature of pairwise testing, directional trends (e.g., increasing or decreasing over time) cannot always be inferred across all time points. This table should be interpreted accordingly. BovE represents d6/d16 hr 0 collection after 5 days of dietary bovine milk elimination. Bov2Hr and Bov4Hr represent d8/d18 hr 2 and 4 (respectively) after maternal consumption of 415 mL bovine milk during the reintroduction phase.

### Peptidomic analysis of human milk samples collected during soy beverage phase

We identified 2,287 unique peptides in the 72 HM samples collected during the SB phase. Of these, 2,271 peptides were of human origin, matched to 71 HM-derived parent proteins, and 8 peptides were of possible soy origin (Table 5), matched to 8 soy-derived parent proteins. Eight peptides could not be distinguished between human or soy origin. All 8 proteins of possible soy origin were matched using only 1 unique peptide; as such, we had low confidence in their identification as proteins, and they were not further analyzed or reported at a parent protein level. We next entered the eight peptides identified as soy-derived in the NCBI protein BLAST search engine using the blastp algorithm.^2^ Six of the eight identified peptides were matched with 100% identity to multiple nonhuman proteins from several nonhuman organisms, bacteria, or other potential food sources (including potato, peas, and other plants). Two (EKLFGLVRDSAGQLKASGTVVIDAAL and NLSELTLSLTNNIVCRIAL) of the eight identified peptides were matched with 100% identity to soy-derived proteins only (glycine max or glycine soja) although also shared >80% identity with other plants, foods, and nonhuman sources (such as a variety of beans and peanuts). These results introduce the possibility that these peptides could be derived from other foods present in the participants diets.

**Table 5:** Mean relative abundances (%; range 0–100) and prevalence by time point of peptides identified as potentially soy-derived in human milk samples collected after dietary elimination of soy and after 2 and 4 h of soy beverage consumption.

Peptide sequence	Number of samples SoyE (*n*, %)	Number of samples Soy2Hr (*n*, %)	Number of samples Soy4Hr (*n*, %)	Relative abundance (%)
EKLFGLVRDSAGQLKASGTVVIDAAL	12 (50)	14 (58)	14 (50)	0.108
THPKISDAAVVPMKDEAAGEVPVA	24 (100)	24 (100)	24 (100)	0.073
LEAAFGRFGFETIIVTGLP	24 (100)	24 (100)	23 (96)	0.055
VGGAALPDTAEKITFDSKLVAGPNGGSAGKLTVK	9 (38)	5 (21)	5 (21)	0.006
VVSKATSAEINQKASPGVPL	19 (79)	19 (79)	19 (79)	0.007
RSLVLLYNSSRLFGGGTLINKE	6 (25)	10 (42)	13 (54)	0.002
ADLDATILDIRPSETEAVAI	6 (25)	7 (29)	8 (33)	<0.001
NLSELTLSLTNNIVCRIAL	1 (3)	2 (8)	1 (3)	<0.001

SoyE represents d6/d16 hr 0 collection after 5 days of dietary soy elimination. Soy2Hr and Soy4Hr represent d8/d18 hr 2 and 4 (respectively) after maternal consumption of 415 mL soy beverage during the reintroduction phase.

### Peptidomic analysis of soy beverage samples

We identified 710 soy-derived peptides in the SB sample (*n* = 1) (Supplementary Table 5). None of the 8 soy-derived peptides identified in the HM samples were matched with 100% sequence homology to peptides identified in the SB.

## Discussion

BM-derived peptides were identified in HM collected after dietary elimination as well as 2 and 4 h after BM consumption. Relative abundances of BM-derived peptides were low, and there was interindividual variation in their appearance and abundance. Only 14 BM-derived peptides differed in relative abundance when comparing samples collected after BM elimination and those 2 and 4 h after BM consumption. Among these 14 peptides, abundances were not consistently higher in samples collected 2 or 4 h after BM consumption. In fact, BM-derived peptides were identified in all samples collected after 5 d of dietary elimination. Finally, although 8 peptides of possible soy origin were identified in human milk samples, they were unable to be matched to soy parent proteins with adequate confidence and shared a high level of homology with other foods and organisms.

Results from this study support previous research demonstrating the presence of bovine-derived peptides in HM following maternal consumption of BM. We identified a total of 121 unique bovine-derived peptides in 72 HM samples collected from women after a 5-d dietary BM elimination and 2 and 4 h after BM consumption (415 mL). While several studies have reported the presence of BM proteins in HM using immunochemical detection methods (7, 32, 33), few have specifically investigated BM-derived peptides in HM after BM consumption using more sensitive techniques such as LC–MS/MS. In 6 HM samples collected by women 2 h after consuming 200 mL of BM, Picariello et al. (8) identified a total of 11 BM-derived peptides. The two peptides they identified that were derived from β-lactoglobulin (YVEELKPTPEGDLEIL and YVEELKPTPEGDL) were also detected in our samples, and both studies identified peptides from *α*s1-casein. In contrast, peptides from osteopontin, toll-like receptor 9, bile salt-activated lipase, perilipin-2, xanthine dehydrogenase, and lactotransferrin were identified in their samples but not ours, and *κ*-casein, β-casein, α-lactalbumin, and glycosylation-dependent cell adhesion molecule 1 were detected in our samples but not theirs.

Several BM-derived peptides reported by Dekker et al. (34) were also identified in our HM samples, including the most abundant peptides derived from β-lactoglobulin (TPEVDDEALEK) and αs1-casein (HIQKEDVPSER). Dekker et al. (34) also identified peptides from several other BM proteins, including desmoplakin, ninein, fatty acid synthase, mucin-16, serum albumin, lactotransferrin, osteoponin, cathepsin D, mosein, and 14 others that were not identified in our samples. While Dekker et al. (34) reported peptides from a broader range of dietary sources and allergens, our analysis was intentionally limited to soy- and bovine milk-derived peptides, which aligned with the allergens targeted in the maternal dietary intervention. Although we did not aim to replicate their broader peptide profiling approach, our findings still allow for meaningful comparisons specific to bovine-derived peptides. Future studies incorporating a wider range of dietary proteins and allergens may provide further insights into the diversity and dynamics of food-derived peptides in HM.

Some of the variability in identified peptides across different studies is expected given the inter- and intra-individual variability of bovine-derived peptide appearance as seen in previous research. This variability may also be related to inconsistency in methodologies, such as the use of different search engines and databases, or, in the former study (8), the addition of a serine-protease inhibitor to HM samples prior to freezing. Additionally, participants in the study by Dekker et al. (34) did not eliminate or consume BM as part of HM collection, which may best explain some of the variation in BM-derived peptides detected in HM samples between our studies. More research is needed to fully understand and explain these differences in results and the myriad factors that impact non-human peptide appearance.

Finally, the predominance of β-lactoglobulin-derived peptides observed in our analysis, as well as in previous studies, may partly reflect the absence of an equivalent protein in human milk. In contrast, peptides derived from other bovine milk proteins, such as caseins, may be underrepresented because many sequences are shared or highly similar between bovine and endogenous human caseins, and were therefore excluded from statistical analysis due to their ambiguous origin.

Though we hypothesized that the relative abundance of these bovine peptides would be greatest in HM collected 4 h after maternal consumption of BM, our results do not support this hypothesis. In the 78 bovine-derived peptides found in at least 15% of HM samples, only 14 were different across the three collections (BovE, Bov2Hr, and Bov4Hr). Surprisingly, for many of these peptides, relative abundances were higher in HM collected after 5 days of dietary elimination. Given that we were unable to quantify the specific amounts of these 14 peptides, and that relative abundances were small (ranging from 0.0005–0.09%), it is difficult to assess the clinical relevance of these differences based on collection time in a food-allergic population. Although quantities of the peptides identified in our study were not evaluated, the very low abundances found in the fourteen variable peptides above are likely below thresholds needed to induce an allergic reaction in most infants. In fact, an analysis by Munblit et al. (35) estimates that the β-lactoglobulin content in human milk after maternal bovine milk consumption is insufficient to trigger allergic reactions in over 99% of infants with IgE-mediated CMA based on current threshold data. Gamirova et al. (36) further estimated the probability of a single breastfeed containing levels of β-lactoglobulin needed to provoke a reaction in an allergic infant at 1:2893. While these estimates do not account for non-IgE-mediated mechanisms or other potential bovine milk-derived allergens, they support our hypothesis that the peptides detected in our results are also very likely below thresholds needed to provoke an allergic reaction. Additionally, given that the measurement of relative abundances is inherently dependent on the abundance of all other peptides detected within a sample, it is possible that the observed increases or decreases in specific BM-derived peptides after the dietary elimination and BM consumption reflect shifts in the overall peptide profile, rather than true changes in their absolute concentration. Future research should aim to incorporate quantitative techniques to assess peptide transfer in human milk following maternal dietary consumption.

While we were able to detect variation across timepoints in several bovine-derived peptides, we did not observe any differences at the parent protein level. We suspect that aggregating peptides at the protein level may mask the significant differences identified at the individual peptide level. These results may suggest that the presence and transfer of dietary peptides in human milk is peptide-specific rather than protein-wide.

Unexpectedly, results from this study suggest the presence of bovine-derived peptides in HM collected after 5 d of maternal BM elimination. These results do not support our hypothesis that peptides from BM would not be detectable via LC–MS/MS in HM produced after a 5-d elimination period. In the previously mentioned study published by Picariello et al. (8) using similar methodologies, 6 women were also assigned to a control group and HM samples were collected after women were asked to follow a milk- and dairy product-free diet for at least 1 week. Interestingly, 4 bovine-derived peptides were identified in these control samples. It is possible that 5 days of dietary elimination is not sufficient to eliminate the presence of bovine peptides in human milk.

Other studies evaluating the timing of appearance and disappearance of non-human peptides in HM produced after dietary elimination and utilizing the same LC–MS/MS methods are limited. In a second study, Picariello et al. (9) collected HM samples from one healthy, atopic participant at 1, 2, 3, 4, and 6 h after consumption of an unspecified amount of BM. In this time course, peptides matched to parent proteins β-lactoglobulin and β-casein peaked in abundance at 2 h and were no longer detected at 6 h. Unfortunately, this only reflects the time course of peptide disappearance after consumption for one individual and cannot be generalized to all nursing individuals or for other peptides. The timing of the disappearance of bovine peptides may be equally as variable on an individual level as the appearance has been demonstrated to be.

Understanding the timing of disappearance of potential dietary allergens in human milk requires more in-depth research into the kinetics of peptide transfer from the maternal diet. While most dietary proteins are broken down into small peptides and amino acids during digestion, some intact proteins or peptide fragments can survive this process and cross the intestinal barrier through various mechanisms (37), entering the bloodstream and subsequently the mammary gland. The timing and kinetics of this process, including both the appearance and clearance of peptides in human milk, are highly variable both within and between individuals as has been previously described (19). Although factors such as maternal atopy or long-term cow’s milk consumption may influence peptide presence in milk, the underlying mechanisms remain unclear and are not yet well defined (19, 34, 38). Further research is needed to evaluate the specific timing, half-lives, and clearance of dietary peptides from the maternal system.

The unexpected detection of bovine-derived peptides after a 5-d dietary elimination period may alternatively reflect several contributing factors. It is important to consider that HM for our study was collected in participant homes. Although participants were asked to eliminate BM-containing foods and ingredients from their diets, in many cases, other household members may have continued consuming bovine products in the home, increasing the potential for environmental exposure. Participants were asked to wash their hands and use gloves prior to milk collection, and aseptic techniques were used during laboratory analysis, but the possibility of environmental contamination remains. In addition, unintentional dietary exposure to trace amounts of bovine peptides in processed foods or cross-reactive sources may have occurred. The use of highly sensitive methods, such as LC–MS, in the detection of these bovine peptides could account for the identification of bovine peptides in very low abundances in all analyzed samples. Low relative abundances of these non-human peptides may be persistently present in human milk. Additional research is needed to determine whether such low relative abundances are sufficient to induce symptoms in infants with BM sensitivity. Although the infants in our study did not have a current or confirmed diagnosis of food allergy, with the exception of one infant with a possible history of cow’s milk allergy or sensitivity, future studies would benefit from including participants with symptomatic infants and integrating clinical symptomatology alongside molecular findings. This approach could provide critical insight into whether trace peptides can elicit a reaction in HM-fed infants, or whether other immunological or physiological mechanisms are involved. Overall, these results underscore the need for further investigation into the biological and technical factors that influence the presence and detection of food-derived peptides in HM.

Our results suggest that soy-derived peptides may not be present in HM produced both after maternal soy elimination and after consumption of SB – at least in this study population. Although 8 soy-derived peptides were initially identified in HM, several of these peptides were found to have 100% identity matched to other organisms and plant proteins based on BLASTp searching. The high level of shared homology of these peptides with other potential food sources, and the identification of only eight peptides, which could not be matched at a protein level with adequate confidence using Proteome Discoverer, leaves a level of uncertainty that these peptides are derived from the SB intervention in the study.

Furthermore, LC–MS/MS analysis of the SB provided in our study, did not detect any of the 8 peptides found in the HM that were putatively linked to soy. This finding raises further uncertainty that the “soy” peptides we identified were truly derived from the SB intervention. Alternatively, the absence of these peptides in the SB sample may reflect differences in proteolysis when comparing the sample preparation of the SB for LC–MS/MS analysis and the biological digestion occurring in the GI tract of a lactating individual. Overall, these results suggest that the 8 peptides were more likely derived from other dietary or environmental sources, or are analytical artifacts. These findings do not support our initial hypothesis that the relative abundance of soy-derived peptides would be highest in milk produced 4 h after SB consumption.

Research on the presence of soy proteins and peptides in HM remains limited. To our knowledge, published research on soy compounds in HM have solely documented the presence of isoflavones and no soy-derived proteins or peptides have yet been identified in HM (39–41). Zhu et al. (12) provides the most comprehensive research thus far describing the presence of nonhuman peptides identified in HM. In 6 HM samples collected from 6 Italian women at 5 to 7 wk. postpartum, none of the 109 identified nonhuman peptides were reported as being derived from soy. Notably, this study took a non-targeted approach to peptide and protein identification utilizing two database searches of LC–MS/MS data. First, the authors searched using a database of “*Homo sapiens* and allergens” and second, searching against a database of “*Homo sapiens* and organisms with identified allergens.” Dekker et al. (34) evaluated differences in the abundance of non-human peptides in human milk based on maternal allergy status using a customized database that included HM proteins, BM proteins, and allergen proteins. Similar to our findings, they did not report any soy-derived peptides or allergens across 20 human milk samples, though peptides from other dietary sources, such as wheat and corn, were identified. The methods used in these studies contrast with our direct approach of searching using a soybean-specific database and may explain some differences in soy peptide identification.

Additionally, we used an ultra-processed SB as our dietary intervention. The transfer of allergens from other differently processed soy-based foods may yield different results. Furthermore, no soy sensitivities or allergies were reported in our sample in mothers nor their infants. Future research should investigate the presence of soy-derived peptides after consumption of other soy foods and evaluate if transfer of these peptides may be more likely in a soy sensitive or allergic population.

### Limitations and strengths of study

Results of our study are not generalizable given the lack of diversity of the study sample population and the fact that they did not report being allergic (or having infants who were allergic) to BM or SB. Also, samples were collected during the first 2 years of the COVID-19 pandemic and thus were collected by participants in their homes rather than in a research setting. Though detailed written and verbal instructions were provided to each participant to limit the risk of contamination during sampling, contamination is still possible. Additionally, although participants were provided with a list, and discussed foods and ingredients containing BM and soy with a study dietitian, their diets were not strictly controlled and therefore consumption of these foods unintentionally is possible.

Furthermore, there are several limitations associated with the analysis of BM- and soy-derived peptides via LC–MS/MS. It is possible that technical artifacts may be present due to sample carryover from chromatography despite efforts to minimize this risk. While ethanol precipitation is widely used to effectively extract peptides from biological sources, we acknowledge that some larger polypeptides may have been excluded by this sample preparation method. As our study aimed to focus on smaller peptides likely to survive digestion and be transferred to HM, this approach was considered appropriate for our research objectives. Finally, we did not specifically account for the possibility that foreign peptides may be bound to carrier proteins when present in HM. This remains a potential limitation, as such associations may influence peptide recovery or detection.

Despite these limitations, our study offers several strengths. We employed a randomized dietary intervention design that incorporated both dietary elimination and reintroduction, alongside two distinct dietary interventions in a crossover format. Furthermore, in contrast to similar published studies and methods, our study was able to evaluate these outcomes using a comparatively larger sample size. Additionally, our study utilized LC–MS to identify the presence of BM- and soy-derived peptides. These rigorous and sensitive methods may enhance the detection of very low abundant peptides that otherwise have not been detected or documented in previous literature. Finally, our study benefits from being able to evaluate the impacts of time after BM/SB consumption on peptide appearance.

## Conclusions

BM-derived peptides are present in human milk although in low relative abundances overall. The relative abundance of some BM-derived peptides differed when comparing human milk samples collected after dietary elimination and those collected 2 and 4 h after BM consumption. These peptides were not found to be consistently greater after consumption and were identified at very low relative abundances that are likely below thresholds needed to induce an allergic reaction in most infants. The identification of BM-derived peptides in all samples collected after 5 days of dietary elimination may indicate that 5 days of elimination is not adequate for the elimination of BM-derived peptides in their entirety or may suggest that low levels of these non-human peptides are persistently present in human milk. Additional research utilizing LC–MS and other sensitive methodologies would be beneficial in evaluating the timing of non-human peptide disappearance or if these peptides are present despite dietary elimination as a result of other mechanisms of environmental exposure. No confident soy-derived peptides from the SB intervention were identified in HM after maternal consumption. More research is needed to investigate the presence of other non-human peptides, such as those from soy, in larger samples sizes, evaluate why the transfer of food-derived bovine allergens into HM is so variable, and elucidate whether this has any impact on infant health.

## Data Availability

The data presented in the study are deposited in the ProteomeXchange via PRIDE repository: https://www.ebi.ac.uk/pride/archive/projects/PXD067746.

## Footnotes

^1^ http://proteomecentral.proteomexchange.org

^2^ https://blast.ncbi.nlm.nih.gov/Blast.cgi?PROGRAM=blastp&PAGE_TYPE=BlastSearch&LINK_LOC=blasthome

## Supplementary material

SUPPLEMENTARY FILE 1 This document provides a copy of the Daily Dietary Survey which measured participant dietary compliance during the study.

## References

[1] Boyce JA Jones SM Rock L Sampson HA Cooper SF Boyce S. Guidelines for the diagnosis and management of food allergy in the United States: report of the NIAID-sponsored expert panel. J Allergy Clin Immunol. (2010) 126:S1–S58. doi: 10.1016/j.jaci.2010.10.007 21134576 PMC4241964

[2] Warren CM Otto AK Walkner MM Gupta RS. Quality of life among food allergic patients and their caregivers. Curr Allergy Asthma Rep. (2016) 16. doi: 10.1007/s11882-016-0614-9, PMID: 27048239

[3] Gupta RS Warren CM Smith BM Blumenstock JA Jiang J Davis MM. The public health impact of parent-reported childhood food allergies in the United States. Pediatrics. (2019) 142:1–12. doi: 10.1542/peds.2019-2461PP PMC6317772 30455345

[4] Luccioli S Ross M Labiner-Wolfe J Fein SB. Maternally reported food allergies and other food-related health problems in infants: characteristics and associated factors. Pediatrics. (2008) 122 Suppl 2:S105–12. doi: 10.1542/peds.2008-1315n, PMID: 18829825

[5] Zeiger RS Sampson HA Bock SA Burks AW Harden K Noone S. Soy allergy in infants and children with IgE-associated cow’s milk allergy. J Pediatr. (1999) 134:614–22. doi: 10.1016/S0022-3476(99)70249-0, PMID: 10228298

[6] Klemola T Vanto T Juntunen-Backman K Kalimo K Korpela R Varjonen E. Allergy to soy formula and to extensively hydrolyzed whey formula in infants with cow’s milk allergy: a prospective, randomized study with a follow-up to the age of 2 years. J Pediatr. (2002) 140:219–24. doi: 10.1067/mpd.2002.121935, PMID: 11865274

[7] Jarvinen K-M Makinen-Kiljunen S Suomalainen H. Cow’s milk challenge through human milk evokes immune responses in infants with cow’s milk allergy. J Pediatr. (1999) 135:506–12. doi: 10.1016/S0022-3476(99)70175-7, PMID: 10518086

[8] Picariello G Addeo F Ferranti P Nocerino R Paparo L Passariello A. Antibody-independent identification of bovine milk-derived peptides in breast-milk. Food Funct. (2016) 7:3402–9. doi: 10.1039/c6fo00731g, PMID: 27396729 PMC4981550

[9] Picariello G De Cicco M Nocerino R Paparo L Mamone G Addeo F. Excretion of dietary cow’s milk derived peptides into breast milk. Front Nutr. (2019) 6:6. doi: 10.3389/fnut.2019.00025, PMID: 30931311 PMC6424006

[10] Macchiaverni P Rekima A van den Elsen L Renz H Verhasselt V. Allergen shedding in human milk: could it be key for immune system education and allergy prevention? J Allergy Clin Immunol. (2021) 148:679–88. doi: 10.1016/j.jaci.2021.07.012, PMID: 34310930

[11] Schocker F Scharf A Kull S Jappe U. Detection of the peanut allergens Ara h 2 and Ara h 6 in human breast milk: development of 2 sensitive and specific sandwich ELISA assays. Int Arch Allergy Immunol. (2017) 174:17–25. doi: 10.1159/000479388, PMID: 28950267

[12] Zhu J Garrigues L Van Den Toorn H Stahl B Heck AJR. Discovery and quantification of nonhuman proteins in human milk. J Proteome Res. (2019) 18:225–38. doi: 10.1021/acs.jproteome.8b00550, PMID: 30489082 PMC6326037

[13] World Health Organization. Breastfeeding. Available online at: https://www.who.int/health-topics/breastfeeding#tab=tab_2 (Accessed January 30, 2023).

[14] United Nations Children’s Fund Breastfeeding: a mother’s gift, for every child (2018) Available online at: https://data.unicef.org/resources/breastfeeding-a-mothers-gift-for-every-child/

[15] Ip S Chung M Raman G Chew P Magula N DeVine D. Breastfeeding and maternal and infant health outcomes in developed countries. Evid Rep Technol Assess (2007) 1–186. Available online at: http://www.ncbi.nlm.nih.gov/pubmed/17764214 PMC4781366 17764214

[16] Binns C Lee M Low WY. The long-term public health benefits of breastfeeding. Asia Pac J Public Health. (2016) 28:7–14. doi: 10.1177/1010539515624964, PMID: 26792873

[17] Arvola T Holmberg-Marttila D. Benefits and risks of elimination diets. Ann Med. (1999) 31:293–8. doi: 10.3109/07853899908995893, PMID: 10480761

[18] Yilmaz O Kacar AS Gogebakan E Can C Necef I Mutluer T. The relationship between dietary elimination and maternal psychopathology in breastfeeding mothers of infants with food allergy. Pediatr Allergy Immunol. (2022) 33:e13670–8. doi: 10.1111/pai.13670, PMID: 34543481

[19] Franco C Fente C Sánchez C Lamas A Cepeda A Leis R. Cow’s milk antigens content in human milk: a scoping review. Foods. (2022) 11:1783. doi: 10.3390/foods11121783, PMID: 35741982 PMC9222876

[20] Perez-Riverol Y Bandla C Kundu DJ Kamatchinathan S Bai J Hewapathirana S. The PRIDE database at 20 years: 2025 update. Nucleic Acids Res. (2024) 53:D543–53. doi: 10.1093/nar/gkae1011, PMID: 39494541 PMC11701690

[21] R Core Team R: a language and environment for statistical computing (2024). Available online at: https://www.r-project.org/

[22] Subar AF Kirkpatrick SI Mittl B Zimmerman TP Thompson FE Bingley C. The Automated Self-Administered 24-hour dietary recall (ASA24): a resource for researchers, clinicians, and educators from the National Cancer Institute. J Acad Nutr Diet. (2012) 112:1134–7. doi: 10.1016/j.jand.2012.04.016 22704899 PMC3721511

[23] Brooks ME Kristensen K van Benthem KJ Magnusson A Berg CW Nielsen A. glmmTMB balances speed and flexibility among packages for zero-inflated generalized linear mixed modeling. RJ. (2017) 9:378–400. doi: 10.32614/RJ-2017-066

[24] Hartig F. _DHARMa: residual diagnostics for hierarchical (multi-level / mixed) regression models_. R Packag version 046 (2022). Available online at: https://cran.r-project.org/package=DHARMa

[25] Lenth R. _emmeans: Estimated marginal means, aka least-squares means_ (2024). Available online at: https://cran.r-project.org/package=emmeans

[26] Oksanen J Simpson G Blanchet F Kindt R Legendre P Minchin P. _vegan: community ecology package_. (2024) Available online at: https://cran.r-project.org/package=vegan

[27] McMurdie H. Phyloseq: an R package for reproducible interactive analysis and graphics of microbiome census data. PLoS One. (2013) 8:e61217. doi: 10.1371/journal.pone.0061217, PMID: 23630581 PMC3632530

[28] Institute of Medicine, Food and Nutrition Board. Dietary reference intakes for thiamin, riboflavin, niacin, vitamin B6, folate, vitamin B12, pantothenic acid, biotin and choline. Washington, DC: National Academies Press (1998). 23193625

[29] Institute of Medicine. Dietary reference intakes for vitamin C, vitamin E, selenium, and carotenoids. Washington, DC: The National Academies Press (2000). 25077263

[30] Institute of Medicine. Dietary reference intakes for calcium and vitamin D. Washington, DC: The National Academies Press (2011). 21796828

[31] Institute of Medicine. Dietary reference intakes: The essential guide to nutrient requirements. Washington, DC: The National Academies Press (2006).

[32] Sorva R Mlkinen-kiljunen S. Beta-lactoglobulin secretion in human milk varies widely after cow’s milk ingestion in mothers of infants with cow’s milk allergy. J Allergy Clin Immunol. (1994) 93:787–92. doi: 10.1016/0091-6749(94)90259-3 8163788

[33] Kilshaw PJ Cant AJ. The passage of maternal dietary proteins into human breast milk. Int Arch Allergy Appl Immunol. (1984) 75:8–15. doi: 10.1159/000233582, PMID: 6746107

[34] Dekker PM Boeren S Wijga AH Koppelman GH Vervoort JJM Hettinga KA. Maternal allergy and the presence of nonhuman proteinaceous molecules in human milk. Nutrients. (2020) 12:1–17. doi: 10.3390/nu12041169 PMC7230597 32331315

[35] Munblit D Perkin MR Palmer DJ Allen KJ Boyle RJ. Assessment of evidence about common infant symptoms and cow’s milk allergy. JAMA Pediatr. (2020) 174:599–608. doi: 10.1001/jamapediatrics.2020.0153, PMID: 32282040

[36] Gamirova A Berbenyuk A Levina D Peshko D Simpson MR Azad MB. Food proteins in human breast milk and probability of IgE-mediated allergic reaction in children during breastfeeding: a systematic review. J Allergy Clin Immunol Pract. (2022) 10:1312–1324.e8. doi: 10.1016/j.jaip.2022.01.028, PMID: 35123103

[37] Price D Ackland L Suphioglu C. Nuts ‘n’ guts: transport of food allergens across the intestinal epithelium. Asia Pac Allergy. (2013) 3:257–65. doi: 10.5415/apallergy.2013.3.4.257, PMID: 24260731 PMC3826608

[38] Fukushima Y Kawata Y Onda T Kitagawa M. Consumption of cow milk and egg by lactating women and the presence of beta-lactoglobulin and ovalbumin in breast milk. Am J Clin Nutr. (1997) 65:30–5. doi: 10.1093/ajcn/65.1.30, PMID: 8988909

[39] Franke AA Custer LJ Tanaka Y. Isoflavones in human breast milk and other biological fluids. Am J Clin Nutr. (1998) 68:1466S–73S. doi: 10.1093/ajcn/68.6.1466S, PMID: 9848518

[40] Franke AA Halm BM Custer LJ Tatsumura Y Hebshi S. Isoflavones in breastfed infants after mothers consume soy. Am J Clin Nutr. (2006) 84:406–13. doi: 10.1093/ajcn/84.2.406, PMID: 16895891

[41] Franke AA Custer LJ. Daidzein and genistein concentrations in human milk after soy consumption. Clin Chem. (1996) 42:955–64. doi: 10.1093/clinchem/42.6.955, PMID: 8665689

