# Supplementary material for: Detection of bovine milk–, but likely not soy–derived, peptides in human milk after maternal consumption of bovine milk and soy beverage: a randomized, cross-over, dietary intervention trial
Source: Front Nutr. 2025 Oct 23;12:1642177. doi: 10.3389/fnut.2025.1642177 (PMC12590503; doi:10.3389/fnut.2025.1642177)
Supplement: Supplementary file 1 [file Table_1.docx]

| **Supplementary Table 1.** Sequence, raw abundance, and relative abundance (%; range 0-100) of all bovine-derived peptides identified in human milk samples collected after 5 days of dietary bovine milk elimination and 2 and 4 hours after bovine milk consumption. BovE represents d6/d16 hr 0 collection after 5 days of dietary elimination. Bov2Hr and Bov4Hr represent d8/d18 hr 2 and 4 (respectively) after consuming 415mL of bovine milk. | | | | | | | | | |
| --- | --- | --- | --- | --- | --- | --- | --- | --- | --- |
| **Protein ID** | **Parent Protein** | **Peptide Sequence** | **Positions in Proteins** | **Number of Samples Total** | **Number of Samples BovE** | **Number of Samples Bov2Hr** | **Number of Samples Bov4Hr** | **Mean Raw Abundance** | **Mean Relative Abundance** |
| P02754 | Beta-lactoglobulin | TPEVDDEALEK | P02754 [141-151] | 70 | 23 | 23 | 24 | 1556728.49 | 0.26 |
| P02754 | Beta-lactoglobulin | ALNENKVL | P02754 [102-109] | 70 | 23 | 24 | 23 | 192666.70 | 0.17 |
| P02754 | Beta-lactoglobulin | KPTPEGDLEIL | P02754 [63-73] | 69 | 23 | 23 | 23 | 1422.44 | 0.14 |
| P02754 | Beta-lactoglobulin | VEELKPTPEGDLEIL | P02754 [59-73] | 72 | 24 | 24 | 24 | 36447.36 | 0.12 |
| P02754 | Beta-lactoglobulin | VLDTDYKKY | P02754 [110-118] | 57 | 19 | 20 | 18 | 8116.57 | 0.07 |
| P02668 | Kappa-casein | QDKTEIPTIN | P02668 [135-144] | 69 | 24 | 22 | 23 | 29823.51 | 0.06 |
| Q2KI46 | CDC42 effector protein (Rho GTPase binding) 3 | PLLSPVTFSSKQ | Q2KI46 [121-132] | 45 | 15 | 15 | 15 | 2872.88 | 0.06 |
| P02668 | Kappa-casein | NQDKTEIPTIN | P02668 [134-144] | 67 | 21 | 23 | 23 | 903.86 | 0.04 |
| P02754 | Beta-lactoglobulin | LDTDYKKY | P02754 [111-118] | 42 | 15 | 13 | 14 | 8617.30 | 0.04 |
| P02754 | Beta-lactoglobulin | KPTPEGDLEILL | P02754 [63-74] | 71 | 24 | 24 | 23 | 5817.61 | 0.04 |
| P02754 | Beta-lactoglobulin | ELKPTPEGDLEIL | P02754 [61-73] | 70 | 24 | 24 | 22 | 2615.89 | 0.03 |
| P02754 | Beta-lactoglobulin | RTPEVDDEALEK | P02754 [140-151] | 49 | 17 | 15 | 17 | 61196.04 | 0.02 |
| P02754 | Beta-lactoglobulin | VRTPEVDDEALEK | P02754 [139-151] | 52 | 16 | 19 | 17 | 222366.57 | 0.02 |
| P02754 | Beta-lactoglobulin | AEKTKIPAVF | P02754 [89-98] | 18 | 5 | 5 | 8 | 3313.05 | 0.02 |
| P02754 | Beta-lactoglobulin | TPEVDDEALEKF | P02754 [141-152] | 33 | 11 | 10 | 12 | 17938.53 | 0.02 |
| G9G9X6 | Alpha-lactalbumin protein variant D | LDKVGIN | G9G9X6 [115-121]; P00711 [115-121] | 39 | 13 | 14 | 12 | 4738.49 | 0.01 |
| P02668 | Kappa-casein | QDKTEIPTINT | P02668 [135-145] | 45 | 13 | 15 | 17 | 7369.48 | 0.01 |
| P02754 | Beta-lactoglobulin | IIAEKTKIPA | P02754 [87-96] | 35 | 13 | 12 | 10 | 4007.91 | 0.01 |
| P02662 | Alpha-S1-casein | HIQKEDVPSER | P02662 [95-105] | 58 | 18 | 20 | 20 | 4886.93 | 0.01 |
| P02754 | Beta-lactoglobulin | VYVEELKPTPEGDLEILLQ | P02754 [57-75] | 10 | 3 | 2 | 5 | 2006.84 | 0.01 |
| P02662 | Alpha-S1-casein | RPKHPIKHQGLPQ | P02662 [16-28] | 26 | 11 | 8 | 7 | 8080.26 | 0.01 |
| P02668 | Kappa-casein | NQDKTEIPTINT | P02668 [134-145] | 24 | 8 | 7 | 9 | 1274604.01 | 0.01 |
| P02754 | Beta-lactoglobulin | VEELKPTPEGDLEILL | P02754 [59-74] | 37 | 11 | 12 | 14 | 2286.01 | 0.01 |
| P02754 | Beta-lactoglobulin | YVEELKPTPEGDLEIL | P02754 [58-73] | 26 | 9 | 9 | 8 | 40312.06 | 0.01 |
| P02754 | Beta-lactoglobulin | VRTPEVDDEALEKFDKA | P02754 [139-155] | 56 | 18 | 18 | 20 | 14945.76 | 0.01 |
| P02666 | Beta-casein | NIPPLTQTPVVVPPF | P02666 [88-102] | 20 | 7 | 5 | 8 | 11289.81 | 0.01 |
| P02754 | Beta-lactoglobulin | RTPEVDDEALEKFDKA | P02754 [140-155] | 20 | 9 | 4 | 7 | 72744.61 | 0.01 |
| P02754 | Beta-lactoglobulin | TPEVDDEALEKFDKA | P02754 [141-155] | 10 | 6 | 2 | 2 | 12970.41 | <0.01 |
| P02754 | Beta-lactoglobulin | DAQSAPLRVY | P02754 [49-58] | 54 | 17 | 16 | 21 | 49913.03 | <0.01 |
| P02662 | Alpha-S1-casein | FFVAPFPEVFGK | P02662 [38-49] | 19 | 5 | 7 | 7 | 5684.53 | <0.01 |
| P02668 | Kappa-casein | KNQDKTEIPTIN | P02668 [133-144] | 8 | 3 | 2 | 3 | 9152.92 | <0.01 |
| P02666 | Beta-casein | EPVLGPVRGPFPIIV | P02666 [210-224] | 13 | 5 | 5 | 3 | 5284.68 | <0.01 |
| P02668 | Kappa-casein | PPKKNQDKTEIPTINTIA | P02668 [130-147] | 24 | 8 | 5 | 11 | 473267.21 | <0.01 |
| P02754 | Beta-lactoglobulin | KIIAEKTKIPAVF | P02754 [86-98] | 23 | 7 | 6 | 10 | 14553.16 | <0.01 |
| P02754 | Beta-lactoglobulin | LIVTQTMKG | P02754 [17-25] | 3 | 2 | 1 | 0 | 5052.04 | <0.01 |
| P02754 | Beta-lactoglobulin | VEELKPTPEGDLEILLQ | P02754 [59-75] | 26 | 11 | 6 | 9 | 57994.68 | <0.01 |
| P02754 | Beta-lactoglobulin | VLVLDTDYK | P02754 [108-116] | 18 | 4 | 6 | 8 | 3258.09 | <0.01 |
| P02754 | Beta-lactoglobulin | VEELKPTPEGDLE | P02754 [59-71] | 18 | 7 | 6 | 5 | 532.33 | <0.01 |
| P02754 | Beta-lactoglobulin | YVEELKPTPEGDLEILL | P02754 [58-74] | 12 | 3 | 7 | 2 | 9665.99 | <0.01 |
| G9G9X6 | Alpha-lactalbumin protein variant D | KILDKVGIN | G9G9X6 [113-121]; P00711 [113-121] | 21 | 7 | 6 | 8 | 13195.32 | <0.01 |
| P02754 | Beta-lactoglobulin | IPAVFKID | P02754 [94-101] | 10 | 4 | 3 | 3 | 58163.09 | <0.01 |
| P02754 | Beta-lactoglobulin | ALNENKVLVLDTDYKKY | P02754 [102-118] | 39 | 12 | 11 | 16 | 12579.99 | <0.01 |
| P02754 | Beta-lactoglobulin | KPTPEGDLEILLQ | P02754 [63-75] | 26 | 9 | 8 | 9 | 134374.20 | <0.01 |
| P02666 | Beta-casein | YQEPVLGPVR | P02666 [208-217] | 24 | 10 | 4 | 10 | 31041.66 | <0.01 |
| P02754 | Beta-lactoglobulin | IDALNENKVL | P02754 [100-109] | 30 | 9 | 8 | 13 | 83532.05 | <0.01 |
| P02663 | Alpha-S2-casein | SIISQETYK | P02663 [28-36] | 5 | 2 | 0 | 3 | 36150.88 | <0.01 |
| P02754 | Beta-lactoglobulin | AASDISLLDAQSAPLR | P02754 [41-56] | 55 | 16 | 20 | 19 | 6708.53 | <0.01 |
| P02666 | Beta-casein | MPFPKYPVEP | P02666 [124-133] | 32 | 10 | 12 | 10 | 11485.82 | <0.01 |
| P02754 | Beta-lactoglobulin | VYVEELKPTPEGDLEIL | P02754 [57-73] | 36 | 16 | 9 | 11 | 6083.97 | <0.01 |
| P02754 | Beta-lactoglobulin | IDALNENKVLV | P02754 [100-110] | 23 | 9 | 5 | 9 | 1829.75 | <0.01 |
| Q95114 | Lactadherin | SGLKINLFDTPLETQYVRLVP | Q95114 [229-249] | 19 | 4 | 5 | 10 | 11557.51 | <0.01 |
| P02666 | Beta-casein | SLSQSKVLPVPQ | P02666 [179-190] | 9 | 3 | 3 | 3 | 11317.52 | <0.01 |
| P02666 | Beta-casein | MPFPKYPVEPF | P02666 [124-134] | 30 | 13 | 7 | 10 | 15046.72 | <0.01 |
| P02666 | Beta-casein | RELEELNVPGEIVES | P02666 [16-30] | 14 | 6 | 4 | 4 | 517375.29 | <0.01 |
| P02754 | Beta-lactoglobulin | VLVLDTDYKK | P02754 [108-117] | 34 | 12 | 12 | 10 | 98858.32 | <0.01 |
| P02754 | Beta-lactoglobulin | FNPTQLEEQCHI | P02754 [167-178] | 41 | 14 | 15 | 12 | 59250.28 | <0.01 |
| P02754 | Beta-lactoglobulin | LDAQSAPLRVY | P02754 [48-58] | 23 | 6 | 6 | 11 | 75276.56 | <0.01 |
| P02754 | Beta-lactoglobulin | SFNPTQLEEQCHI | P02754 [166-178] | 53 | 18 | 16 | 19 | 11722.07 | <0.01 |
| P80195 | Glycosylation-dependent cell adhesion molecule 1 | LISKEQIVIR | P80195 [62-71] | 25 | 10 | 6 | 9 | 28218.30 | <0.01 |
| P02666 | Beta-casein | QEPVLGPVRGPFPIIV | P02666 [209-224] | 2 | 1 | 0 | 1 | 4989.94 | <0.01 |
| P02754 | Beta-lactoglobulin | RTPEVDDEALEKF | P02754 [140-152] | 18 | 7 | 6 | 5 | 8414.02 | <0.01 |
| P02754 | Beta-lactoglobulin | SLAMAASDISLLDAQSAPLR | P02754 [37-56] | 22 | 6 | 9 | 7 | 5076.21 | <0.01 |
| P02754 | Beta-lactoglobulin | LNENKVLVLDTDYKKY | P02754 [103-118] | 14 | 8 | 4 | 2 | 443569.51 | <0.01 |
| P02666 | Beta-casein | TLTDVENLHLPLPL | P02666 [141-154] | 17 | 5 | 7 | 5 | 9833.90 | <0.01 |
| P02666 | Beta-casein | YQEPVLGPVRGPFP | P02666 [208-221] | 9 | 4 | 3 | 2 | 6303.48 | <0.01 |
| P02668 | Kappa-casein | DKTEIPTINT | P02668 [136-145] | 14 | 5 | 4 | 5 | 65042.41 | <0.01 |
| P02754 | Beta-lactoglobulin | VRTPEVDDEALEKF | P02754 [139-152] | 19 | 8 | 5 | 6 | 2601.85 | <0.01 |
| G9G9X6 | Alpha-lactalbumin protein variant D | KGYGGVSLPEW | G9G9X6 [35-45]; P00711 [35-45] | 6 | 2 | 2 | 2 | 7010.04 | <0.01 |
| P80195 | Glycosylation-dependent cell adhesion molecule 1 | SSRQPQSQNPKLPLS | P80195 [72-86] | 20 | 7 | 7 | 6 | 17818.33 | <0.01 |
| P02754 | Beta-lactoglobulin | LVRTPEVDDEALE | P02754 [138-150] | 2 | 0 | 2 | 0 | 11774.20 | <0.01 |
| P02662 | Alpha-S1-casein | EVLNENLLR | P02662 [29-37] | 32 | 9 | 11 | 12 | 12854.01 | <0.01 |
| P02754 | Beta-lactoglobulin | VYVEELKPTPEGDLEILL | P02754 [57-74] | 2 | 2 | 0 | 0 | 6053.73 | <0.01 |
| P02662 | Alpha-S1-casein | VAPFPEVFGKEK | P02662 [40-51] | 16 | 6 | 7 | 3 | 75220.20 | <0.01 |
| P02668 | Kappa-casein | MAIPPKKNQDKTEIPTINT | P02668 [127-145] | 18 | 10 | 3 | 5 | 10880.94 | <0.01 |
| P02754 | Beta-lactoglobulin | LVLDTDYKKY | P02754 [109-118] | 14 | 7 | 3 | 4 | 48866.96 | <0.01 |
| P02754 | Beta-lactoglobulin | VEELKPTPE | P02754 [59-67] | 7 | 2 | 3 | 2 | 1945451.77 | <0.01 |
| P02754 | Beta-lactoglobulin | LIVTQTMK | P02754 [17-24] | 23 | 10 | 7 | 6 | 371244.77 | <0.01 |
| P02754 | Beta-lactoglobulin | IDALNENKVLVLDTDYKKY | P02754 [100-118] | 15 | 5 | 6 | 4 | 327668.53 | <0.01 |
| P80195 | Glycosylation-dependent cell adhesion molecule 1 | LGSEETTEHTPSDASTTEGK | P80195 [98-117] | 8 | 3 | 3 | 2 | 1026.15 | <0.01 |
| P02666 | Beta-casein | GVSKVKEAMAPK | P02666 [109-120] | 13 | 4 | 3 | 6 | 3666.47 | <0.01 |
| P02666 | Beta-casein | RELEELNVPGE | P02666 [16-26] | 3 | 0 | 2 | 1 | 21957.37 | <0.01 |
| P02754 | Beta-lactoglobulin | VLDTDYKKYL | P02754 [110-119] | 11 | 5 | 2 | 4 | 358988.09 | <0.01 |
| P80195 | Glycosylation-dependent cell adhesion molecule 1 | ILNKPEDETHLEAQPTDASAQFIRN | P80195 [19-43] | 13 | 6 | 3 | 4 | 2604.31 | <0.01 |
| P02666 | Beta-casein | VPYPQRDMPIQ | P02666 [193-203] | 14 | 5 | 3 | 6 | 105346.07 | <0.01 |
| P02662 | Alpha-S1-casein | RPKHPIKHQ | P02662 [16-24] | 16 | 5 | 6 | 5 | 4516.61 | <0.01 |
| P02662 | Alpha-S1-casein | APFPEVFGKE | P02662 [41-50] | 8 | 3 | 1 | 4 | 2596.08 | <0.01 |
| P02754 | Beta-lactoglobulin | VLVLDTDYKKY | P02754 [108-118] | 17 | 6 | 6 | 5 | 18892.51 | <0.01 |
| P02754 | Beta-lactoglobulin | MAASDISLLDAQSAPLR | P02754 [40-56] | 16 | 2 | 6 | 8 | 18782.50 | <0.01 |
| P02662 | Alpha-S1-casein | LNENLLRF | P02662 [31-38] | 12 | 3 | 7 | 2 | 1799.56 | <0.01 |
| P80195 | Glycosylation-dependent cell adhesion molecule 1 | AQPTDASAQFIRN | P80195 [31-43] | 11 | 6 | 2 | 3 | 146269.71 | <0.01 |
| P02754 | Beta-lactoglobulin | ASDISLLDAQSAPLR | P02754 [42-56] | 6 | 2 | 2 | 2 | 4026.96 | <0.01 |
| P02754 | Beta-lactoglobulin | VEELKPTPEGD | P02754 [59-69] | 2 | 2 | 0 | 0 | 5863.15 | <0.01 |
| P02662 | Alpha-S1-casein | EVLNENLLRF | P02662 [29-38] | 4 | 2 | 2 | 0 | 1249277.93 | <0.01 |
| P02754 | Beta-lactoglobulin | VTQTMKGLDIQ | P02754 [19-29] | 9 | 3 | 2 | 4 | 22226.29 | <0.01 |
| P02668 | Kappa-casein | SGEPTSTPTTE | P02668 [148-158] | 2 | 1 | 0 | 1 | 8200.32 | <0.01 |
| P02662 | Alpha-S1-casein | YKVPQLEIVPNSAEER | P02662 [119-134] | 11 | 4 | 4 | 3 | 14145.61 | <0.01 |
| P02754 | Beta-lactoglobulin | NENKVLVLDTDYKKY | P02754 [104-118] | 8 | 5 | 1 | 2 | 49432.30 | <0.01 |
| P02668 | Kappa-casein | MAIPPKKNQDKTEIPT | P02668 [127-142] | 3 | 2 | 1 | 0 | 68816.57 | <0.01 |
| P02754 | Beta-lactoglobulin | VLDTDYKKYLLF | P02754 [110-121] | 4 | 3 | 1 | 0 | 80962.48 | <0.01 |
| P02754 | Beta-lactoglobulin | AASDISLLDAQSAPLRVY | P02754 [41-58] | 12 | 5 | 3 | 4 | 11686.42 | <0.01 |
| P80195 | Glycosylation-dependent cell adhesion molecule 1 | SRQPQSQNPKLPLS | P80195 [73-86] | 3 | 3 | 0 | 0 | 6181.58 | <0.01 |
| P02754 | Beta-lactoglobulin | AEKTKIPAVFKID | P02754 [89-101] | 6 | 4 | 1 | 1 | 40082.78 | <0.01 |
| P02662 | Alpha-S1-casein | APFPEVFGK | P02662 [41-49] | 5 | 2 | 1 | 2 | 4657.30 | <0.01 |
| P02662 | Alpha-S1-casein | VAPFPEVFGKE | P02662 [40-50] | 17 | 4 | 5 | 8 | 3037.57 | <0.01 |
| P02662 | Alpha-S1-casein | FVAPFPEVFGK | P02662 [39-49] | 3 | 2 | 1 | 0 | 4936.11 | <0.01 |
| P02662 | Alpha-S1-casein | FVAPFPEVFGKEKVNEL | P02662 [39-55] | 5 | 3 | 1 | 1 | 3813.91 | <0.01 |
| P02754 | Beta-lactoglobulin | YVEELKPTPEGDLE | P02754 [58-71] | 7 | 2 | 3 | 2 | 83703.78 | <0.01 |
| P02662 | Alpha-S1-casein | VLNENLLRF | P02662 [30-38] | 2 | 1 | 1 | 0 | 12155.33 | <0.01 |
| P02754 | Beta-lactoglobulin | MKGLDIQKVAGTWYSL | P02754 [23-38] | 2 | 0 | 1 | 1 | 65034.73 | <0.01 |
| P02662 | Alpha-S1-casein | ERYLGYLEQL | P02662 [104-113] | 3 | 1 | 1 | 1 | 631191.45 | <0.01 |
| P80195 | Glycosylation-dependent cell adhesion molecule 1 | RNLENTVKETIK | P80195 [127-138] | 1 | 1 | 0 | 0 | 69555.88 | <0.01 |
| P02754 | Beta-lactoglobulin | LVRTPEVDDE | P02754 [138-147] | 2 | 0 | 2 | 0 | 239694.66 | <0.01 |
| P02754 | Beta-lactoglobulin | ALNENKVLVLDTDYKK | P02754 [102-117] | 3 | 3 | 0 | 0 | 41508.30 | <0.01 |
| P02754 | Beta-lactoglobulin | LVRTPEVDDEALEKFDKA | P02754 [138-155] | 9 | 5 | 2 | 2 | 14823.73 | <0.01 |
| P02662 | Alpha-S1-casein | FVAPFPEVFGKE | P02662 [39-50] | 2 | 1 | 1 | 0 | 15472.22 | <0.01 |
| P02666 | Beta-casein | RELEELNVPGEIVE | P02666 [16-29] | 2 | 0 | 2 | 0 | 30840.15 | <0.01 |
| P80195 | Glycosylation-dependent cell adhesion molecule 1 | SSRQPQSQNPKLPLSIL | P80195 [72-88] | 2 | 2 | 0 | 0 | 277053.61 | <0.01 |
| P02754 | Beta-lactoglobulin | AMAASDISLLDAQSAPLRVY | P02754 [39-58] | 2 | 0 | 1 | 1 | 924.92 | <0.01 |
| P02662 | Alpha-S1-casein | KYKVPQLEIVPNSAEER | P02662 [118-134] | 3 | 1 | 1 | 1 | 2818.11 | <0.01 |
| P02754 | Beta-lactoglobulin | MAASDISLLDAQSAPLRVY | P02754 [40-58] | 1 | 0 | 1 | 0 | 3165.77 | <0.01 |
| P02662 | Alpha-S1-casein | VPSERYLGY | P02662 [101-109] | 1 | 1 | 0 | 0 | 40793.54 | <0.01 |

| **Supplementary Table 3.** Kruskal Wallis tests demonstrated no differences in mean relative abundances across the three collection time points for bovine-derived peptides aggregated at parent protein level and evaluated using Kruskal Wallis tests. BovE represents d6/d16 hr 0 collection after 5 days of dietary bovine milk elimination. Bov2Hr and Bov4Hr represent d8/d18 hr 2 and 4, respectively, after maternal consumption of 415 mL bovine milk during the reintroduction period. Means and standard deviations are reported in relative abundance (%; range 0-100). | | | | | | | |
| --- | --- | --- | --- | --- | --- | --- | --- |
| **Parent Protein** | **BovE Mean** | **BovE Standard Deviation** | **Bov2Hr Mean** | **Bov2Hr Standard Deviation** | **Bov4Hr Mean** | **Bov4Hr Standard Deviation** | **P value** |
| Beta-lactoglobulin | 1.21 | 2.30 | 1.19 | 4.08 | 0.78 | 1.49 | 0.83 |
| Kappa-Casein | 0.13 | 0.31 | 0.15 | 0.61 | 0.13 | 0.35 | 0.51 |
| Alpha-S1-Casein | 0.03 | 0.05 | 0.03 | 0.04 | 0.03 | 0.07 | 0.40 |
| Beta-Casein | 0.03 | 0.05 | 0.02 | 0.03 | 0.03 | 0.08 | 0.64 |
| Alpha-lactalbumin protein variant D | 0.01 | 0.02 | 0.02 | 0.11 | 0.01 | 0.04 | 0.68 |
| Glycosylation-dependent cell adhesion molecule 1 | 0.01 | 0.02 | 0.00 | 0.00 | 0.00 | 0.00 | 0.76 |

| **Supplementary Table 4**. Kruskal Wallis tests demonstrated no differences in relative abundances across the three collection time points for six bovine-derived peptides evaluated using Kruskal Wallis tests. BovE represents d6/d16 hr 0 collection after 5 days of dietary bovine milk elimination. Bov2Hr and Bov4Hr represent d8/d18 hr 2 and 4, respectively, after maternal consumption of 415 mL bovine milk during the reintroduction period. Means and standard deviations are reported in relative abundance (%; range 0-100). | | | | | | | | |
| --- | --- | --- | --- | --- | --- | --- | --- | --- |
| **Parent Protein** | **Peptide** | **BovE Mean** | **BovE Standard Deviation** | **Bov2Hr Mean** | **Bov2Hr Standard Deviation** | **Bov4Hr Mean** | **Bov4Hr Standard Deviation** | **P value** |
| Beta-lactoglobulin | ELKPTPEGDLEIL | 0.02 | 0.05 | 0.04 | 0.14 | 0.01 | 0.03 | 0.93 |
| Beta-lactoglobulin | ALNENKVL | 0.28 | 0.71 | 0.12 | 0.32 | 0.12 | 0.26 | 0.58 |
| Beta-lactoglobulin | TPEVDDEALEK | 0.08 | 0.12 | 0.51 | 2.28 | 0.20 | 0.76 | 0.45 |
| Beta-lactoglobulin | KPTPEGDLEIL | 0.14 | 0.31 | 0.19 | 0.75 | 0.09 | 0.17 | 0.81 |
| Kappa-casein | QDKTEIPTIN | 0.03 | 0.05 | 0.11 | 0.46 | 0.06 | 0.22 | 0.60 |
| Kappa-casein | NQDKTEIPTIN | 0.06 | 0.18 | 0.03 | 0.07 | 0.04 | 0.14 | 0.56 |

| **Supplementary Table 5.** List of the soy-derived peptides identified from one soybean milk sample analyzed in duplicate. | | | | | |
| --- | --- | --- | --- | --- | --- |
| **Protein ID** | **Protein** | **Peptide sequence** | **Positions in Proteins** | **Theoretical MH+ (Dalton)** | **Average Raw Abundance** |
| P04405 | Glycinin G2 | PFSFLVPPQESQRR | P04405 [469-482] | 1687.8915 | 5.15.E+08 |
| P05046 | Lectin | PLDLTSFVLHEAI | P05046 [273-285] | 1454.7890 | 4.30.E+08 |
| P04405 | Glycinin G2 | PFSFLVPPQESQRRA | P04405 [469-483] | 1758.9286 | 2.77.E+08 |
| P04405 | Glycinin G2 | NPFSFLVPPQESQRR | P04405 [468-482] | 1801.9344 | 1.79.E+08 |
| P04776 | Glycinin G1 | PFKFLVPPQESQKR | P04776 [479-492] | 1700.9483 | 1.64.E+08 |
| P04776 | Glycinin G1 | PFKFLVPPQESQKRA | P04776 [479-493] | 1771.9854 | 1.37.E+08 |
| P04405 | Glycinin G2 | NNPFSFLVPPQESQRR | P04405 [467-482] | 1915.9773 | 1.04.E+08 |
| P05046 | Lectin | PLDLTSFVLHEA | P05046 [273-284] | 1341.7049 | 9.79.E+07 |
| P04776 | Glycinin G1 | NPFKFLVPPQESQKR | P04776 [478-492] | 1814.9912 | 9.79.E+07 |
| P04405 | Glycinin G2 | NPFSFLVPPQESQRRA | P04405 [468-483] | 1872.9715 | 7.96.E+07 |
| P04776 | Glycinin G1 | NNPFKFLVPPQESQKR | P04776 [477-492] | 1929.0341 | 7.20.E+07 |
| P04776 | Glycinin G1 | NPFKFLVPPQESQKRA | P04776 [478-493] | 1886.0283 | 6.88.E+07 |
| P04347 | Glycinin G5 | LKYQGNSGPLVNP | P04347 [504-516] | 1386.7376 | 6.39.E+07 |
| P01070 | Trypsin inhibitor A | DFVLDNEGNPLEN | P01070 [25-37] | 1475.6649 | 6.08.E+07 |
| P04405 | Glycinin G2 | NNPFSFLVPPQESQRRA | P04405 [467-483] | 1987.0144 | 5.60.E+07 |
| P13917 | Basic 7S globulin | VTPTKPINLVVLPVQN | P13917 [25-40] | 1732.0367 | 5.55.E+07 |
| P02858 | Glycinin G4 | WGPLVNPESQQGSPR | P02858 [545-559] | 1651.8187 | 5.50.E+07 |
| P04776 | Glycinin G1 | NNPFKFLVPPQESQKRA | P04776 [477-493] | 2000.0712 | 5.09.E+07 |
| Q01417 | 18 kDa seed maturation protein | MQGGKKAGESIKETATNIGAS | Q01417 [1-21] | 2120.0652 | 5.02.E+07 |
| P29531 | P24 oleosin isoform B | TTVPPHSVQVH | P29531 [2-12] | 1243.6430 | 4.69.E+07 |
| P04405 | Glycinin G2 | GIDETICTMRLRQNIGQN | P04405 [301-318] | 2119.0383 | 4.68.E+07 |
| P04405; P04776 | Glycinin G2; Glycinin G1 | SLLNALPEEVIQHTFNLKSQQARQ | P04405 [440-463]; P04776 [450-473] | 2764.4741 | 4.33.E+07 |
| P04405; P04776 | Glycinin G2; Glycinin G1 | SLENQLDQMPRRF | P04405 [167-179]; P04776 [170-182] | 1633.8115 | 4.23.E+07 |
| P04405; P04776 | Glycinin G2; Glycinin G1 | ALPEEVIQHTFNLKSQQARQ | P04405 [444-463]; P04776 [454-473] | 2337.2310 | 4.10.E+07 |
| P04776 | Glycinin G1 | FLVPPQESQKR | P04776 [482-492] | 1328.7321 | 4.06.E+07 |
| P04405 | Glycinin G2 | PFSFLVPPQESQR | P04405 [469-481] | 1531.7904 | 4.02.E+07 |
| P04405; P04776 | Glycinin G2; Glycinin G1 | ALPEEVIQHTFN | P04405 [444-455]; P04776 [454-465] | 1397.7060 | 4.00.E+07 |
| P29530 | P24 oleosin isoform A | TTQVPPHSVQVH | P29530 [2-13] | 1371.7015 | 3.90.E+07 |
| P08170 | Seed linoleate 13S-lipoxygenase-1 | MFSAGHKIKGTVVLMPKNELEVN | P08170 [1-23] | 2584.3626 | 3.59.E+07 |
| P04347 | Glycinin G5 | SYNLGQSQVRQ | P04347 [493-503] | 1279.6389 | 3.45.E+07 |
| P08170 | Seed linoleate 13S-lipoxygenase-1 | PSLQGNRLGPVQLPYTLLYPSSEEGLTFRGIPNSISI | P08170 [803-839] | 4014.1335 | 3.36.E+07 |
| Q39846 | Seed biotin-containing protein SBP65 | ASEQLARREN | Q39846 [2-11] | 1215.6076 | 3.33.E+07 |
| P04776 | Glycinin G1 | PFKFLVPPQESQK | P04776 [479-491] | 1544.8471 | 3.19.E+07 |
| Q8RVH5 | Basic 7S globulin 2 | VPIPQHHTNPTKPINLLVLPVQN | Q8RVH5 [25-47] | 2569.4613 | 3.07.E+07 |
| P04776 | Glycinin G1 | FLVPPQESQKRA | P04776 [482-493] | 1399.7692 | 3.04.E+07 |
| P04405; P04776 | Glycinin G2; Glycinin G1 | SLLNALPEEVIQHTFNLKS | P04405 [440-458]; P04776 [450-468] | 2153.1601 | 2.87.E+07 |
| P02858 | Glycinin G4 | WGPLVNPESQQGSP | P02858 [545-558] | 1495.7176 | 2.85.E+07 |
| Q01417 | 18 kDa seed maturation protein | TRAGGKPNDYGYGTGGT | Q01417 [157-173] | 1671.7721 | 2.75.E+07 |
| P04405 | Glycinin G2 | DEEEQPQCVETDKGCQRQS | P04405 [277-295] | 2322.9561 | 2.62.E+07 |
| Q01417 | 18 kDa seed maturation protein | MQGGKKAGESIKETA | Q01417 [1-15] | 1576.7999 | 2.57.E+07 |
| P13917 | Basic 7S globulin | SIRINQHSVFPL | P13917 [260-271] | 1410.7852 | 2.56.E+07 |
| P05046 | Lectin | FVLHEAI | P05046 [279-285] | 828.4614 | 2.48.E+07 |
| P04776 | Glycinin G1 | GIDETICTMRLRHNIGQTSSPDIYN | P04776 [311-335] | 2891.3775 | 2.42.E+07 |
| P04405 | Glycinin G2 | FLVPPQESQRR | P04405 [472-482] | 1356.7383 | 2.37.E+07 |
| P01071; P01070 | Trypsin inhibitor B; Trypsin inhibitor A | DFVLDNEGN | P01071 [1-9]; P01070 [25-33] | 1022.4425 | 2.36.E+07 |
| Q39837 | Albumin-1 | ADCNGACSPFEVPPCRSRDCRCVPIGLFVGFCIHPTG | Q39837 [20-56] | 4265.9017 | 2.30.E+07 |
| P04405; P04776 | Glycinin G2; Glycinin G1 | SLLNALPEEVIQHTFNLK | P04405 [440-457]; P04776 [450-467] | 2066.1281 | 2.30.E+07 |
| Q8RVH5 | Basic 7S globulin 2 | VPIPQHHTNPTKPIN | Q8RVH5 [25-39] | 1692.9180 | 2.28.E+07 |
| P11828 | Glycinin G3 | PFSFLVPPKESQRR | P11828 [465-478] | 1687.9279 | 2.22.E+07 |
| P12810 | 16.9 kDa class I heat shock protein 1 | DLPGVKKEEV | P12810 [60-69] | 1113.6150 | 2.14.E+07 |
| P04405; P04776 | Glycinin G2; Glycinin G1 | ALPEEVIQHTFNLKS | P04405 [444-458]; P04776 [454-468] | 1725.9170 | 2.13.E+07 |
| P04405; P04776 | Glycinin G2; Glycinin G1 | SLLNALPEEVIQHTFN | P04405 [440-455]; P04776 [450-465] | 1824.9490 | 2.07.E+07 |
| P04405; P04776 | Glycinin G2; Glycinin G1 | SLLNALPEEVIQHTFNLKSQQA | P04405 [440-461]; P04776 [450-471] | 2480.3144 | 2.06.E+07 |
| P04405 | Glycinin G2 | EEEQPQCVETDKGCQRQS | P04405 [278-295] | 2207.9292 | 1.94.E+07 |
| P04405; P04776 | Glycinin G2; Glycinin G1 | LNALPEEVIQHTFNLKS | P04405 [442-458]; P04776 [452-468] | 1953.0440 | 1.94.E+07 |
| P08170 | Seed linoleate 13S-lipoxygenase-1 | DPSLQGNRLGPVQLPYTLLYPSSEEGLTFRGIPNSISI | P08170 [802-839] | 4129.1604 | 1.94.E+07 |
| P02858 | Glycinin G4 | GVEENICTLKLHENIARPSRADFYN | P02858 [379-403] | 2946.4527 | 1.93.E+07 |
| P04405; P04776 | Glycinin G2; Glycinin G1 | LAGANSLLNALPEEVIQHTFNLKS | P04405 [435-458]; P04776 [445-468] | 2579.3828 | 1.89.E+07 |
| P04405 | Glycinin G2 | SFLVPPQESQRR | P04405 [471-482] | 1443.7703 | 1.84.E+07 |
| P01070 | Trypsin inhibitor A | VFCPQQAEDDKCGDIG | P01070 [158-173] | 1838.7684 | 1.73.E+07 |
| Q39837 | Albumin-1 | ADCNGACSPFEVPPCRSRDCRCVPIGLFVGFCIHPT | Q39837 [20-55] | 4208.8802 | 1.66.E+07 |
| P04405; P04776 | Glycinin G2; Glycinin G1 | LNALPEEVIQHTFN | P04405 [442-455]; P04776 [452-465] | 1624.8330 | 1.58.E+07 |
| P05046 | Lectin | LPHASSNIDPLDLTSFVLHEAI | P05046 [264-285] | 2389.2398 | 1.57.E+07 |
| P08170 | Seed linoleate 13S-lipoxygenase-1 | MFSAGHKIKGTVVLMPKNELEVNPDGSAVD | P08170 [1-30] | 3225.6283 | 1.56.E+07 |
| P04776 | Glycinin G1 | NPFKFLVPPQESQK | P04776 [478-491] | 1658.8901 | 1.54.E+07 |
| P02858 | Glycinin G4 | LKYEGNWGPLVNPESQQGSP | P02858 [539-558] | 2200.0669 | 1.53.E+07 |
| P04405 | Glycinin G2 | LREQAQQNECQIQKLN | P04405 [19-34] | 1999.9978 | 1.50.E+07 |
| P05046 | Lectin | PLDLTSFVLH | P05046 [273-282] | 1141.6252 | 1.49.E+07 |
| P04405; P04776 | Glycinin G2; Glycinin G1 | LNALPEEVIQHTFNLKSQQA | P04405 [442-461]; P04776 [452-471] | 2280.1983 | 1.45.E+07 |
| P02858 | Glycinin G4 | LKYEGNWGPLVNPESQQGSPR | P02858 [539-559] | 2356.1680 | 1.45.E+07 |
| F7J077 | Beta-conglycinin beta subunit 2 | LKVREDENNPFYLRSSN | F7J077 [24-40]; P25974 [24-40] | 2081.0410 | 1.44.E+07 |
| P10538 | Beta-amylase | HAITPLKPSAPKIPIEVLLEATKPTLPFPWLPETDMKVDG | P10538 [457-496] | 4390.4135 | 1.44.E+07 |
| P04405; P04776 | Glycinin G2; Glycinin G1 | LNALPEEVIQHTFNLKSQQARQ | P04405 [442-463]; P04776 [452-473] | 2564.3580 | 1.42.E+07 |
| P04405; P04776 | Glycinin G2; Glycinin G1 | ALPEEVIQHTFNLKSQQA | P04405 [444-461]; P04776 [454-471] | 2053.0713 | 1.40.E+07 |
| P29530 | P24 oleosin isoform A | TTQVPPH | P29530 [2-8] | 821.4152 | 1.39.E+07 |
| P08170 | Seed linoleate 13S-lipoxygenase-1 | NDPSLQGNRLGPVQLPYTLLYPSSEEGLTFRGIPNSISI | P08170 [801-839] | 4243.2034 | 1.38.E+07 |
| P04405 | Glycinin G2 | ALPEEVIQHTFNLKSQQARQVKNNNPFSFLVPPQESQRR | P04405 [444-482] | 4575.3968 | 1.34.E+07 |
| P04347 | Glycinin G5 | SYNLGQSQVRQLKYQGNSGPLVNP | P04347 [493-516] | 2647.3587 | 1.34.E+07 |
| P29531 | P24 oleosin isoform B | TTVPPHSVQVHTTTH | P29531 [2-16] | 1683.8449 | 1.33.E+07 |
| P46519 | Desiccation protectant protein Lea14 homolog | SQLLDKAKN | P46519 [2-10] | 1058.5841 | 1.32.E+07 |
| P04776 | Glycinin G1 | NNPFKFLVPPQESQK | P04776 [477-491] | 1772.9330 | 1.30.E+07 |
| P04405 | Glycinin G2 | FLVPPQESQRRA | P04405 [472-483] | 1427.7754 | 1.29.E+07 |
| Q39837 | Albumin-1 | GFCIHPTG | Q9ZQX0 [49-56]; Q39837 [49-56] | 888.4033 | 1.28.E+07 |
| P11828 | Glycinin G3 | FSFREQPQQN | P11828 [20-29] | 1280.6018 | 1.28.E+07 |
| P04405; P04776 | Glycinin G2; Glycinin G1 | ALPEEVIQHTFNLK | P04405 [444-457]; P04776 [454-467] | 1638.8850 | 1.28.E+07 |
| P04405; P04776 | Glycinin G2; Glycinin G1 | ALPEEVIQHTFNL | P04405 [444-456]; P04776 [454-466] | 1510.7900 | 1.24.E+07 |
| P04405 | Glycinin G2 | FSFLVPPQESQRR | P04405 [470-482] | 1590.8387 | 1.24.E+07 |
| P02858 | Glycinin G4 | GVEENICTLKLHEN | P02858 [379-392] | 1655.8058 | 1.22.E+07 |
| P04405; P04776 | Glycinin G2; Glycinin G1 | LNALPEEVIQHTFNLK | P04405 [442-457]; P04776 [452-467] | 1866.0120 | 1.20.E+07 |
| P05046 | Lectin | LTSFVLHEAI | P05046 [276-285] | 1129.6252 | 1.19.E+07 |
| P29531 | P24 oleosin isoform B | TTVPPHSVQVHT | P29531 [2-13] | 1344.6906 | 1.19.E+07 |
| P04347 | Glycinin G5 | GVEENICTMKLHENIARPSRADFYN | P04347 [345-369] | 2964.4091 | 1.16.E+07 |
| P05046 | Lectin | PLDLTSFVLHE | P05046 [273-283] | 1270.6678 | 1.14.E+07 |
| P04405; P04776 | Glycinin G2; Glycinin G1 | LAGANSLLNALPEEVIQHTFNLK | P04405 [435-457]; P04776 [445-467] | 2492.3508 | 1.12.E+07 |
| P01070 | Trypsin inhibitor A | DFVLDNEGNPLENGGTYYILSDITAFGGIRAAPTGN | P01070 [25-60] | 3771.8137 | 1.12.E+07 |
| P04405 | Glycinin G2 | VKNNNPFSFLVPPQESQRR | P04405 [464-482] | 2257.1836 | 1.10.E+07 |
| P01064; P01063 | Bowman-Birk type proteinase inhibitor D-II; Bowman-Birk type proteinase inhibitor C-II | FCYKPC | P01064 [73-78]; P01063 [70-75] | 874.3586 | 1.08.E+07 |
| P04776 | Glycinin G1 | FSSREQPQQNECQIQKLN | P04776 [20-37] | 2234.0619 | 1.07.E+07 |
| P29531 | P24 oleosin isoform B | TTVPPHSVQVHTT | P29531 [2-14] | 1445.7383 | 1.04.E+07 |
| Q01417 | 18 kDa seed maturation protein | MQGGKKAGESIKETATN | Q01417 [1-17] | 1791.8905 | 1.04.E+07 |
| Q8RVH5 | Basic 7S globulin 2 | VPIPQHHTN | Q8RVH5 [25-33] | 1042.5429 | 1.01.E+07 |
| P29530 | P24 oleosin isoform A | TTQVPPHSVQVHT | P29530 [2-14] | 1472.7492 | 1.01.E+07 |
| P04405; P04776 | Glycinin G2; Glycinin G1 | SLLNALPEEVIQHTFNLKSQQ | P04405 [440-460]; P04776 [450-470] | 2409.2773 | 1.01.E+07 |
| P05046 | Lectin | IDPLDLTSFVLHEAI | P05046 [271-285] | 1682.9000 | 9.84.E+06 |
| P11828; P04405 | Glycinin G3; Glycinin G2 | RPSIGNLAGANSL | P11828 [425-437]; P04405 [429-441] | 1269.6910 | 9.66.E+06 |
| P04405; P04776 | Glycinin G2; Glycinin G1 | LAGANSLLNALPEEVIQHTFNLKSQQARQ | P04405 [435-463]; P04776 [445-473] | 3190.6967 | 9.42.E+06 |
| Q01417 | 18 kDa seed maturation protein | MQGGKKAGESIKET | Q01417 [1-14] | 1505.7628 | 9.32.E+06 |
| P05046 | Lectin | PPNPHIGINVN | P05046 [166-176] | 1171.6218 | 9.26.E+06 |
| P05046 | Lectin | SSNIDPLDLTSFVLHEAI | P05046 [268-285] | 1971.0070 | 9.22.E+06 |
| P09186; P08170 | Seed linoleate 9S-lipoxygenase-3; Seed linoleate 13S-lipoxygenase-1 | FRGIPNSISI | P09186 [848-857]; P08170 [830-839]; P38417 [844-853] | 1103.6208 | 9.01.E+06 |
| P01070 | Trypsin inhibitor A | VVQFQKLDKESL | P01070 [194-205] | 1433.7999 | 8.97.E+06 |
| P04405 | Glycinin G2 | NPFSFLVPPQESQR | P04405 [468-481] | 1645.8333 | 8.86.E+06 |
| P02858 | Glycinin G4 | ISSSKLNECQLN | P02858 [24-35] | 1392.6788 | 8.70.E+06 |
| P04405 | Glycinin G2 | DDEEEQPQCVETDKGCQRQS | P04405 [276-295] | 2437.9831 | 8.62.E+06 |
| P04405; P04776 | Glycinin G2; Glycinin G1 | LNALPEEVIQHTFNLKSQQ | P04405 [442-460]; P04776 [452-470] | 2209.1612 | 8.50.E+06 |
| P04405 | Glycinin G2 | SFLVPPQESQRRA | P04405 [471-483] | 1514.8074 | 8.47.E+06 |
| P08170 | Seed linoleate 13S-lipoxygenase-1 | AFLGRSVSLQ | P08170 [34-43] | 1077.6051 | 8.43.E+06 |
| P08170 | Seed linoleate 13S-lipoxygenase-1 | MFSAGHKIKGTVVLMPKNELEVNPDGSAVDN | P08170 [1-31] | 3339.6712 | 8.41.E+06 |
| P29530 | P24 oleosin isoform A | TTQVPPHSVQVHTT | P29530 [2-15] | 1573.7969 | 8.39.E+06 |
| P04405 | Glycinin G2 | LVPPQESQRRA | P04405 [473-483] | 1280.7070 | 8.21.E+06 |
| P04405 | Glycinin G2 | LVPPQESQRR | P04405 [473-482] | 1209.6698 | 8.13.E+06 |
| P04405 | Glycinin G2 | DRPSIGNLAGANSLLNALPEEVIQHTFNLKS | P04405 [428-458] | 3318.7441 | 8.06.E+06 |
| P04405; P04776 | Glycinin G2; Glycinin G1 | SLLNALPEEVIQHTFNL | P04405 [440-456]; P04776 [450-466] | 1938.0331 | 7.83.E+06 |
| P29530 | P24 oleosin isoform A | TTQVPPHSVQ | P29530 [2-11] | 1135.5742 | 7.69.E+06 |
| P04405; P04776 | Glycinin G2; Glycinin G1 | LAGANSLLNALPEEVIQHTFN | P04405 [435-455]; P04776 [445-465] | 2251.1717 | 7.45.E+06 |
| P11827 | Beta-conglycinin alpha' subunit | VISQIPSQVQELAFPGSAKDIENLIKSQSESYFVDAQPQQKEEGN | P11827 [562-606] | 4976.4800 | 7.33.E+06 |
| P29531 | P24 oleosin isoform B | TTVPPHSVQVHTTTHR | P29531 [2-17] | 1839.9460 | 7.29.E+06 |
| P04405 | Glycinin G2 | LREQAQQNECQIQKLNALKPDN | P04405 [19-40] | 2638.3366 | 7.27.E+06 |
| P01070 | Trypsin inhibitor A | VVQFQKLDKES | P01070 [194-204] | 1320.7158 | 7.27.E+06 |
| P02858 | Glycinin G4 | ISLLDTSNFNNQLDQTPRVF | P02858 [168-187] | 2322.1725 | 7.23.E+06 |
| Q39837 | Albumin-1 | ADCNGACSPFEVPPCRSRDCRCVPIGLFVGFCIHPTGL | Q39837 [20-57] | 4378.9858 | 7.19.E+06 |
| P11828; P04405; P04776 | Glycinin G3; Glycinin G2; Glycinin G1 | GIDETICTMRL | P11828 [297-307]; P04405 [301-311]; P04776 [311-321] | 1308.6286 | 7.07.E+06 |
| P29531 | P24 oleosin isoform B | TTVPPHSVQ | P29531 [2-10] | 1007.5156 | 7.06.E+06 |
| P08170 | Seed linoleate 13S-lipoxygenase-1 | RLGPVQLPYTLLYPSSEEGLTFRGIPNSISI | P08170 [809-839] | 3417.8417 | 7.03.E+06 |
| P04405 | Glycinin G2 | LKEAFGVN | P04405 [226-233] | 877.4778 | 6.99.E+06 |
| P04405 | Glycinin G2 | VKNNNPFSFLVPPQESQRRA | P04405 [464-483] | 2328.2207 | 6.78.E+06 |
| P04405 | Glycinin G2 | GIDETICTMRLRQNIG | P04405 [301-316] | 1876.9368 | 6.78.E+06 |
| P02858 | Glycinin G4 | PLVNPESQQGSPR | P02858 [547-559] | 1408.7179 | 6.76.E+06 |
| P22895 | P34 probable thiol protease | YFASYPTKEESETLVS | P22895 [350-365] | 1850.8695 | 6.73.E+06 |
| P04776 | Glycinin G1 | LVPPQESQKRA | P04776 [483-493] | 1252.7008 | 6.69.E+06 |
| Q01417 | 18 kDa seed maturation protein | AGGKPNDYGYGTGGT | Q01417 [159-173] | 1414.6234 | 6.69.E+06 |
| P04405 | Glycinin G2 | DEEEQPQCVETDKGCQRQ | P04405 [277-294] | 2235.9241 | 6.68.E+06 |
| P01064 | Bowman-Birk type proteinase inhibitor D-II | DFCYKPCKSRDD | P01064 [72-83] | 1590.6675 | 6.52.E+06 |
| P13917 | Basic 7S globulin | VTPTKPINLVVLPVQNDGSTGLHWAN | P13917 [25-50] | 2770.4886 | 6.40.E+06 |
| P22895 | P34 probable thiol protease | YFASYPTKEESET | P22895 [350-362] | 1551.6850 | 6.37.E+06 |
| Q01417 | 18 kDa seed maturation protein | MQGGKKAGESIKETATNIGASA | Q01417 [1-22] | 2191.1023 | 6.27.E+06 |
| P01055 | Bowman-Birk type proteinase inhibitor | FCYEPCKPSE | P01055 [96-105] | 1316.5286 | 6.25.E+06 |
| P04776 | Glycinin G1 | TPMIGTLAGANSL | P04776 [439-451] | 1245.6508 | 6.23.E+06 |
| P05046 | Lectin | IDPLDLTSFVLHEA | P05046 [271-284] | 1569.8159 | 6.15.E+06 |
| P05046 | Lectin | LPHASSNIDPLDLTS | P05046 [264-278] | 1579.7962 | 6.10.E+06 |
| P09186 | Seed linoleate 9S-lipoxygenase-3 | MLGGLLHRG | P09186 [1-9] | 995.5455 | 6.07.E+06 |
| P05046 | Lectin | IPGESHDVLSWSFASNLPHASSNID | P05046 [248-272] | 2680.2638 | 5.99.E+06 |
| Q01417 | 18 kDa seed maturation protein | MQGGKKAGESIKE | Q01417 [1-13] | 1404.7151 | 5.97.E+06 |
| P11828; P04405 | Glycinin G3; Glycinin G2 | DRPSIGNLAGANSL | P11828 [424-437]; P04405 [428-441] | 1384.7179 | 5.95.E+06 |
| P03596 | Movement protein | VPNTTISGIA | P03596 [258-267] | 1014.5466 | 5.92.E+06 |
| P04405 | Glycinin G2 | GIDETICTMRLRQNIGQNSSPDIYN | P04405 [301-325] | 2895.3724 | 5.91.E+06 |
| P04405 | Glycinin G2 | FSFLVPPQESQRRA | P04405 [470-483] | 1661.8758 | 5.78.E+06 |
| P04776 | Glycinin G1 | LVPPQESQKR | P04776 [483-492] | 1181.6637 | 5.72.E+06 |
| P04347 | Glycinin G5 | YQGNSGPLVNP | P04347 [506-516] | 1145.5586 | 5.72.E+06 |
| P04405; P04776 | Glycinin G2; Glycinin G1 | YLAGNQEQEFLKY | P04405 [180-192]; P04776 [183-195] | 1602.7799 | 5.61.E+06 |
| P04776 | Glycinin G1 | FKFLVPPQESQKR | P04776 [480-492] | 1603.8955 | 5.54.E+06 |
| P26413 | Heat shock 70 kDa protein | KSINPDEAVAYGAAVQA | P26413 [366-382] | 1703.8599 | 5.49.E+06 |
| Q01417 | 18 kDa seed maturation protein | STTGEYGQPMGAHQ | Q01417 [103-116] | 1463.6220 | 5.47.E+06 |
| P04347 | Glycinin G5 | LGQSQVRQLKYQGNSGPLVNP | P04347 [496-516] | 2283.2204 | 5.46.E+06 |
| P04405 | Glycinin G2 | NNPFSFLVPPQESQR | P04405 [467-481] | 1759.8762 | 5.44.E+06 |
| P04405; P04776 | Glycinin G2; Glycinin G1 | ALPEEVIQHT | P04405 [444-453]; P04776 [454-463] | 1136.5946 | 5.40.E+06 |
| P04347 | Glycinin G5 | SYNLGQSQVR | P04347 [493-502] | 1151.5804 | 5.37.E+06 |
| P29531 | P24 oleosin isoform B | TTVPPHSVQVHTTTHRYEAGVVPPAR | P29531 [2-27] | 2879.4911 | 5.32.E+06 |
| P05046 | Lectin | LPHASSNID | P05046 [264-272] | 953.4687 | 5.31.E+06 |
| P04776 | Glycinin G1 | TPMIGTLAGANSLLNALPEEVIQHTFNLKSQQARQ | P04776 [439-473] | 3790.9909 | 5.29.E+06 |
| P19594 | 2S seed storage albumin protein | LATMCRFGPMIQCDLSSDD | P19594 [140-158] | 2216.9443 | 5.28.E+06 |
| P04405; P04776 | Glycinin G2; Glycinin G1 | ALPEEVIQHTFNLKSQQ | P04405 [444-460]; P04776 [454-470] | 1982.0342 | 5.25.E+06 |
| P04776 | Glycinin G1 | TPMIGTLAGANSLLNALPEEVIQHTFNLKS | P04776 [439-468] | 3179.6769 | 5.23.E+06 |
| P04405; P04776 | Glycinin G2; Glycinin G1 | ALPEEVIQHTF | P04405 [444-454]; P04776 [454-464] | 1283.6630 | 5.04.E+06 |
| P04405; P04776 | Glycinin G2; Glycinin G1 | LAGANSLLNALPEEVIQHTFNLKSQQA | P04405 [435-461]; P04776 [445-471] | 2906.5371 | 4.92.E+06 |
| P0DO16 | Beta-conglycinin alpha subunit 1 | SYFVDAQPKKKEEGN | P0DO16 [576-590] | 1739.8599 | 4.85.E+06 |
| Q01417 | 18 kDa seed maturation protein | STTGEYGQPMGAH | Q01417 [103-115] | 1335.5634 | 4.83.E+06 |
| P69325 | Polyubiquitin | MQIFVK | P69325 [1-6]; [77-82]; [153-158]; [229-234] | 765.4328 | 4.83.E+06 |
| P04347 | Glycinin G5 | ITSSKFNECQLN | P04347 [25-36] | 1440.6788 | 4.81.E+06 |
| F7J077 | Beta-conglycinin beta subunit 2 | LKVREDENNPFYLR | F7J077 [24-37]; P25974 [24-37] | 1792.9341 | 4.79.E+06 |
| P05046 | Lectin | IPGESHDVLSWSFASNLPHASSNIDPLDLTS | P05046 [248-278] | 3306.5913 | 4.78.E+06 |
| P62163 | Calmodulin-2 | GDGQINYEEFVKVMMAK | P62163 [133-149] | 1958.9351 | 4.73.E+06 |
| P11828 | Glycinin G3 | NNPFSFLVPPKESQRR | P11828 [463-478] | 1916.0137 | 4.73.E+06 |
| P04405; P04776 | Glycinin G2; Glycinin G1 | SLENQLDQMPRRFY | P04405 [167-180]; P04776 [170-183] | 1796.8748 | 4.73.E+06 |
| P02858 | Glycinin G4 | ISSSKLNECQLNNLN | P02858 [24-38] | 1733.8487 | 4.72.E+06 |
| P26987 | Stress-induced protein SAM22 | LLAHPDYN | P26987 [151-158] | 942.4680 | 4.71.E+06 |
| P04776 | Glycinin G1 | VIKPPTD | P04776 [264-270] | 769.4454 | 4.70.E+06 |
| P04405; P04776 | Glycinin G2; Glycinin G1 | SLENQLDQMPRR | P04405 [167-178]; P04776 [170-181] | 1486.7431 | 4.68.E+06 |
| P04405 | Glycinin G2 | DRPSIGNLAGANSLLNALPEEVIQHTFNLKSQQA | P04405 [428-461] | 3645.8984 | 4.67.E+06 |
| P05046 | Lectin | SNIDPLDLTSFVLHEAI | P05046 [269-285] | 1883.9749 | 4.66.E+06 |
| P04405 | Glycinin G2 | DEEEQPQCVETDKGCQR | P04405 [277-293] | 2107.8655 | 4.65.E+06 |
| P02858 | Glycinin G4 | EQIPSHPP | P02858 [293-300] | 904.4523 | 4.63.E+06 |
| P04405 | Glycinin G2 | ALPEEVIQHTFNLKSQQARQVKNNNPFSFLVPPQESQRRA | P04405 [444-483] | 4646.4339 | 4.52.E+06 |
| P04405; P04776 | Glycinin G2; Glycinin G1 | LNALPEEVIQHTFNL | P04405 [442-456]; P04776 [452-466] | 1737.9170 | 4.48.E+06 |
| P01071; P01070 | Trypsin inhibitor B; Trypsin inhibitor A | LVVSKNKPLVVQF | P01071 [161-173]; P01070 [185-197] | 1470.9043 | 4.47.E+06 |
| P04405; P04776 | Glycinin G2; Glycinin G1 | SLLNALPEEVIQHTF | P04405 [440-454]; P04776 [450-464] | 1710.9061 | 4.44.E+06 |
| P11828 | Glycinin G3 | NPFSFLVPPKESQRR | P11828 [464-478] | 1801.9708 | 4.39.E+06 |
| P04405 | Glycinin G2 | EEEQPQCVETDKGCQRQ | P04405 [278-294] | 2120.8972 | 4.38.E+06 |
| P05046 | Lectin | LPHASSNIDPLD | P05046 [264-275] | 1278.6325 | 4.37.E+06 |
| P09186 | Seed linoleate 9S-lipoxygenase-3 | MLGGLLHR | P09186 [1-8] | 938.5240 | 4.28.E+06 |
| F7J077 | Beta-conglycinin beta subunit 2 | LKVREDENNPFY | F7J077 [24-35]; P25974 [24-35] | 1523.7489 | 4.27.E+06 |
| P05046 | Lectin | VLHEAI | P05046 [280-285] | 681.3930 | 4.26.E+06 |
| P04405 | Glycinin G2 | GELQEGGVLIVPQNF | P04405 [394-408] | 1599.8377 | 4.25.E+06 |
| P02858 | Glycinin G4 | GPLVNPESQQGSPR | P02858 [546-559] | 1465.7394 | 4.17.E+06 |
| P04347 | Glycinin G5 | KYQGNSGPLVNP | P04347 [505-516] | 1273.6535 | 4.12.E+06 |
| P09186 | Seed linoleate 9S-lipoxygenase-3 | LPSSKEGLTFRGIPNSISI | P09186 [839-857] | 2016.1124 | 4.11.E+06 |
| Q01417 | 18 kDa seed maturation protein | NTRAGGKPNDYGYGTGGT | Q01417 [156-173] | 1785.8151 | 4.07.E+06 |
| P04776 | Glycinin G1 | SVIKPPTD | P04776 [263-270] | 856.4775 | 4.07.E+06 |
| P04405; P04776 | Glycinin G2; Glycinin G1 | SLLNALPEEVIQH | P04405 [440-452]; P04776 [450-462] | 1462.7900 | 4.04.E+06 |
| P09186; P08170; P09439 | Seed linoleate 9S-lipoxygenase-3; Seed linoleate 13S-lipoxygenase-1; Seed linoleate 9S-lipoxygenase-2 | RGIPNSISI | P09186 [849-857]; P08170 [831-839]; P38417 [845-853]; P09439 [857-865] | 956.5524 | 4.03.E+06 |
| P29531 | P24 oleosin isoform B | TTVPPHSVQVHTTT | P29531 [2-15] | 1546.7860 | 4.02.E+06 |
| P05046 | Lectin | FVLHEA | P05046 [279-284] | 715.3774 | 4.01.E+06 |
| F7J077 | Beta-conglycinin beta subunit 2 | LKVREDENNPFYLRSSNSFQTLFENQN | F7J077 [24-50]; P25974 [24-50] | 3289.5872 | 3.91.E+06 |
| P13917 | Basic 7S globulin | VTPTKPINL | P13917 [25-33] | 982.5932 | 3.90.E+06 |
| Q01417 | 18 kDa seed maturation protein | MQGGKKAGESIKETATNIGA | Q01417 [1-20] | 2033.0332 | 3.88.E+06 |
| P04776 | Glycinin G1 | FSSREQPQQNECQIQK | P04776 [20-35] | 2006.9349 | 3.86.E+06 |
| P04405; P04776 | Glycinin G2; Glycinin G1 | SLLNALPEEVIQHTFNLKSQQAR | P04405 [440-462]; P04776 [450-472] | 2636.4155 | 3.81.E+06 |
| P04405; P04776 | Glycinin G2; Glycinin G1 | TPVVAVSIIDTNSLENQLDQMPRRF | P04405 [155-179]; P04776 [158-182] | 2843.4720 | 3.81.E+06 |
| P04776 | Glycinin G1 | PFKFLVPPQESQKRAV | P04776 [479-494] | 1871.0538 | 3.79.E+06 |
| P04405; P04776 | Glycinin G2; Glycinin G1 | SLENQLDQMPRRFYLAGN | P04405 [167-184]; P04776 [170-187] | 2152.0604 | 3.78.E+06 |
| P09186 | Seed linoleate 9S-lipoxygenase-3 | IEDPSCPHGIR | P09186 [611-621] | 1280.6052 | 3.71.E+06 |
| P11828 | Glycinin G3 | LNALPEEVIQQTFNL | P11828 [438-452] | 1728.9167 | 3.69.E+06 |
| P05046 | Lectin | PPNPHIGIN | P05046 [166-174] | 958.5105 | 3.69.E+06 |
| P29531 | P24 oleosin isoform B | TTVPPHSVQVHTTTHRY | P29531 [2-18] | 2003.0094 | 3.69.E+06 |
| F7J077 | Beta-conglycinin beta subunit 2 | LKVREDENNPFYLRS | F7J077 [24-38]; P25974 [24-38] | 1879.9661 | 3.66.E+06 |
| P04776 | Glycinin G1 | FSSREQPQQNECQIQKLNALKPDN | P04776 [20-43] | 2872.4006 | 3.60.E+06 |
| P05046 | Lectin | SNLPHASSNIDPLDLTSFVLHEAI | P05046 [262-285] | 2590.3148 | 3.60.E+06 |
| P04347 | Glycinin G5 | SYNLGQSQVRQLK | P04347 [493-505] | 1520.8180 | 3.59.E+06 |
| P13917 | Basic 7S globulin | VTPTKPINLVVLPVQNDG | P13917 [25-42] | 1904.0851 | 3.56.E+06 |
| P11828 | Glycinin G3 | PFSFLVPPKESQR | P11828 [465-477] | 1531.8267 | 3.56.E+06 |
| P04776 | Glycinin G1 | DTPMIGTLAGANSLLNALPEEVIQHTFN | P04776 [438-465] | 2966.4928 | 3.51.E+06 |
| P04405; P04776 | Glycinin G2; Glycinin G1 | YLAGNQEQEFLK | P04405 [180-191]; P04776 [183-194] | 1439.7165 | 3.50.E+06 |
| P02858 | Glycinin G4 | ISSSKLNECQLNN | P02858 [24-36] | 1506.7217 | 3.47.E+06 |
| P08170 | Seed linoleate 13S-lipoxygenase-1 | MFSAGHKIKG | P08170 [1-10] | 1117.5823 | 3.46.E+06 |
| P11827; F7J077; P0DO16 | Beta-conglycinin alpha' subunit; Beta-conglycinin beta subunit 2; Beta-conglycinin alpha subunit 1 | INAENNQRNF | P11827 [545-554]; F7J077 [363-372]; P25974 [363-372]; P0DO16 [529-538] | 1219.5814 | 3.39.E+06 |
| P11828; P04405 | Glycinin G3; Glycinin G2 | DRPSIGNLAGAN | P11828 [424-435]; P04405 [428-439] | 1184.6018 | 3.33.E+06 |
| P01070 | Trypsin inhibitor A | VFCPQQAEDDKCG | P01070 [158-170] | 1553.6359 | 3.32.E+06 |
| P04776 | Glycinin G1 | IKNNNPFKFLVPPQESQKR | P04776 [474-492] | 2284.2561 | 3.29.E+06 |
| P11828 | Glycinin G3 | SFQNQLDQMPRRF | P11828 [167-179] | 1666.8118 | 3.29.E+06 |
| P04405 | Glycinin G2 | DDDEEEQPQCVETDKGCQRQS | P04405 [275-295] | 2553.0100 | 3.28.E+06 |
| P04776 | Glycinin G1 | GGLSVIKPPTD | P04776 [260-270] | 1083.6045 | 3.24.E+06 |
| P19594 | 2S seed storage albumin protein | MCRFGPMIQCDLSSDD | P19594 [143-158] | 1931.7755 | 3.23.E+06 |
| P08170 | Seed linoleate 13S-lipoxygenase-1 | MFSAGHKIKGTVVL | P08170 [1-14] | 1529.8509 | 3.19.E+06 |
| P04776 | Glycinin G1 | PPQESQKR | P04776 [485-492] | 969.5112 | 3.18.E+06 |
| P05046 | Lectin | IPGESHDVLSWSFASNLPHASSNIDPLD | P05046 [248-275] | 3005.4276 | 3.17.E+06 |
| P08170 | Seed linoleate 13S-lipoxygenase-1 | AFLGRSVSL | P08170 [34-42] | 949.5465 | 3.17.E+06 |
| P01071; P01070 | Trypsin inhibitor B; Trypsin inhibitor A | LPEGPAVKIGENKD | P01071 [99-112]; P01070 [123-136] | 1466.7849 | 3.15.E+06 |
| P08170 | Seed linoleate 13S-lipoxygenase-1 | MFSAGHKIKGTVVLMPKNELEVNPDGSA | P08170 [1-28] | 3011.5329 | 3.11.E+06 |
| Q01417 | 18 kDa seed maturation protein | MQGGKKAGESIK | Q01417 [1-12] | 1275.6726 | 3.08.E+06 |
| P29530 | P24 oleosin isoform A | TTQVPPHSVQVHTTT | P29530 [2-16] | 1674.8446 | 3.04.E+06 |
| P04405 | Glycinin G2 | EQPQCVETDKGCQRQS | P04405 [280-295] | 1949.8440 | 3.04.E+06 |
| P11828; P04776 | Glycinin G3; Glycinin G1 | GIDETICTMRLRHNIG | P11828 [297-312]; P04776 [311-326] | 1885.9371 | 3.03.E+06 |
| P09439 | Seed linoleate 9S-lipoxygenase-2 | MFSVPGVSGILN | P09439 [1-12] | 1262.6449 | 3.01.E+06 |
| P04347 | Glycinin G5 | VRQLKYQGNSGPLVNP | P04347 [501-516] | 1769.9657 | 2.98.E+06 |
| Q01417 | 18 kDa seed maturation protein | TSAMPGHGTGQPTGHVTEGVVGSHPIGTNRGPGGTATAHN | Q01417 [117-156] | 3802.8063 | 2.97.E+06 |
| P04405 | Glycinin G2 | LKEAFGVNMQ | P04405 [226-235] | 1136.5769 | 2.96.E+06 |
| P08170 | Seed linoleate 13S-lipoxygenase-1 | MPKNELEVN | P08170 [15-23] | 1073.5296 | 2.96.E+06 |
| P05046 | Lectin | SSNIDPLDLTSFVLHEA | P05046 [268-284] | 1857.9229 | 2.94.E+06 |
| P04776 | Glycinin G1 | GIDETICTMRLRHNIGQTSSPDIYNPQAG | P04776 [311-339] | 3244.5474 | 2.94.E+06 |
| P04776 | Glycinin G1 | PFKFLVPPQESQKR | P04776 [479-492] | 1742.9588 | 2.92.E+06 |
| P11828; P04776 | Glycinin G3; Glycinin G1 | GIDETICTMRLRHN | P11828 [297-310]; P04776 [311-324] | 1715.8316 | 2.91.E+06 |
| Q01417 | 18 kDa seed maturation protein | MQGGKKAGESI | Q01417 [1-11] | 1147.5776 | 2.90.E+06 |
| P04405 | Glycinin G2 | GIDETICTMRLRQN | P04405 [301-314] | 1706.8313 | 2.88.E+06 |
| P02858 | Glycinin G4 | GVEENICTLKLHENIARPS | P02858 [379-397] | 2180.1128 | 2.85.E+06 |
| P13917 | Basic 7S globulin | VTPTKPINLVVLPV | P13917 [25-38] | 1489.9352 | 2.84.E+06 |
| P08170 | Seed linoleate 13S-lipoxygenase-1 | MFSAGHKIK | P08170 [1-9] | 1060.5608 | 2.83.E+06 |
| P02858 | Glycinin G4 | GVEENICTLKLHENIARP | P02858 [379-396] | 2093.0808 | 2.83.E+06 |
| P04776 | Glycinin G1 | PFKFLVPPQESQ | P04776 [479-490] | 1416.7522 | 2.82.E+06 |
| P04405; P04776 | Glycinin G2; Glycinin G1 | LAGANSLLNALPEEVIQHTFNLKSQQ | P04405 [435-460]; P04776 [445-470] | 2835.4999 | 2.82.E+06 |
| P01055 | Bowman-Birk type proteinase inhibitor | FCYEPCKPSEDDKEN | P01055 [96-110] | 1917.7630 | 2.82.E+06 |
| P04405 | Glycinin G2 | DRPSIGNLAGANSLLNALPEEVIQHTFNLKSQQARQ | P04405 [428-463] | 3930.0580 | 2.80.E+06 |
| P11828; P04405; P04776 | Glycinin G3; Glycinin G2; Glycinin G1 | GIDETICTMR | P11828 [297-306]; P04405 [301-310]; P04776 [311-320] | 1195.5446 | 2.80.E+06 |
| P05046 | Lectin | AETVSFSWNK | P05046 [33-42] | 1168.5633 | 2.76.E+06 |
| P04776 | Glycinin G1 | TPMIGTLAGANSLLNALPEEVIQHTFN | P04776 [439-465] | 2851.4659 | 2.74.E+06 |
| P13917 | Basic 7S globulin | VTPTKPINLVVLPVQND | P13917 [25-41] | 1847.0637 | 2.74.E+06 |
| P02858 | Glycinin G4 | PLVNPESQQGSP | P02858 [547-558] | 1252.6168 | 2.71.E+06 |
| P04405 | Glycinin G2 | GIDETICTMRLRQNIGQNSSPDIYNPQAG | P04405 [301-329] | 3248.5423 | 2.70.E+06 |
| P11827 | Beta-conglycinin alpha' subunit | SESYFVDAQPQQKEEGN | P11827 [590-606] | 1955.8617 | 2.67.E+06 |
| P11828 | Glycinin G3 | SLLNALPEEVIQQTFNLRRQQA | P11828 [436-457] | 2568.3893 | 2.67.E+06 |
| P02858 | Glycinin G4 | LDQTPRVFY | P02858 [180-188] | 1138.5891 | 2.65.E+06 |
| P04405 | Glycinin G2 | ILSGFAPEFLKEAFGVN | P04405 [217-233] | 1838.9687 | 2.64.E+06 |
| P04405; P04776 | Glycinin G2; Glycinin G1 | SLLNALPEEVIQHT | P04405 [440-453]; P04776 [450-463] | 1563.8377 | 2.63.E+06 |
| P11828 | Glycinin G3 | SLLNALPEEVIQQTFNLRR | P11828 [436-454] | 2241.2350 | 2.63.E+06 |
| Q8RVH5 | Basic 7S globulin 2 | HHTNPTKPINLLVLPVQN | Q8RVH5 [30-47] | 2035.1447 | 2.60.E+06 |
| P25272 | Kunitz-type trypsin inhibitor KTI1 | KCEDIGIQIDDDGIRRL | P25272 [167-183] | 2016.0179 | 2.58.E+06 |
| Q01417 | 18 kDa seed maturation protein | SAMPGHGTGQPTGHVT | Q01417 [118-133] | 1534.7067 | 2.53.E+06 |
| P04776 | Glycinin G1 | DTPMIGTLAGANSL | P04776 [438-451] | 1360.6777 | 2.52.E+06 |
| P04405 | Glycinin G2 | RPSIGNLAGANSLLNALPEEVIQHTFNLKS | P04405 [429-458] | 3203.7171 | 2.51.E+06 |
| P01064; P01063 | Bowman-Birk type proteinase inhibitor D-II; Bowman-Birk type proteinase inhibitor C-II | DFCYKPC | P01064 [72-78]; P01063 [69-75] | 989.3856 | 2.50.E+06 |
| P02858 | Glycinin G4 | TYNTGDEPVVAISLLDTSNFNNQLDQTPRVFY | P02858 [157-188] | 3631.7551 | 2.48.E+06 |
| P09186 | Seed linoleate 9S-lipoxygenase-3 | SKEGLTFRGIPNSISI | P09186 [842-857] | 1718.9436 | 2.47.E+06 |
| P11828 | Glycinin G3 | ALPEEVIQQTFNLRRQQA | P11828 [440-457] | 2141.1462 | 2.45.E+06 |
| Q01417 | 18 kDa seed maturation protein | STTGEYGQPMGAHQT | Q01417 [103-117] | 1564.6697 | 2.44.E+06 |
| P26413 | Heat shock 70 kDa protein | GKELCKSINPDEAVAYGAAVQA | P26413 [361-382] | 2291.1336 | 2.44.E+06 |
| P04776 | Glycinin G1 | PFKFLVPPQESQKRAVA | P04776 [479-495] | 1942.0909 | 2.42.E+06 |
| P04776 | Glycinin G1 | FSSREQPQQN | P04776 [20-29] | 1220.5654 | 2.41.E+06 |
| P04405 | Glycinin G2 | EEEQPQCVETDKGCQR | P04405 [278-293] | 1992.8386 | 2.41.E+06 |
| P11827 | Beta-conglycinin alpha' subunit | SYFVDAQPQQKEEGN | P11827 [592-606] | 1739.7871 | 2.41.E+06 |
| Q01417 | 18 kDa seed maturation protein | SAMPGHGTGQPTGHVTEGV | Q01417 [118-136] | 1819.8392 | 2.40.E+06 |
| P05046 | Lectin | SSNIDPLDLTS | P05046 [268-278] | 1161.5634 | 2.40.E+06 |
| P05046 | Lectin | LPHASSNIDPLDLTSFVLHEA | P05046 [264-284] | 2276.1557 | 2.39.E+06 |
| P11828 | Glycinin G3 | ALPEEVIQQTFNLRR | P11828 [440-454] | 1813.9919 | 2.37.E+06 |
| P13917 | Basic 7S globulin | VTPTKPINLVVLPVQNDGSTGLH | P13917 [25-47] | 2399.3293 | 2.36.E+06 |
| P04405 | Glycinin G2 | DRPSIGNLAGANSLLNALPEEVIQHTFNLK | P04405 [428-457] | 3231.7121 | 2.36.E+06 |
| P05046 | Lectin | IDPLDLTS | P05046 [271-278] | 873.4564 | 2.34.E+06 |
| P29530; P29531 | P24 oleosin isoform A; P24 oleosin isoform B | TTVTATTATA | P29530 [217-226]; P29531 [214-223] | 937.4837 | 2.34.E+06 |
| P04776 | Glycinin G1 | EGEDKGAIVTVKGGLSVIKPPTD | P04776 [248-270] | 2310.2551 | 2.33.E+06 |
| P22895 | P34 probable thiol protease | YFASYPTKEESETLV | P22895 [350-364] | 1763.8374 | 2.33.E+06 |
| P11827 | Beta-conglycinin alpha' subunit | EGEQPRPFP | P11827 [96-104] | 1056.5109 | 2.30.E+06 |
| P04405 | Glycinin G2 | DDEEEQPQCVETDKGCQRQ | P04405 [276-294] | 2350.9510 | 2.30.E+06 |
| P04405 | Glycinin G2 | DRPSIGNLAGANSLLNALPEEVIQHTFNLKSQQ | P04405 [428-460] | 3574.8612 | 2.29.E+06 |
| P29530 | P24 oleosin isoform A | TTQVPPHS | P29530 [2-9] | 908.4472 | 2.28.E+06 |
| P01071; P01070 | Trypsin inhibitor B; Trypsin inhibitor A | SDITAFGGIRAAPTGN | P01071 [21-36]; P01070 [45-60] | 1547.7813 | 2.28.E+06 |
| P22895 | P34 probable thiol protease | FASYPTKEESETL | P22895 [351-363] | 1501.7057 | 2.27.E+06 |
| P04776 | Glycinin G1 | FKFLVPPQESQKRA | P04776 [480-493] | 1674.9326 | 2.27.E+06 |
| P04347 | Glycinin G5 | ITSSKFNECQLNNLN | P04347 [25-39] | 1781.8487 | 2.23.E+06 |
| P02858 | Glycinin G4 | GVEENICTLKLHE | P02858 [379-391] | 1541.7628 | 2.21.E+06 |
| P02858 | Glycinin G4 | FNNQLDQTPRVF | P02858 [176-187] | 1478.7387 | 2.20.E+06 |
| P04347 | Glycinin G5 | GVEENICTMKLHEN | P04347 [345-358] | 1673.7622 | 2.19.E+06 |
| P04405 | Glycinin G2 | RPSIGNLAGANSLLNALPEEVIQHTFNLK | P04405 [429-457] | 3116.6851 | 2.15.E+06 |
| P04776 | Glycinin G1 | NPFKFLVPPQESQKRAV | P04776 [478-494] | 1985.0967 | 2.15.E+06 |
| P11827 | Beta-conglycinin alpha' subunit | IENLIKSQSESYFVDAQPQQKEEGN | P11827 [582-606] | 2881.3850 | 2.15.E+06 |
| P04405 | Glycinin G2 | LREQAQQNECQIQ | P04405 [19-31] | 1644.7758 | 2.14.E+06 |
| P08170 | Seed linoleate 13S-lipoxygenase-1 | SEEGLTFRGIPNSISI | P08170 [824-839]; P38417 [838-853] | 1719.8912 | 2.14.E+06 |
| P11828 | Glycinin G3 | NNPFSFLVPPKESQRRV | P11828 [463-479] | 2015.0821 | 2.13.E+06 |
| P22895 | P34 probable thiol protease | SYPTKEESETL | P22895 [353-363] | 1283.6002 | 2.12.E+06 |
| P49045 | Vacuolar-processing enzyme | TYCPGMDPSPPPEYITC | P49045 [251-267] | 1984.8126 | 2.11.E+06 |
| P02858; P04347 | Glycinin G4; Glycinin G5 | TYNTGDEPVVA | P02858 [157-167]; P04347 [157-167] | 1165.5372 | 2.10.E+06 |
| P01070 | Trypsin inhibitor A | DFVLDNEGNPLENGGTYYIL | P01070 [25-44] | 2243.0503 | 2.08.E+06 |
| P04776 | Glycinin G1 | PPQESQKRA | P04776 [485-493] | 1040.5483 | 2.07.E+06 |
| Q01417 | 18 kDa seed maturation protein | TGEYGQPMGAHQT | Q01417 [105-117] | 1376.5899 | 2.05.E+06 |
| P10538 | Beta-amylase | WELPDDAGKY | P10538 [230-239] | 1193.5473 | 2.05.E+06 |
| P01071; P01070 | Trypsin inhibitor B; Trypsin inhibitor A | LPEGPAVKIGEN | P01071 [99-110]; P01070 [123-134] | 1223.6630 | 2.04.E+06 |
| P04347 | Glycinin G5 | PDIEHPETMQQQQQQK | P04347 [193-208] | 1964.9131 | 2.04.E+06 |
| P04405 | Glycinin G2 | GIDETICTMRLRQNIGQ | P04405 [301-317] | 2004.9954 | 2.03.E+06 |
| P49045 | Vacuolar-processing enzyme | TYCPGMDPSPPP | P49045 [251-262] | 1318.5442 | 2.02.E+06 |
| P04776 | Glycinin G1 | RHNIGQTSSPDIYN | P04776 [322-335] | 1601.7667 | 1.99.E+06 |
| P04405 | Glycinin G2 | LKEAFGVNMQI | P04405 [226-236] | 1249.6609 | 1.96.E+06 |
| P08170 | Seed linoleate 13S-lipoxygenase-1 | VVLMPKNELEVN | P08170 [12-23] | 1384.7505 | 1.94.E+06 |
| P29531 | P24 oleosin isoform B | TTVPPH | P29531 [2-7] | 693.3566 | 1.93.E+06 |
| P02858 | Glycinin G4 | GVEENICTLKLHENIARPSRADFYNPKAGRISTLN | P02858 [379-413] | 3984.0508 | 1.91.E+06 |
| P01064 | Bowman-Birk type proteinase inhibitor D-II | YSKPCCDL | P01064 [20-27] | 1042.4332 | 1.88.E+06 |
| P09186; P08170 | Seed linoleate 9S-lipoxygenase-3; Seed linoleate 13S-lipoxygenase-1 | TFRGIPNSISI | P09186 [847-857]; P08170 [829-839]; P38417 [843-853] | 1204.6685 | 1.88.E+06 |
| P04405 | Glycinin G2 | PFSFLVPPQESQRRAVA | P04405 [469-485] | 1929.0341 | 1.86.E+06 |
| P0DO16 | Beta-conglycinin alpha subunit 1 | VLQRFNQRSPQLQ | P0DO16 [216-228] | 1613.8871 | 1.84.E+06 |
| P08170 | Seed linoleate 13S-lipoxygenase-1 | MFSAGHKIKGTVVLMPKNELEVN | P08170 [1-23] | 2600.3575 | 1.84.E+06 |
| P04405 | Glycinin G2 | EQPQCVETDKGCQR | P04405 [280-293] | 1734.7534 | 1.84.E+06 |
| P49045 | Vacuolar-processing enzyme | TYCPGMDPSP | P49045 [251-260] | 1124.4387 | 1.83.E+06 |
| Q39846 | Seed biotin-containing protein SBP65 | ASEQLARRENTTTEKEIH | Q39846 [2-19] | 2155.0738 | 1.83.E+06 |
| P11827 | Beta-conglycinin alpha' subunit | FVDAQPQQKEEGN | P11827 [594-606] | 1489.6918 | 1.83.E+06 |
| P11827 | Beta-conglycinin alpha' subunit | LIKSQSESYFVDAQPQQKEEGN | P11827 [585-606] | 2525.2154 | 1.79.E+06 |
| P01070 | Trypsin inhibitor A | DFVLDNEGNPLENG | P01070 [25-38] | 1532.6863 | 1.76.E+06 |
| P04405 | Glycinin G2 | PFSFLVPPQESQ | P04405 [469-480] | 1375.6892 | 1.74.E+06 |
| P04405; P04776 | Glycinin G2; Glycinin G1 | GANSLLNALPEEVIQHTFNLK | P04405 [437-457]; P04776 [447-467] | 2308.2296 | 1.74.E+06 |
| P04347 | Glycinin G5 | SQVRQLKYQGNSGPLVNP | P04347 [499-516] | 1985.0563 | 1.74.E+06 |
| P11828; P04776 | Glycinin G3; Glycinin G1 | GIDETICTMRLRHNIGQ | P11828 [297-313]; P04776 [311-327] | 2013.9957 | 1.73.E+06 |
| P04405 | Glycinin G2 | DEEEQPQCVETDKGCQRQSK | P04405 [277-296] | 2451.0511 | 1.72.E+06 |
| P01071; P01070 | Trypsin inhibitor B; Trypsin inhibitor A | IGISIDHDDGTRR | P01071 [148-160]; P01070 [172-184] | 1454.7346 | 1.72.E+06 |
| P04405 | Glycinin G2 | GERVFDGELQEGGVLIVPQNF | P04405 [388-408] | 2303.1666 | 1.67.E+06 |
| P08170 | Seed linoleate 13S-lipoxygenase-1 | PSLQGNRLGPVQLP | P08170 [803-816] | 1475.8329 | 1.66.E+06 |
| P04405 | Glycinin G2 | DDEEEQPQCVETDKGCQR | P04405 [276-293] | 2222.8925 | 1.64.E+06 |
| Q01417 | 18 kDa seed maturation protein | GKPNDYGYGTGGT | Q01417 [161-173] | 1286.5648 | 1.64.E+06 |
| P26413 | Heat shock 70 kDa protein | KSINPDEAVAYGAAVQAA | P26413 [366-383] | 1774.8970 | 1.64.E+06 |
| P04347 | Glycinin G5 | PDIEHPETMQQQQQ | P04347 [193-206] | 1708.7595 | 1.64.E+06 |
| Q8RVH5 | Basic 7S globulin 2 | TVTPQGEYNVRVS | Q8RVH5 [254-266] | 1449.7332 | 1.63.E+06 |
| P04776 | Glycinin G1 | ALPEEVIQHTFNLKSQQARQIKN | P04776 [454-476] | 2692.4529 | 1.62.E+06 |
| P02858 | Glycinin G4 | WGPLVNPESQQGS | P02858 [545-557] | 1398.6648 | 1.61.E+06 |
| P02858 | Glycinin G4 | YLAGNPDIEYPETMQQQQQ | P02858 [188-206] | 2253.0128 | 1.59.E+06 |
| Q8RVH5 | Basic 7S globulin 2 | SIRINQHSVFPPN | Q8RVH5 [267-279] | 1508.7968 | 1.59.E+06 |
| P04347 | Glycinin G5 | GVEENICTMKLH | P04347 [345-356] | 1430.6767 | 1.57.E+06 |
| P02858 | Glycinin G4 | GVEENICTLK | P02858 [379-388] | 1162.5773 | 1.56.E+06 |
| P05046 | Lectin | LPHASSNIDPLDL | P05046 [264-276] | 1391.7165 | 1.56.E+06 |
| P04405 | Glycinin G2 | FAPEFLKEAFGVN | P04405 [221-233] | 1468.7471 | 1.56.E+06 |
| P13917 | Basic 7S globulin | VTPTKPINLVVLPVQNDGSTGLHWANLQK | P13917 [25-53] | 3139.7262 | 1.55.E+06 |
| P04405; P04776 | Glycinin G2; Glycinin G1 | ANSLLNALPEEVIQHTFNLKS | P04405 [438-458]; P04776 [448-468] | 2338.2401 | 1.55.E+06 |
| P02858 | Glycinin G4 | IIIAQGKGAL | P02858 [92-101] | 983.6248 | 1.55.E+06 |
| Q8RVH5 | Basic 7S globulin 2 | VPIPQHHTNPTKPINLLVLPVQNDASTGLHWAN | Q8RVH5 [25-57] | 3621.9289 | 1.54.E+06 |
| P04405 | Glycinin G2 | GIDETICTMRLRQNIGQN | P04405 [301-318] | 2135.0332 | 1.54.E+06 |
| P11827 | Beta-conglycinin alpha' subunit | YFVDAQPQQKEEGN | P11827 [593-606] | 1652.7551 | 1.53.E+06 |
| P04776 | Glycinin G1 | SLLNALPEEVIQHTFNLKSQQARQIKN | P04776 [450-476] | 3119.6960 | 1.53.E+06 |
| P05046 | Lectin | LDLTSFVLHEAI | P05046 [274-285] | 1357.7362 | 1.53.E+06 |
| P04347 | Glycinin G5 | FNNQLDQNPRVF | P04347 [176-187] | 1491.7339 | 1.51.E+06 |
| P08170 | Seed linoleate 13S-lipoxygenase-1 | RLGPVQLPYTLLYPSSEEGLT | P08170 [809-829] | 2333.2387 | 1.51.E+06 |
| P02858 | Glycinin G4 | YLAGNPDIEYPETMQQQQQQK | P02858 [188-208] | 2509.1664 | 1.50.E+06 |
| P08170 | Seed linoleate 13S-lipoxygenase-1 | AGHKIKGTVVLMPKNELEVN | P08170 [4-23] | 2177.2111 | 1.49.E+06 |
| P04405 | Glycinin G2 | SNILSGFAPEFLKE | P04405 [215-228] | 1551.8053 | 1.49.E+06 |
| P11828 | Glycinin G3 | SLLNALPEEVIQQTFNL | P11828 [436-452] | 1929.0328 | 1.48.E+06 |
| P11828 | Glycinin G3 | FSFREQPQQ | P11828 [20-28] | 1166.5589 | 1.48.E+06 |
| P01070 | Trypsin inhibitor A | DFVLDNEGNPLENGG | P01070 [25-39] | 1589.7078 | 1.48.E+06 |
| P04405 | Glycinin G2 | GPQEIYIQQGN | P04405 [84-94] | 1246.6062 | 1.47.E+06 |
| P08170 | Seed linoleate 13S-lipoxygenase-1 | MFSAGHKIKGT | P08170 [1-11] | 1218.6300 | 1.47.E+06 |
| P04776 | Glycinin G1 | DTPMIGTLAGAN | P04776 [438-449] | 1160.5616 | 1.46.E+06 |
| P04405 | Glycinin G2 | EQPQCVETDKGCQRQ | P04405 [280-294] | 1862.8120 | 1.46.E+06 |
| P04405 | Glycinin G2 | SQQARQVKNNNPFSFLVPPQESQRR | P04405 [458-482] | 2955.5296 | 1.45.E+06 |
| P22895 | P34 probable thiol protease | FASYPTKEESET | P22895 [351-362] | 1388.6216 | 1.45.E+06 |
| P0DO16 | Beta-conglycinin alpha subunit 1 | EDEQPRPIP | P0DO16 [97-105] | 1080.5320 | 1.45.E+06 |
| P04405; P04776 | Glycinin G2; Glycinin G1 | LLNALPEEVIQHTFN | P04405 [441-455]; P04776 [451-465] | 1737.9170 | 1.43.E+06 |
| P04405; P04776 | Glycinin G2; Glycinin G1 | LLNALPEEVIQHTFNLKSQQARQ | P04405 [441-463]; P04776 [451-473] | 2677.4420 | 1.43.E+06 |
| P04347 | Glycinin G5 | ITSSKFN | P04347 [25-31] | 796.4199 | 1.43.E+06 |
| Q8RVH5 | Basic 7S globulin 2 | PTKPINLLVLPVQN | Q8RVH5 [34-47] | 1545.9363 | 1.43.E+06 |
| P05046 | Lectin | IPGESHDVLSWSFASNLPHASSNIDPLDL | P05046 [248-276] | 3118.5116 | 1.41.E+06 |
| P49045 | Vacuolar-processing enzyme | YAMGSHVMQ | P49045 [307-315] | 1023.4387 | 1.41.E+06 |
| P08170 | Seed linoleate 13S-lipoxygenase-1 | YPSSEEGLTFRGIPNSISI | P08170 [821-839]; P38417 [835-853] | 2067.0393 | 1.41.E+06 |
| P0DO16 | Beta-conglycinin alpha subunit 1 | FLAGSQDNVISQIPSQVQELAFPGSAQAVEKLLKNQRESY | P0DO16 [538-577] | 4390.2678 | 1.40.E+06 |
| P04347 | Glycinin G5 | FNNQLDQNPRVFY | P04347 [176-188] | 1654.7972 | 1.39.E+06 |
| P04776 | Glycinin G1 | DTPMIGTLAGANSLLNALPEEVIQHTFNLKSQQARQIK | P04776 [438-475] | 4147.1968 | 1.39.E+06 |
| P04405 | Glycinin G2 | FLVPPQESQR | P04405 [472-481] | 1200.6372 | 1.39.E+06 |
| P04776 | Glycinin G1 | GIDETICTMRLRHNIGQTSSPDIYN | P04776 [311-335] | 2907.3724 | 1.39.E+06 |
| P19594 | 2S seed storage albumin protein | LINLATMCRFGPMIQCDLSSDD | P19594 [137-158] | 2557.1554 | 1.38.E+06 |
| P02858 | Glycinin G4 | ISLLDTSNFNNQLDQTPRVFYL | P02858 [168-189] | 2598.3198 | 1.37.E+06 |
| P02858 | Glycinin G4 | EDEQIPSHPP | P02858 [291-300] | 1148.5218 | 1.37.E+06 |
| P04405 | Glycinin G2 | LREQAQQNECQIQK | P04405 [19-32] | 1772.8708 | 1.36.E+06 |
| P15627 | Capsid protein | ITINEELKNLSS | P15627 [134-145] | 1402.7424 | 1.36.E+06 |
| P04405 | Glycinin G2 | SLLNALPEEVIQHTFNLKSQQARQVKNNNPFSFLVPPQESQR | P04405 [440-481] | 4846.5387 | 1.36.E+06 |
| P04405 | Glycinin G2 | VKNNNPFSFLVPPQESQR | P04405 [464-481] | 2101.0825 | 1.36.E+06 |
| P26413 | Heat shock 70 kDa protein | GKELCKSINPDEAVAYGAAVQAA | P26413 [361-383] | 2362.1707 | 1.36.E+06 |
| P02858 | Glycinin G4 | ISLLDTSNFNNQLDQTPRVFY | P02858 [168-188] | 2485.2358 | 1.36.E+06 |
| P62163 | Calmodulin-2 | VDGDGQINYEEFVKVMMAK | P62163 [131-149] | 2173.0304 | 1.36.E+06 |
| P04405; P04776 | Glycinin G2; Glycinin G1 | AGANSLLNALPEEVIQHTFNLK | P04405 [436-457]; P04776 [446-467] | 2379.2667 | 1.35.E+06 |
| P02858 | Glycinin G4 | FNNQLDQTPRVFY | P02858 [176-188] | 1641.8020 | 1.34.E+06 |
| Q8RVH5 | Basic 7S globulin 2 | SIRINQHSVFPP | Q8RVH5 [267-278] | 1394.7539 | 1.34.E+06 |
| P04347 | Glycinin G5 | PDIEHPETMQQQQQQKS | P04347 [193-209] | 2051.9451 | 1.34.E+06 |
| Q01417 | 18 kDa seed maturation protein | MQGGKKAGESIKETATNIG | Q01417 [1-19] | 1961.9961 | 1.34.E+06 |
| P02858; P04347 | Glycinin G4; Glycinin G5 | SVLSGFSKHFL | P02858 [228-238]; P04347 [227-237] | 1221.6626 | 1.33.E+06 |
| P02858 | Glycinin G4 | DEDEQIPSHPP | P02858 [290-300] | 1263.5488 | 1.33.E+06 |
| P11827 | Beta-conglycinin alpha' subunit | IENLIK | P11827 [582-587] | 729.4505 | 1.33.E+06 |
| P02858 | Glycinin G4 | GPLVNPESQQGSP | P02858 [546-558] | 1309.6383 | 1.33.E+06 |
| P09186 | Seed linoleate 9S-lipoxygenase-3 | PSSKEGLTFRGIPNSISI | P09186 [840-857] | 1903.0284 | 1.32.E+06 |
| P25272 | Kunitz-type trypsin inhibitor KTI1 | KCEDIGIQIDDDGIRRLVL | P25272 [167-185] | 2228.1703 | 1.32.E+06 |
| P04347 | Glycinin G5 | GVEENICTMKLHENIARPSRADFYNPKAGRISTLN | P04347 [345-379] | 4002.0073 | 1.32.E+06 |
| P11827 | Beta-conglycinin alpha' subunit | IENLIKSQ | P11827 [582-589] | 944.5411 | 1.31.E+06 |
| P02858 | Glycinin G4 | GIYSPHWNLNAN | P02858 [436-447] | 1385.6597 | 1.30.E+06 |
| I1N2Z5 | Protein SLE1 | MESQQANREELDEKARQGETVVPGGTGGKS | I1N2Z5 [1-30] | 3230.5342 | 1.30.E+06 |
| P04405; P04776 | Glycinin G2; Glycinin G1 | AGANSLLNALPEEVIQHTFNLKS | P04405 [436-458]; P04776 [446-468] | 2466.2987 | 1.29.E+06 |
| Q01417 | 18 kDa seed maturation protein | GGKPNDYGYGTGGT | Q01417 [160-173] | 1343.5862 | 1.29.E+06 |
| P01064 | Bowman-Birk type proteinase inhibitor D-II | RSMPPQCSCEDIR | P01064 [32-44] | 1635.7036 | 1.28.E+06 |
| P02858 | Glycinin G4 | LKYEGNWGPLVNPESQQG | P02858 [539-556] | 2015.9821 | 1.26.E+06 |
| P04405 | Glycinin G2 | NNNPFSFLVPPQESQRR | P04405 [466-482] | 2030.0203 | 1.25.E+06 |
| P26413 | Heat shock 70 kDa protein | GKELCKSINPDEAVAYGAAVQAAILSGQGD | P26413 [361-390] | 3032.4993 | 1.25.E+06 |
| I1JLC8 | Protein SLE2 | ASRQNNKQELDERARQGETVVPGG | I1JLC8 [2-25] | 2681.3350 | 1.24.E+06 |
| P09186 | Seed linoleate 9S-lipoxygenase-3 | DLNFTPREFDSFDEVHGLYSGGIKLPTDIISKI | P09186 [293-325] | 3723.8905 | 1.23.E+06 |
| P04405 | Glycinin G2 | LVPPQESQR | P04405 [473-481] | 1053.5687 | 1.21.E+06 |
| P09439 | Seed linoleate 9S-lipoxygenase-2 | MFSVPGVSGILNRG | P09439 [1-14] | 1475.7675 | 1.21.E+06 |
| P04405 | Glycinin G2 | PFSFLVPPQESQRRA | P04405 [469-483] | 1800.9391 | 1.20.E+06 |
| P09186; P08170 | Seed linoleate 9S-lipoxygenase-3; Seed linoleate 13S-lipoxygenase-1 | LTFRGIPNSISI | P09186 [846-857]; P08170 [828-839]; P38417 [842-853] | 1317.7525 | 1.19.E+06 |
| P19594 | 2S seed storage albumin protein | TMCRFGPMIQCDLSSDD | P19594 [142-158] | 2032.8231 | 1.19.E+06 |
| P01055 | Bowman-Birk type proteinase inhibitor | SYPAQCFCVDITDFCYEPCKPS | P01055 [83-104] | 2744.1136 | 1.19.E+06 |
| P11828; P04405 | Glycinin G3; Glycinin G2 | RPSIGNLAGAN | P11828 [425-435]; P04405 [429-439] | 1069.5749 | 1.19.E+06 |
| P11828 | Glycinin G3 | YLAGNQEQEFLQYQPQK | P11828 [180-196] | 2084.0083 | 1.18.E+06 |
| P04405; P04776 | Glycinin G2; Glycinin G1 | LNALPEEVIQH | P04405 [442-452]; P04776 [452-462] | 1262.6739 | 1.17.E+06 |
| Q01417 | 18 kDa seed maturation protein | MGHGHHTTGTGTGTATY | Q01417 [86-102] | 1686.7289 | 1.17.E+06 |
| Q8RVH5 | Basic 7S globulin 2 | VPIPQHHTNPTKPINLL | Q8RVH5 [25-41] | 1919.0861 | 1.15.E+06 |
| Q8RVH5 | Basic 7S globulin 2 | PQHHTNPTKPINLLVLP | Q8RVH5 [28-44] | 1919.0861 | 1.15.E+06 |
| P02858 | Glycinin G4 | GVEENICTLKL | P02858 [379-389] | 1275.6613 | 1.15.E+06 |
| P04405; P04776 | Glycinin G2; Glycinin G1 | LLNALPEEVIQHTFNLKS | P04405 [441-458]; P04776 [451-468] | 2066.1281 | 1.14.E+06 |
| P46519 | Desiccation protectant protein Lea14 homolog | SQLLDKAKNYVAEKV | P46519 [2-16] | 1747.9589 | 1.14.E+06 |
| P05046 | Lectin | SNLPHASSNIDPLDLTS | P05046 [262-278] | 1780.8712 | 1.14.E+06 |
| P02858 | Glycinin G4 | EPVVAISLLDTSNFNNQLDQTPRVF | P02858 [163-187] | 2817.4418 | 1.14.E+06 |
| P04776 | Glycinin G1 | DTPMIGTLAGANSLLNALPEEVIQHTFNLKSQQARQIKN | P04776 [438-476] | 4261.2398 | 1.13.E+06 |
| P02858 | Glycinin G4 | NQLDQTPRVF | P02858 [178-187] | 1217.6273 | 1.13.E+06 |
| P11828 | Glycinin G3 | NPFSFLVPPKESQR | P11828 [464-477] | 1645.8697 | 1.13.E+06 |
| F7J077; P0DO16 | Beta-conglycinin beta subunit 2; Beta-conglycinin alpha subunit 1 | EGALLLPHFN | F7J077 [281-290]; P25974 [281-290]; P0DO16 [447-456] | 1110.5942 | 1.13.E+06 |
| P11828 | Glycinin G3 | LNALPEEVIQQTFNLRR | P11828 [438-454] | 2041.1189 | 1.12.E+06 |
| P11828; P04405 | Glycinin G3; Glycinin G2 | DRPSIGNLAGANS | P11828 [424-436]; P04405 [428-440] | 1271.6339 | 1.12.E+06 |
| P0DO16 | Beta-conglycinin alpha subunit 1 | FLAGSQDNVISQIPSQVQELAFPGSAQAVEKL | P0DO16 [538-569] | 3371.7482 | 1.11.E+06 |
| P04405; P04776 | Glycinin G2; Glycinin G1 | SLLNALPEEVIQHTFNLKSQ | P04405 [440-459]; P04776 [450-469] | 2281.2187 | 1.10.E+06 |
| P04776 | Glycinin G1 | DTPMIGTLAGANSLLNALPEEVIQH | P04776 [438-462] | 2604.3338 | 1.10.E+06 |
| P13917 | Basic 7S globulin | VTPTKPIN | P13917 [25-32] | 869.5091 | 1.10.E+06 |
| P13917 | Basic 7S globulin | SIRINQHSVFPLN | P13917 [260-272] | 1524.8281 | 1.10.E+06 |
| P04405 | Glycinin G2 | DDEEEQPQCVETDKGCQRQSK | P04405 [276-296] | 2566.0780 | 1.09.E+06 |
| P09439 | Seed linoleate 9S-lipoxygenase-2 | MRKNVLDFN | P09439 [26-34] | 1136.5881 | 1.09.E+06 |
| P04776 | Glycinin G1 | TPMIGTLAGANSLLNALPEEVIQHTFNLKSQQARQ | P04776 [439-473] | 3806.9858 | 1.08.E+06 |
| I1JLC8 | Protein SLE2 | ASRQNNKQELDERARQGETVVPGGTG | I1JLC8 [2-27] | 2839.4041 | 1.08.E+06 |
| P62163 | Calmodulin-2 | YEEFVKVMMAK | P62163 [139-149] | 1374.6796 | 1.07.E+06 |
| Q39837 | Albumin-1 | GACSPFEVPPCRSRDCRCVP | Q39837 [24-43] | 2407.0522 | 1.04.E+06 |
| P04405; P04776 | Glycinin G2; Glycinin G1 | LAGANSLLNALPEEVIQHTFNLKSQQAR | P04405 [435-462]; P04776 [445-472] | 3062.6382 | 1.04.E+06 |
| P05046 | Lectin | SWDPPNPHIG | P05046 [163-172] | 1119.5218 | 1.04.E+06 |
| P04405 | Glycinin G2 | PPQESQRR | P04405 [475-482] | 997.5174 | 1.03.E+06 |
| P04405; P04776 | Glycinin G2; Glycinin G1 | ANSLLNALPEEVIQHTFNLK | P04405 [438-457]; P04776 [448-467] | 2251.2081 | 1.03.E+06 |
| P04776 | Glycinin G1 | LEFLEHAFSVDKQI | P04776 [226-239] | 1675.8690 | 1.03.E+06 |
| P04405; P04776 | Glycinin G2; Glycinin G1 | LAGNQEQEFLKY | P04405 [181-192]; P04776 [184-195] | 1439.7165 | 1.02.E+06 |
| Q8RVH5 | Basic 7S globulin 2 | VPIPQHHTNPTKPINL | Q8RVH5 [25-40] | 1806.0021 | 1.01.E+06 |
| P0DO16 | Beta-conglycinin alpha subunit 1 | ETLFKNQYGRIR | P0DO16 [204-215] | 1524.8281 | 1.00.E+06 |
| P02858 | Glycinin G4 | VFRAIPSE | P02858 [516-523] | 918.5043 | 1.00.E+06 |
| P04776 | Glycinin G1 | IKNNNPFKF | P04776 [474-482] | 1121.6102 | 9.92.E+05 |
| P02858 | Glycinin G4 | VLAHSYNL | P02858 [524-531] | 916.4887 | 9.70.E+05 |
| P04405; P04776 | Glycinin G2; Glycinin G1 | NALPEEVIQHTFNLK | P04405 [443-457]; P04776 [453-467] | 1752.9279 | 9.70.E+05 |
| P04776 | Glycinin G1 | NNPFKFLVPPQESQKRAV | P04776 [477-494] | 2099.1396 | 9.68.E+05 |
| P08170 | Seed linoleate 13S-lipoxygenase-1 | MFSAGHKIKGTVVLMPKNELEVNPDGSAVD | P08170 [1-30] | 3241.6232 | 9.67.E+05 |
| P13917 | Basic 7S globulin | VTPTKPINLVVLPVQNDGS | P13917 [25-43] | 1991.1172 | 9.64.E+05 |
| P29531 | P24 oleosin isoform B | TTVPPHSVQVHTTTHRYEAGVVPPARFE | P29531 [2-29] | 3155.6021 | 9.58.E+05 |
| P11827; P0DO16 | Beta-conglycinin alpha' subunit; Beta-conglycinin alpha subunit 1 | YRILEFN | P11827 [249-255]; P0DO16 [233-239] | 954.5043 | 9.51.E+05 |
| P04776 | Glycinin G1 | VKGGLSVIKPPTD | P04776 [258-270] | 1310.7678 | 9.51.E+05 |
| P04776 | Glycinin G1 | LEFLEH | P04776 [226-231] | 787.3985 | 9.38.E+05 |
| Q8RVH5 | Basic 7S globulin 2 | HSVFPPN | Q8RVH5 [273-279] | 797.3941 | 9.30.E+05 |
| P02858 | Glycinin G4 | ISSSKLN | P02858 [24-30] | 748.4199 | 9.26.E+05 |
| P04405 | Glycinin G2 | LREQAQQNECQIQKLNALKPDNRIE | P04405 [19-43] | 3036.5643 | 9.25.E+05 |
| P09439 | Seed linoleate 9S-lipoxygenase-2 | MFSVPGVSGILNRGG | P09439 [1-15] | 1532.7890 | 9.21.E+05 |
| P0DO16 | Beta-conglycinin alpha subunit 1 | VQELAFPGSAQAVEK | P0DO16 [554-568] | 1573.8221 | 9.18.E+05 |
| P11828; P04776 | Glycinin G3; Glycinin G1 | AMFVPHYNLNAN | P11828 [354-365]; P04776 [368-379] | 1390.6572 | 9.18.E+05 |
| P11828 | Glycinin G3 | NPFSFLVPPKESQRRV | P11828 [464-479] | 1901.0392 | 9.16.E+05 |
| Q8RVH5 | Basic 7S globulin 2 | TNPTKPINLLVLPVQN | Q8RVH5 [32-47] | 1761.0269 | 9.12.E+05 |
| F7J077 | Beta-conglycinin beta subunit 2 | LKVREDENNPFYL | F7J077 [24-36]; P25974 [24-36] | 1636.8330 | 9.11.E+05 |
| P04776 | Glycinin G1 | TLAGANSLLNALPEEVIQHTFNLKSQQARQ | P04776 [444-473] | 3291.7444 | 9.10.E+05 |
| P13917 | Basic 7S globulin | VTPTKPINLVVLPVQNDGSTGLHWANLQ | P13917 [25-52] | 3011.6313 | 9.06.E+05 |
| P08170 | Seed linoleate 13S-lipoxygenase-1 | ELEVNPDGSAVD | P08170 [19-30] | 1244.5641 | 9.03.E+05 |
| P09186 | Seed linoleate 9S-lipoxygenase-3 | IDLSVIEILSRHA | P09186 [765-777]; P24095 [772-784] | 1465.8373 | 9.01.E+05 |
| P04405 | Glycinin G2 | YLAGNQEQEFLKYQQQ | P04405 [180-195] | 1986.9556 | 9.00.E+05 |
| P05046 | Lectin | FASNLPHASSNID | P05046 [260-272] | 1372.6492 | 8.90.E+05 |
| P09186 | Seed linoleate 9S-lipoxygenase-3 | YTLLLPSSKEGLTFRGIPNSISI | P09186 [835-857] | 2506.3916 | 8.86.E+05 |
| P0DO16 | Beta-conglycinin alpha subunit 1 | EIPRPRPRPQHPEREPQQPGE | P0DO16 [72-92] | 2530.3022 | 8.85.E+05 |
| Q04672 | Sucrose-binding protein | FFPFELPREERGR | Q04672 [508-520] | 1679.8653 | 8.83.E+05 |
| P05046 | Lectin | LPHASSNIDPLDLT | P05046 [264-277] | 1492.7642 | 8.80.E+05 |
| P11828; P04776 | Glycinin G3; Glycinin G1 | GIDETICTMRLRHNIGQTSSPDI | P11828 [297-319]; P04776 [311-333] | 2614.2712 | 8.72.E+05 |
| P11827 | Beta-conglycinin alpha' subunit | VQELAFPGSAKDIENLIKSQSESYFVDAQPQQKEEGN | P11827 [570-606] | 4124.0095 | 8.64.E+05 |
| P04405 | Glycinin G2 | EEQPQCVETDKGCQRQS | P04405 [279-295] | 2078.8866 | 8.60.E+05 |
| P04405; P04776 | Glycinin G2; Glycinin G1 | AGANSLLNALPEEVIQHTFNLKSQQA | P04405 [436-461]; P04776 [446-471] | 2793.4530 | 8.55.E+05 |
| P01071; P01070 | Trypsin inhibitor B; Trypsin inhibitor A | IGISIDHDDGTRRL | P01071 [148-161]; P01070 [172-185] | 1567.8187 | 8.55.E+05 |
| P02858 | Glycinin G4 | SQVSELKYEGNWGPLVNPESQQGSPR | P02858 [534-559] | 2886.4017 | 8.45.E+05 |
| P04405 | Glycinin G2 | DDDEEEQPQCVETDKGCQRQ | P04405 [275-294] | 2465.9780 | 8.43.E+05 |
| P09186 | Seed linoleate 9S-lipoxygenase-3 | GPVQMPYTLLLPSSKEGLTFRGIPNSISI | P09186 [829-857] | 3115.6860 | 8.40.E+05 |
| P04776 | Glycinin G1 | NNPFKFLVPPQESQ | P04776 [477-490] | 1644.8380 | 8.39.E+05 |
| Q8RVH5 | Basic 7S globulin 2 | LLVLPVQNDASTGLHWANLQK | Q8RVH5 [40-60] | 2317.2663 | 8.39.E+05 |
| P04347 | Glycinin G5 | PRVFYLAGNPDIEHPETMQQ | P04347 [184-203] | 2342.1234 | 8.33.E+05 |
| Q8RVH5 | Basic 7S globulin 2 | VPIPQHHTNPTKPINLLVLPVQNDA | Q8RVH5 [25-49] | 2755.5254 | 8.31.E+05 |
| P11828; P04405 | Glycinin G3; Glycinin G2 | FEYVSFKTNDRPSIGNLAGANSL | P11828 [415-437]; P04405 [419-441] | 2500.2467 | 8.25.E+05 |
| Q8RVH5 | Basic 7S globulin 2 | VPIPQHHTNPTKPINLLVLPVQNDASTGLHWANLQK | Q8RVH5 [25-60] | 3991.1665 | 8.25.E+05 |
| P11828 | Glycinin G3 | LAGANSLLNALPEEVIQQTFNLRRQQA | P11828 [431-457] | 2994.6119 | 8.22.E+05 |
| P02858 | Glycinin G4 | GNWGPLVNPESQQGSPR | P02858 [543-559] | 1822.8831 | 8.18.E+05 |
| P05046 | Lectin | LPHASSNIDPLDLTSFVL | P05046 [264-281] | 1939.0171 | 8.18.E+05 |
| P05046 | Lectin | ILQGDAIVTSSGKLQL | P05046 [51-66] | 1642.9374 | 8.15.E+05 |
| P01063 | Bowman-Birk type proteinase inhibitor C-II | DESSKPCCDLCMCTA | P01063 [15-29] | 1833.6580 | 8.09.E+05 |
| P05046 | Lectin | AETVSFSWNKFVPKQPN | P05046 [33-49] | 1979.0021 | 8.07.E+05 |
| P04405; P04776 | Glycinin G2; Glycinin G1 | AGANSLLNALPEEVIQHTFN | P04405 [436-455]; P04776 [446-465] | 2138.0877 | 8.04.E+05 |
| P01064 | Bowman-Birk type proteinase inhibitor D-II | EYSKPCCDL | P01064 [19-27] | 1171.4758 | 8.01.E+05 |
| P11827 | Beta-conglycinin alpha' subunit | VQELAFPGSAKDIENLIKSQ | P11827 [570-589] | 2187.1656 | 7.91.E+05 |
| P09186 | Seed linoleate 9S-lipoxygenase-3 | MLGGLLHRGH | P09186 [1-10] | 1132.6044 | 7.91.E+05 |
| P04405; P04776 | Glycinin G2; Glycinin G1 | LAGNQEQEFLK | P04405 [181-191]; P04776 [184-194] | 1276.6532 | 7.83.E+05 |
| P11828; P04405 | Glycinin G3; Glycinin G2 | FKTNDRPSIGNLAGANSL | P11828 [420-437]; P04405 [424-441] | 1874.9719 | 7.83.E+05 |
| P04405 | Glycinin G2 | LKSQQARQVKNNNPFSFLVPPQESQRR | P04405 [456-482] | 3196.7086 | 7.80.E+05 |
| P02858 | Glycinin G4 | VSELKYEGNWGPLVNPESQQGSP | P02858 [536-558] | 2515.2100 | 7.72.E+05 |
| P04347 | Glycinin G5 | GVEENICTMK | P04347 [345-354] | 1180.5337 | 7.71.E+05 |
| P11828 | Glycinin G3 | SLLNALPEEVIQQTFN | P11828 [436-451] | 1815.9487 | 7.69.E+05 |
| P04776 | Glycinin G1 | FSSREQPQQNECQIQKL | P04776 [20-36] | 2120.0189 | 7.66.E+05 |
| P49045 | Vacuolar-processing enzyme | TYCPGMDPSPPPE | P49045 [251-263] | 1447.5868 | 7.56.E+05 |
| P05046 | Lectin | DLTSFVLHEAI | P05046 [275-285] | 1244.6521 | 7.53.E+05 |
| P04405; P04776 | Glycinin G2; Glycinin G1 | ANSLLNALPEEVIQHTFNLKSQQA | P04405 [438-461]; P04776 [448-471] | 2665.3944 | 7.49.E+05 |
| P05046 | Lectin | IPGESHDVLSWSFASNLPHASSNIDPLDLT | P05046 [248-277] | 3219.5593 | 7.43.E+05 |
| P04405; P04776 | Glycinin G2; Glycinin G1 | ANSLLNALPEEVIQHTFNLKSQQARQ | P04405 [438-463]; P04776 [448-473] | 2949.5541 | 7.41.E+05 |
| Q04672 | Sucrose-binding protein | SFFFPFELPREERGR | Q04672 [506-520] | 1913.9657 | 7.27.E+05 |
| P04776 | Glycinin G1 | LAGNQEQEFLKYQQEQGGHQS | P04776 [184-204] | 2419.1273 | 7.13.E+05 |
| Q04672 | Sucrose-binding protein | FFPFELPREERG | Q04672 [508-519] | 1523.7641 | 7.12.E+05 |
| P02858 | Glycinin G4 | EPVVAISLLDTSNFNNQLDQTPRVFYLAGN | P02858 [163-192] | 3335.6907 | 7.11.E+05 |
| P04405; P04776 | Glycinin G2; Glycinin G1 | TPVVAVSIIDTNSLENQLDQMPRRFY | P04405 [155-180]; P04776 [158-183] | 3006.5353 | 7.06.E+05 |
| P01070 | Trypsin inhibitor A | KPLVVQFQKLDKESL | P01070 [191-205] | 1772.0317 | 7.06.E+05 |
| P11828 | Glycinin G3 | LAGANSLLNALPEEVIQQTFNLRR | P11828 [431-454] | 2667.4577 | 7.05.E+05 |
| Q04672 | Sucrose-binding protein | EEDPELVTCKHQCQQ | Q04672 [35-49] | 1900.8164 | 7.05.E+05 |
| P28759 | Superoxide dismutase [Fe], chloroplastic | ASLGGLQNVSGINFLIKEGPKVN | P28759 [2-24] | 2397.3136 | 6.96.E+05 |
| P04405; P04776 | Glycinin G2; Glycinin G1 | AGANSLLNALPEEVIQHTFNLKSQQARQ | P04405 [436-463]; P04776 [446-473] | 3077.6127 | 6.83.E+05 |
| P01064 | Bowman-Birk type proteinase inhibitor D-II | TRSMPPQCSCEDIR | P01064 [31-44] | 1736.7513 | 6.81.E+05 |
| P04776 | Glycinin G1 | FSSREQPQ | P04776 [20-27] | 978.4639 | 6.81.E+05 |
| P0DO16 | Beta-conglycinin alpha subunit 1 | PFLFGSNRFETL | P0DO16 [195-206] | 1427.7318 | 6.80.E+05 |
| P04776 | Glycinin G1 | SLLNALPEEVIQHTFNLKSQQARQIK | P04776 [450-475] | 3005.6531 | 6.78.E+05 |
| P04405; P04776 | Glycinin G2; Glycinin G1 | PEEVIQHTFNLKS | P04405 [446-458]; P04776 [456-468] | 1541.7958 | 6.68.E+05 |
| P08170 | Seed linoleate 13S-lipoxygenase-1 | YTLLYPSSEEGLTFRGIPNSISI | P08170 [817-839]; P38417 [831-853] | 2557.3185 | 6.65.E+05 |
| P05046 | Lectin | ILQGDAIVTSSGKL | P05046 [51-64] | 1401.7948 | 6.61.E+05 |
| P04405; P04776 | Glycinin G2; Glycinin G1 | LNALPEEVIQHTFNLKSQQAR | P04405 [442-462]; P04776 [452-472] | 2436.2994 | 6.60.E+05 |
| P11827 | Beta-conglycinin alpha' subunit | SAKDIENLIKSQSESYFVDAQPQQKEEGN | P11827 [578-606] | 3282.5761 | 6.49.E+05 |
| P08170 | Seed linoleate 13S-lipoxygenase-1 | SAGHKIKGTVVLMPKNELEVN | P08170 [3-23] | 2264.2431 | 6.45.E+05 |
| P04347 | Glycinin G5 | LAGNPDIEHPETMQQQQQ | P04347 [189-206] | 2063.9451 | 6.40.E+05 |
| P04405 | Glycinin G2 | FKTNDRPSIGNLAGANSLLNALPEEVIQHTFN | P04405 [424-455] | 3480.7870 | 6.39.E+05 |
| P0DO16 | Beta-conglycinin alpha subunit 1 | AIGINAENNQRNF | P0DO16 [526-538] | 1460.7241 | 6.36.E+05 |
| P05046 | Lectin | SFASNLPHASSNID | P05046 [259-272] | 1459.6812 | 6.33.E+05 |
| P04405; P04776 | Glycinin G2; Glycinin G1 | NALPEEVIQHTFN | P04405 [443-455]; P04776 [453-465] | 1511.7489 | 6.32.E+05 |
| P22895 | P34 probable thiol protease | YFASYPTKEESETLVSAR | P22895 [350-367] | 2078.0077 | 6.31.E+05 |
| P11827 | Beta-conglycinin alpha' subunit | KNPFHFN | P11827 [209-215] | 903.4472 | 6.31.E+05 |
| P04405 | Glycinin G2 | GIDETICTMRLRQNIGQNSSPDIYN | P04405 [301-325] | 2911.3673 | 6.29.E+05 |
| P26413 | Heat shock 70 kDa protein | KSINPDEAVAYGAAVQAAILSGQGD | P26413 [366-390] | 2445.2256 | 6.28.E+05 |
| P08170 | Seed linoleate 13S-lipoxygenase-1 | GTVVLMPKNELEVN | P08170 [10-23] | 1542.8196 | 6.21.E+05 |
| P05046 | Lectin | IPGESHDVLSWSFASNLPHA | P05046 [248-267] | 2164.0458 | 6.20.E+05 |
| P05046 | Lectin | IDPLDLTSFVLHE | P05046 [271-283] | 1498.7788 | 6.18.E+05 |
| Q01417 | 18 kDa seed maturation protein | AMPGHGTGQPTGHVTE | Q01417 [119-134] | 1576.7173 | 6.18.E+05 |
| P04405 | Glycinin G2 | DDDEEEQPQCVETDKGCQRQSK | P04405 [275-296] | 2681.1050 | 6.17.E+05 |
| P04405; P04776 | Glycinin G2; Glycinin G1 | ANSLLNALPEEVIQHTFNLKSQQ | P04405 [438-460]; P04776 [448-470] | 2594.3573 | 6.13.E+05 |
| P09186 | Seed linoleate 9S-lipoxygenase-3 | PYTLLLPSSKEGLTFRGIPNSISI | P09186 [834-857] | 2603.4443 | 6.11.E+05 |
| P04405 | Glycinin G2 | GIDETICTMRLRQNIGQNSSPDIYNPQA | P04405 [301-328] | 3191.5208 | 6.08.E+05 |
| P08170 | Seed linoleate 13S-lipoxygenase-1 | GNRLGPVQLPYTLLYPSSEEGLTFRGIPNSISI | P08170 [807-839] | 3588.9061 | 6.06.E+05 |
| O04132 | Protein SRC1 | SGIIHKIEETLHVGG | O04132 [2-16] | 1631.8752 | 6.06.E+05 |
| P04405 | Glycinin G2 | GELQEGGVLIVPQNFAVAAK | P04405 [394-413] | 2040.1124 | 5.95.E+05 |
| P11828 | Glycinin G3 | SFQNQLDQMPRR | P11828 [167-178] | 1519.7434 | 5.94.E+05 |
| P0DO16 | Beta-conglycinin alpha subunit 1 | PQHPEREPQQPGE | P0DO16 [80-92] | 1528.7139 | 5.93.E+05 |
| Q01417 | 18 kDa seed maturation protein | MQGGKKAGESIKETATNIGASAK | Q01417 [1-23] | 2319.1973 | 5.90.E+05 |
| P04405 | Glycinin G2 | IVRNLQGENEEEDSGAIVTVKGGLR | P04405 [236-260] | 2683.4010 | 5.85.E+05 |
| P04405; P04776 | Glycinin G2; Glycinin G1 | YLAGNQEQEFLKYQQ | P04405 [180-194]; P04776 [183-197] | 1858.8970 | 5.85.E+05 |
| P04405 | Glycinin G2 | SLLNALPEEVIQHTFNLKSQQARQVKN | P04405 [440-466] | 3105.6804 | 5.83.E+05 |
| Q01417 | 18 kDa seed maturation protein | GEYGQPMGAHQT | Q01417 [106-117] | 1275.5423 | 5.74.E+05 |
| P02858 | Glycinin G4 | EPVVAISLLDTSNFNNQLDQTPRVFY | P02858 [163-188] | 2980.5051 | 5.69.E+05 |
| P04405 | Glycinin G2 | GFAPEFLKEAFGVN | P04405 [220-233] | 1525.7686 | 5.68.E+05 |
| P05046 | Lectin | SNLPHASSNIDPLD | P05046 [262-275] | 1479.7074 | 5.63.E+05 |
| P05046 | Lectin | PLDLTSFV | P05046 [273-280] | 891.4822 | 5.57.E+05 |
| P05046 | Lectin | FASNLPHASSNIDPLD | P05046 [260-275] | 1697.8129 | 5.55.E+05 |
| P05046 | Lectin | SNLPHASSNIDPLDLTSFVLHEA | P05046 [262-284] | 2477.2307 | 5.54.E+05 |
| P02858 | Glycinin G4 | TGDEPVVAISLLDTSNFNNQLDQTPRVFY | P02858 [160-188] | 3253.6012 | 5.51.E+05 |
| P04347 | Glycinin G5 | DHPPQRPSRPEQQEP | P04347 [321-335] | 1797.8627 | 5.48.E+05 |
| P04776 | Glycinin G1 | FSSREQPQQNECQIQKLNALKPD | P04776 [20-42] | 2758.3577 | 5.48.E+05 |
| P04347 | Glycinin G5 | LAGNPDIEHPETMQQ | P04347 [189-203] | 1679.7694 | 5.47.E+05 |
| P09439 | Seed linoleate 9S-lipoxygenase-2 | RKNVLDFN | P09439 [27-34] | 1005.5476 | 5.45.E+05 |
| P0DO16 | Beta-conglycinin alpha subunit 1 | GEIPRPRPRPQHPE | P0DO16 [71-84] | 1665.8932 | 5.44.E+05 |
| P02858 | Glycinin G4 | GVEENICTLKLHENIARPSR | P02858 [379-398] | 2336.2139 | 5.43.E+05 |
| P02858; P04347 | Glycinin G4; Glycinin G5 | FYNPKAGRISTLN | P02858 [401-413]; P04347 [367-379] | 1480.7907 | 5.39.E+05 |
| P01071; P01070 | Trypsin inhibitor B; Trypsin inhibitor A | SDITAFGGIRA | P01071 [21-31]; P01070 [45-55] | 1107.5793 | 5.34.E+05 |
| Q01417 | 18 kDa seed maturation protein | TSAMPGHGTGQPTGHVTEGVVGSHPIGTNRGPGGTATAH | Q01417 [117-155] | 3688.7633 | 5.31.E+05 |
| P02858 | Glycinin G4 | IIIAQGKGALGVAIPGCPETFEEPQEQSN | P02858 [92-120] | 3053.5248 | 5.28.E+05 |
| P04405 | Glycinin G2 | DEEEQPQCVETDKGCQRQSKR | P04405 [277-297] | 2607.1522 | 5.26.E+05 |
| P19594 | 2S seed storage albumin protein | TMCRFGPMIQCD | P19594 [142-153] | 1515.6211 | 5.23.E+05 |
| P01064 | Bowman-Birk type proteinase inhibitor D-II | YSKPCCDLCMC | P01064 [20-30] | 1493.5350 | 5.22.E+05 |
| P04347 | Glycinin G5 | YLAGNPDIEHPETMQQQQQ | P04347 [188-206] | 2227.0084 | 5.17.E+05 |
| P02858 | Glycinin G4 | AVTSYLKDVFRAIPSEVLAHSYNL | P02858 [508-531] | 2693.4297 | 5.13.E+05 |
| P04405 | Glycinin G2 | LREQAQQN | P04405 [19-26] | 986.5014 | 5.12.E+05 |
| P05046 | Lectin | SFASNLPHASSNIDPLDLTSFVLHEA | P05046 [259-284] | 2782.3683 | 5.10.E+05 |
| P04347 | Glycinin G5 | PDIEHPETMQQQQ | P04347 [193-205] | 1580.7010 | 5.09.E+05 |
| P09186 | Seed linoleate 9S-lipoxygenase-3 | PVQMPYTLLLPSSKEGLTFRGIPNSISI | P09186 [830-857] | 3058.6646 | 5.08.E+05 |
| P08170 | Seed linoleate 13S-lipoxygenase-1 | PYTLLYPSSEEGLTFRGIPNSISI | P08170 [816-839]; P38417 [830-853] | 2654.3712 | 5.06.E+05 |
| P04347 | Glycinin G5 | LAGNPDIEHPETMQQQ | P04347 [189-204] | 1807.8279 | 5.05.E+05 |
| P04776 | Glycinin G1 | SLLNALPEEVIQHTFNLKSQQARQIKNNNPFKF | P04776 [450-482] | 3867.0664 | 5.04.E+05 |
| P04405 | Glycinin G2 | NPFSFLVPPQESQRRAV | P04405 [468-484] | 1972.0399 | 5.02.E+05 |
| P04405; P04776 | Glycinin G2; Glycinin G1 | GANSLLNALPEEVIQHTFNLKS | P04405 [437-458]; P04776 [447-468] | 2395.2616 | 4.97.E+05 |
| P04405; P04776 | Glycinin G2; Glycinin G1 | LLNALPEEVIQHTFNLKSQQ | P04405 [441-460]; P04776 [451-470] | 2322.2452 | 4.92.E+05 |
| C6T0L2 | Protein SLE3 | ASHQQNKQELDERARQGETVVPGGTGGKSLEAQQHLAE | C6T0L2 [2-39] | 4126.0297 | 4.89.E+05 |
| P04405 | Glycinin G2 | SLLNALPEEVIQHTFNLKSQQARQVKNNNPFSF | P04405 [440-472] | 3811.9878 | 4.88.E+05 |
| P04405 | Glycinin G2 | GELQEGGVLIVPQNFAVA | P04405 [394-411] | 1840.9803 | 4.87.E+05 |
| P11827 | Beta-conglycinin alpha' subunit | SQSESYFVDAQPQQKEEGN | P11827 [588-606] | 2170.9524 | 4.78.E+05 |
| P02858 | Glycinin G4 | ISSSKLNECQLNNLNALEPDHRVE | P02858 [24-47] | 2780.3632 | 4.74.E+05 |
| Q8RVH5; P13917 | Basic 7S globulin 2; Basic 7S globulin | THQCLSCPA | Q8RVH5 [106-114]; P13917 [99-107] | 1073.4503 | 4.72.E+05 |
| P62163 | Calmodulin-2 | GQINYEEFVKVMMAK | P62163 [135-149] | 1786.8866 | 4.72.E+05 |
| P02858 | Glycinin G4 | TYNTGDEPVVAISLLDTSNFNNQLDQTPRVF | P02858 [157-187] | 3468.6918 | 4.70.E+05 |
| P19594 | 2S seed storage albumin protein | SKWQHQQDSCRKQLQGVNLTPCEKH | P19594 [22-46] | 3092.4901 | 4.59.E+05 |
| P11828 | Glycinin G3 | FSFREQPQQNE | P11828 [20-30] | 1409.6444 | 4.56.E+05 |
| P01064 | Bowman-Birk type proteinase inhibitor D-II | DEYSKPCCDLCMC | P01064 [18-30] | 1737.6046 | 4.55.E+05 |
| P0DO16 | Beta-conglycinin alpha subunit 1 | NVISQIPSQVQELAFPGSAQAVEKL | P0DO16 [545-569] | 2653.4196 | 4.53.E+05 |
| P26987 | Stress-induced protein SAM22 | YLLAHPDYN | P26987 [150-158] | 1105.5313 | 4.47.E+05 |
| P04347 | Glycinin G5 | GVEENICTMKLHENIAR | P04347 [345-361] | 2013.9845 | 4.44.E+05 |
| P02858 | Glycinin G4 | VFRAIPSEVLAHSYN | P02858 [516-530] | 1702.8911 | 4.41.E+05 |
| Q01417 | 18 kDa seed maturation protein | NTAAKQSATTAGHMGHGHHTTGTGTGTATY | Q01417 [73-102] | 2925.3293 | 4.37.E+05 |
| P04405 | Glycinin G2 | GIDETICTMRLRQ | P04405 [301-313] | 1592.7883 | 4.29.E+05 |
| P04405; P04776 | Glycinin G2; Glycinin G1 | LAGANSLLNALPEEVIQHTFNLKSQ | P04405 [435-459]; P04776 [445-469] | 2707.4414 | 4.25.E+05 |
| P01070 | Trypsin inhibitor A | AMDGWFRL | P01070 [137-144] | 995.4768 | 4.23.E+05 |
| P04405 | Glycinin G2 | NNPFSFLVPPQESQRRAVA | P04405 [467-485] | 2157.1200 | 4.21.E+05 |
| P08170 | Seed linoleate 13S-lipoxygenase-1 | SLQGNRLGPVQLPYTLLYPSSEEGLTFRGIPNSISI | P08170 [804-839] | 3917.0807 | 4.21.E+05 |
| P04405 | Glycinin G2 | VRNLQGENEEEDSGAIVTVKGGLRV | P04405 [237-261] | 2669.3853 | 4.19.E+05 |
| P04405 | Glycinin G2 | RPSIGNLAGANSLLNALPEEVIQHT | P04405 [429-453] | 2614.3947 | 4.18.E+05 |
| Q01417 | 18 kDa seed maturation protein | MQGGKKAGESIKETATNI | Q01417 [1-18] | 1904.9746 | 4.10.E+05 |
| P04776 | Glycinin G1 | FLVPPQESQKRAV | P04776 [482-494] | 1498.8376 | 4.04.E+05 |
| P04405 | Glycinin G2 | DDEEEQPQCVETDKGCQRQSKR | P04405 [276-297] | 2722.1791 | 4.02.E+05 |
| P0DO16 | Beta-conglycinin alpha subunit 1 | VISQIPSQVQELAFPGSAQAVEKLLKN | P0DO16 [546-572] | 2894.5986 | 4.02.E+05 |
| P0DO16 | Beta-conglycinin alpha subunit 1 | NVISQIPSQVQELAFPGSAQAVEKLLKN | P0DO16 [545-572] | 3008.6415 | 4.01.E+05 |
| P05046 | Lectin | AETVSFSWNKFVPKQPNM | P05046 [33-50] | 2110.0426 | 4.00.E+05 |
| P25272 | Kunitz-type trypsin inhibitor KTI1 | VVQFQKFRSST | P25272 [192-202] | 1326.7165 | 3.89.E+05 |
| P01064 | Bowman-Birk type proteinase inhibitor D-II | MPPQCSCEDIR | P01064 [34-44] | 1392.5705 | 3.89.E+05 |
| P0DO16 | Beta-conglycinin alpha subunit 1 | LAGSQDNVISQIPSQVQELAFPGSAQAVEKLLKNQRESY | P0DO16 [539-577] | 4243.1993 | 3.88.E+05 |
| O04132 | Protein SRC1 | SGIIHKIEETLHVG | O04132 [2-15] | 1574.8537 | 3.88.E+05 |
| P08170 | Seed linoleate 13S-lipoxygenase-1 | RLGPVQLPYTLLYPSSEEGLTFRGIPN | P08170 [809-835] | 3017.6095 | 3.87.E+05 |
| P02858 | Glycinin G4 | PESQQGSPR | P02858 [551-559] | 985.4698 | 3.86.E+05 |
| P04405 | Glycinin G2 | EGSNILSGFAPEFLKE | P04405 [213-228] | 1737.8694 | 3.85.E+05 |
| P04405 | Glycinin G2 | DDEEEQPQCVETDKGCQRQSKRS | P04405 [276-298] | 2809.2112 | 3.84.E+05 |
| Q8RVH5 | Basic 7S globulin 2 | PIPQHHTN | Q8RVH5 [26-33] | 943.4744 | 3.81.E+05 |
| P01070 | Trypsin inhibitor A | KPLVVQFQKLDKESLA | P01070 [191-206] | 1843.0688 | 3.80.E+05 |
| P04405 | Glycinin G2 | LREQAQQNECQIQKLNA | P04405 [19-35] | 2071.0349 | 3.79.E+05 |
| P01064 | Bowman-Birk type proteinase inhibitor D-II | EYSKPCCDLCMC | P01064 [19-30] | 1622.5776 | 3.79.E+05 |
| P01070 | Trypsin inhibitor A | KPLVVQFQKLDKES | P01070 [191-204] | 1658.9476 | 3.79.E+05 |
| Q04672 | Sucrose-binding protein | SFFFPFELPREERG | Q04672 [506-519] | 1757.8646 | 3.79.E+05 |
| P04405; P04776 | Glycinin G2; Glycinin G1 | EDTPVVAVSIIDTNSLENQLDQMPRRF | P04405 [153-179]; P04776 [156-182] | 3087.5415 | 3.73.E+05 |
| P04405 | Glycinin G2 | LREQAQQNECQIQKL | P04405 [19-33] | 1885.9549 | 3.73.E+05 |
| P0DO16 | Beta-conglycinin alpha subunit 1 | GEIPRPRPRPQHPEREP | P0DO16 [71-87] | 2048.0897 | 3.71.E+05 |
| P0DO16 | Beta-conglycinin alpha subunit 1 | IPRPRPRPQHPEREPQQPGE | P0DO16 [73-92] | 2401.2596 | 3.67.E+05 |
| P11827 | Beta-conglycinin alpha' subunit | VISQIPSQVQELAFPGSAKDIENLIKSQ | P11827 [562-589] | 3039.6361 | 3.65.E+05 |
| P01055 | Bowman-Birk type proteinase inhibitor | ITDFCYEPCKPSEDDKEN | P01055 [93-110] | 2246.9216 | 3.60.E+05 |
| P04405 | Glycinin G2 | LREQAQQNECQIQKLNALKPD | P04405 [19-39] | 2524.2936 | 3.59.E+05 |
| P01064 | Bowman-Birk type proteinase inhibitor D-II | RSMPPQCSCEDI | P01064 [32-43] | 1479.6025 | 3.53.E+05 |
| P04405 | Glycinin G2 | EEEQPQCVETDKGCQRQSK | P04405 [278-296] | 2336.0242 | 3.49.E+05 |
| P0DO16 | Beta-conglycinin alpha subunit 1 | EIPRPRPRPQHPEREPQQPGEKE | P0DO16 [72-94] | 2787.4397 | 3.47.E+05 |
| P05046 | Lectin | SHDVLSWSFASNLPHASSNID | P05046 [252-272] | 2284.0629 | 3.42.E+05 |
| Q04672 | Sucrose-binding protein | FFPFELPRE | Q04672 [508-516] | 1181.5990 | 3.37.E+05 |
| P04347 | Glycinin G5 | EDQPRPDHPPQRPSRP | P04347 [315-330] | 1908.9423 | 3.36.E+05 |
| P04405 | Glycinin G2 | DRPSIGNLAGANSLLNALPEEVIQHT | P04405 [428-453] | 2729.4217 | 3.36.E+05 |
| P04405 | Glycinin G2 | NLAGANSLLNALPEEVIQHTFN | P04405 [434-455] | 2365.2147 | 3.30.E+05 |
| P04776 | Glycinin G1 | FSSREQPQQ | P04776 [20-28] | 1106.5225 | 3.27.E+05 |
| P04405; P04776 | Glycinin G2; Glycinin G1 | GANSLLNALPEEVIQHTFNLKSQQARQ | P04405 [437-463]; P04776 [447-473] | 3006.5756 | 3.19.E+05 |
| P04405 | Glycinin G2 | EEQPQCVETDKGCQRQ | P04405 [279-294] | 1991.8546 | 3.14.E+05 |
| P04405 | Glycinin G2 | PQCVETDKGCQR | P04405 [282-293] | 1477.6522 | 3.05.E+05 |
| P04405 | Glycinin G2 | EQPQCVETDKGCQ | P04405 [280-292] | 1578.6523 | 3.04.E+05 |
| Q8RVH5 | Basic 7S globulin 2 | VPIPQHHTNPTKPINLLVLPVQNDASTGLH | Q8RVH5 [25-54] | 3250.7695 | 3.03.E+05 |
| P02858 | Glycinin G4 | AVTSYLKDVFRAIPSEVL | P02858 [508-525] | 2008.1114 | 3.00.E+05 |
| P04405 | Glycinin G2 | DRPSIGNLAGANSLLNALPEEVIQHTFNLKSQQAR | P04405 [428-462] | 3801.9995 | 2.98.E+05 |
| P04405 | Glycinin G2 | FKTNDRPSIGNLAGANSLLNALPEEVIQHTFNLKS | P04405 [424-458] | 3808.9981 | 2.97.E+05 |
| P04405 | Glycinin G2 | PQCVETDKGCQRQS | P04405 [282-295] | 1692.7428 | 2.86.E+05 |
| P04347 | Glycinin G5 | ALEPDHRVESEGGLIETWN | P04347 [40-58] | 2152.0305 | 2.84.E+05 |
| P02858 | Glycinin G4 | EPVVAISLLDTSNFN | P02858 [163-177] | 1618.8323 | 2.78.E+05 |
| P0DO16 | Beta-conglycinin alpha subunit 1 | PQHPEREPQQPGEKE | P0DO16 [80-94] | 1785.8515 | 2.61.E+05 |
| Q01417 | 18 kDa seed maturation protein | HMGHGHHTTGTGTGTATY | Q01417 [85-102] | 1823.7878 | 2.54.E+05 |
| P05046 | Lectin | LPHASSNIDPLDLTSFVLHE | P05046 [264-283] | 2205.1186 | 2.47.E+05 |
| Q9M4T8 | Proteasome subunit alpha type-5 | NVDIAKVAPTYHLYTPSEVEAVISRL | Q9M4T8 [212-237] | 2885.5407 | 2.46.E+05 |
| P04405; P04776 | Glycinin G2; Glycinin G1 | SIIDTNSLENQLDQMPRRF | P04405 [161-179]; P04776 [164-182] | 2277.1292 | 2.37.E+05 |
| P02858 | Glycinin G4 | AVTSYLKDVFRAIPSEVLAH | P02858 [508-527] | 2216.2074 | 2.37.E+05 |
| P04405 | Glycinin G2 | PFSFLVPPQESQRR | P04405 [469-482] | 1729.9020 | 2.17.E+05 |
| P04405 | Glycinin G2 | DDDEEEQPQCVETDKGCQRQSKR | P04405 [275-297] | 2837.2061 | 2.15.E+05 |
| P04347 | Glycinin G5 | EEEDQPRPDHPPQRPSRPEQQEP | P04347 [313-335] | 2778.2826 | 1.98.E+05 |
| P08170 | Seed linoleate 13S-lipoxygenase-1 | NRLGPVQLPYTLLYPSSEEGLTFRGIPNSISI | P08170 [808-839] | 3531.8846 | 1.83.E+05 |
| P04405 | Glycinin G2 | DDDEEEQPQCVETDKGCQRQSKRS | P04405 [275-298] | 2924.2381 | 1.68.E+05 |
| P04776 | Glycinin G1 | DTPMIGTLAGANSLLNALPEEVIQHTFNLKSQQARQ | P04776 [438-473] | 3964.0233 | 1.40.E+05 |
