# Supplementary material for: Detection of bovine milk–, but likely not soy–derived, peptides in human milk after maternal consumption of bovine milk and soy beverage: a randomized, cross-over, dietary intervention trial
Source: Front Nutr. 2025 Oct 23;12:1642177. doi: 10.3389/fnut.2025.1642177 (PMC12590503; doi:10.3389/fnut.2025.1642177)
Supplement: Supplementary file 3 [file Table_3.pdf]

PARTICIPANT#: __________________ (To Be Completed by Researcher)

BREASTMILK AND FOOD ALLERGEN STUDY
DAILY DIETARY SURVEY

Today's Date: _____ / _____ / 20_____

If you are removing cow's milk products at this time, please only fill out the cow's milk section (you can consume soy products). If you are removing soy products at this time, please only fill out the soy product section (you can consume cow's milk products).

Please mark if, to your knowledge, you have consumed any of the following foods or food ingredients in the previous 24 hours.

Cow's Milk/Dairy

_____ Butter

_____ Buttermilk

_____ Casein/Caseinate

_____ Cheese (all kinds, includes cottage cheese and cream cheese)

_____ Cream (heavy, light, sour, whipping, whipped)

_____ Curds

_____ Custard

_____ Galactose

_____ Ghee

_____ Goat milk products

_____ Half & Half

_____ Hydrolysates (casein hydrolysate, milk protein hydrolysate, protein hydrolysate, whey hydrolysate, whey protein hydrolysate)

_____ Ice cream

_____ Kosher Dairy

_____ Lactalbumin

_____ Lactate Solids

_____ Lactitol Monohydrate

_____ Lactoferrin

_____ Lactoglobulin

_____ Milk (includes nonfat/skim, lowfat, reduced fat, whole, evaporated, condensed, dry, and lactose free)

_____ Milk Chocolate (or other chocolates containing milk ingredients/products)

_____ Nougat

_____ Paneer

_____ Pudding

_____ Racaldent

_____ Rennet

_____ Sheep products

_____ Simplesse

_____ Whey

_____ Yogurt (includes kefir and frozen yogurt)

Soy

_____ Edamame

_____ Bean Curd

_____ Fish Sauces Containing Soy Sauce

_____ Kinako

_____ Koya Dofu

_____ Miso

_____ Natto

_____ Nimame

_____ Okara

_____ Soy (cheese, flour, milk, nuts, sauce, yogurt, ice cream, etc.)

_____ Soybean

_____ Soya

_____ Soybean Oil

_____ Soy Lecithin

_____ Soy Protein

_____ Shoyu

_____ Tamari

_____ Tempeh

_____ Teriyaki Sauce

_____ Worcester Sauce Containing Soy Sauce

_____ Textured Vegetable Protein

_____ Tofu

_____ Yaki-dofu

_____ Yuba

_____ **To my knowledge, I have not consumed any of the above foods over the previous 24 hours.**

In the past 24 hours, have you eaten foods from outside of your home and were unable to identify potential cow's milk or soy ingredients in the food? (for example, food from a restaurant or that another individual made)

☐ Yes ☐No

If YES, please list/describe what you ate with as much detail as you can and where you ate it from:

____

____

____

____

____

____

____

____

____

____

____

In the past 24 hours, did your infant consume any foods or beverages other than breastmilk?

☐ Yes ☐ No

If YES, please list/describe what your infant ate or drank in the last 24 hours:

____

____

____

____

____

____

____

____

____
